# Burden of NASH related liver cancer from 1990 to 2021 at the global, regional, and national levels

**DOI:** 10.3389/fnut.2025.1510563

**Published:** 2025-01-27

**Authors:** Shuang Li, Shuangjiang Li, Linjing Guan, Mingjuan Li, Jiahui Zhao, Min Wu, Qiuyun Li, Hui Li, Guoqing Ouyang, Guangdong Pan

**Affiliations:** ^1^Graduate School of Guangxi University of Chinese Medicine, Nanning, Guangxi, China; ^2^Department of General Surgery, Liuzhou People’s Hospital, Liuzhou, Guangxi, China; ^3^Department of Abdomen Ultrasound, Nanning Sixth People’s Hospital, Nanning, Guangxi, China; ^4^Department of Respiratory Medicine, The First Hospital of Changsha, Changsha, Hunan, China

**Keywords:** incidence, death, disability-adjusted life years, age-standardized rate, NASH-related liver cancer

## Abstract

**Background:**

The global burden of non-alcoholic steatohepatitis (NASH)-related liver cancer (NRLC) is increasing, making NASH the fastest-growing cause of liver cancer worldwide. This study presents a comprehensive analysis of NRLC burden at the global, regional, and national levels, further categorized by age, sex, and sociodemographic index (SDI).

**Method:**

Data on NRLC from the Global Burden of Disease, Injuries, and Risk Factors (GBD) study 2021 were downloaded at global, regional, and national levels. The numbers and age-standardized rates (ASRs) of incidence, mortality, and disability-adjusted life years (DALYs) were analyzed to quantify the global burden of NRLC. Additionally, percentage changes in ASRs were used to identify trends in NRLC from 1990 to 2021.

**Results:**

Globally, both the number of cases and ASRs for NRLC increased between 1990 and 2021. In 2021, there were 42,291 new cases, 40,925 deaths, and 995,475 DALYs attributed to NRLC. East Asia, South Asia, and Southeast Asia reported the highest absolute case numbers, while Western, Southern, and Eastern Sub-Saharan Africa exhibited the highest ASRs. From 1990 to 2021, Australasia, Southern Latin America, and High-income North America showed the most significant increases in NRLC incidence. Nationally, Mongolia, Gambia, and Mozambique exhibited the highest ASR in 2021.The greatest percentage increases in ASIR occurred in Australia, the United Kingdom, and New Zealand between 1990 and 2021. NRLC incidence rates were higher in men and increased with age, peaking at 80–89 years. Similar patterns were observed for NRLC-related deaths and DALYs. Regionally, ASRs initially declined but then increased as SDI rose. At the national level, ASRs consistently decreased with higher SDI.

**Conclusion:**

This study highlights the substantial burden of NRLC at global, regional, and national levels. Males and older individuals bear a higher disease burden, and considerable variation exists across different regions and countries. These findings provide critical insights for formulating effective strategies to prevent and manage NRLC.

## 1 Introduction

Liver cancer ranks as the sixth most common cancer globally and is the third leading cause of cancer-related deaths, according to the analysis of 2020. The incidence and mortality rates of liver cancer are 2–3 times higher in males than in females (1). The burden of liver cancer is most prominent in regions undergoing transitions, including East Asia, Micronesia, and North Africa. Although the morbidity and mortality of liver cancer have decreased in some Southeast Asian countries, such as Japan, China, and South Korea, they have risen in most other regions, including the USA, Australia, and much of Europe (2). Primary liver cancer is primarily divided into hepatocellular carcinoma (HCC), accounting for 85–90% of cases (3), and intrahepatic cholangiocarcinoma (ICC), comprising 10–15%, along with other less common types. Liver cancer is often diagnosed at an advanced stage, complicating treatment efforts. Available treatments range from localized options, such as surgical resection, transplantation, ablation, and radiation, to systemic therapies like chemotherapy and immunotherapy (4). Although these treatments are effective, they come with limitations and potential side effects.

HCC is mainly linked to chronic hepatitis B (HBV) or hepatitis C (HCV) infections, as well as risk factors like aflatoxin exposure, excessive alcohol intake, obesity, type 2 diabetes, smoking, and nonalcoholic steatohepatitis (NASH) (5, 6). While HBV and HCV remain the primary causes of liver cancer, the global burden related to these viruses has decreased due to increased vaccination coverage and the availability of antiviral therapies (7). On the other hand, NASH has rapidly become a leading cause of liver cancer, particularly in regions experiencing rising obesity rates (7). NASH is now the second most common reason for liver transplantation in the United States and contributed significantly to the increase in hepatocellular carcinoma cases by 2000 (8). Although NASH-related liver cancer (NRLC) is not yet the most common cause of liver cancer globally, the growing prevalence of NASH is expected to increase its incidence. Consequently, strategies aimed at preventing liver cancer should focus on managing NASH. Key preventive measures include lifestyle changes such as weight loss, dietary modifications, and increased physical activity. Pharmacological treatments, such as insulin sensitizers, antioxidants, GLP-1 receptor agonists, and bile acid derivatives, have shown promise in clinical trials. In severe cases, bariatric surgery may be a viable option (9, 10). However, these treatments are primarily designed to address NASH itself. To develop comprehensive strategies, understanding the burden of NRLC is essential for informing policy decisions.

In this study, we assessed the global burden of NRLC, analyzing morbidity, mortality, and DALYs data from the GBD database by sex, age, region, country, and SDI. Age-standardized rates (ASRs) were employed to eliminate the confounding effects of differing age structures across regions and countries. Our study aims to support the development of strategies to tackle the growing challenges of NRLC.

## 2 Method

### 2.1 Data source

Our data on NRLC was enrolled from the GBD 2021 study, which synthesized epidemiological information for over 371 diseases and injuries across 204 countries and regions from 1990 to 2021 (11). These 204 countries and territories were grouped into 21 regions and seven super-regions based on geographic location. Additionally, the regions were classified into five Socio-demographic Index (SDI) categories: low, low-middle, middle, high-middle, and high. To estimate the burden of liver cancer, a comprehensive range of data sources was utilized, including peer-reviewed literature, population surveys, national census records, disease surveillance systems, vital registration systems, and other health-related datasets provided by Institute of Health Metrics and Evaluation (IHME) (11). The burden for NLRC were analyzed using DisMod-MR 2.1, a Bayesian meta-regression framework designed to integrate heterogeneous datasets. For regions with insufficient raw epidemiological data, the tool estimates these metrics by utilizing a hierarchical cascade approach within the Global GBD framework. The Socio-demographic Index (SDI) is a composite measure derived from three key indicators: g lag-distributed income (LDI) per capita, average years of education for individuals aged 15 and older, and fertility rates among women under 25 years old. The SDI scores range from 0, indicating the lowest level of development, to 1, representing the highest level (12). The detailed methodology for estimation, conducted by IHME, has been described previously (11, 12). All data used in the present study on incidence, DALYs, deaths, age-standardized incidence rate (ASIR), age-standardized mortality rate (ASMR), and age-standardized DALY rate (ASDR) for liver cancer due to NASH from 1990 to 2021 was extracted using the Global Health Data Exchange (GHDx) query tool (https://ghdx.healthdata.org/gbd-2021/sources).

### 2.2 Statistical analysis

Morbidity, mortality, and DALYs were employed to assess trends in NRLC. Age-standardized rates (ASR) were crucial for comparing morbidity, mortality, and DALYs, as they minimize the impact of age variations across regions or countries with different population structures (13). The percentage change in ASIR, ASMR, and ASDR was calculated to quantify trends from 1990 to 2019 in the global burden of NRLC, which was enrolled from GBD databases. To explore the relationship between ASR of NRLC and SDI at regional and national level, we used the Pearson’s Correlation Coefficient method. The 95% uncertainty intervals (UIs) were calculated by determining the 2.5th and 97.5th percentiles from the ordered draws. All data analyses were conducted in R 4.2.2, and visualization was performed using the “ggplot2” package. Sex differences were examined using an unpaired t-test, with statistical significance set at a *p* value <0.05.

## 3 Results

### 3.1 Global burden of NRLC

**Figure 1:**
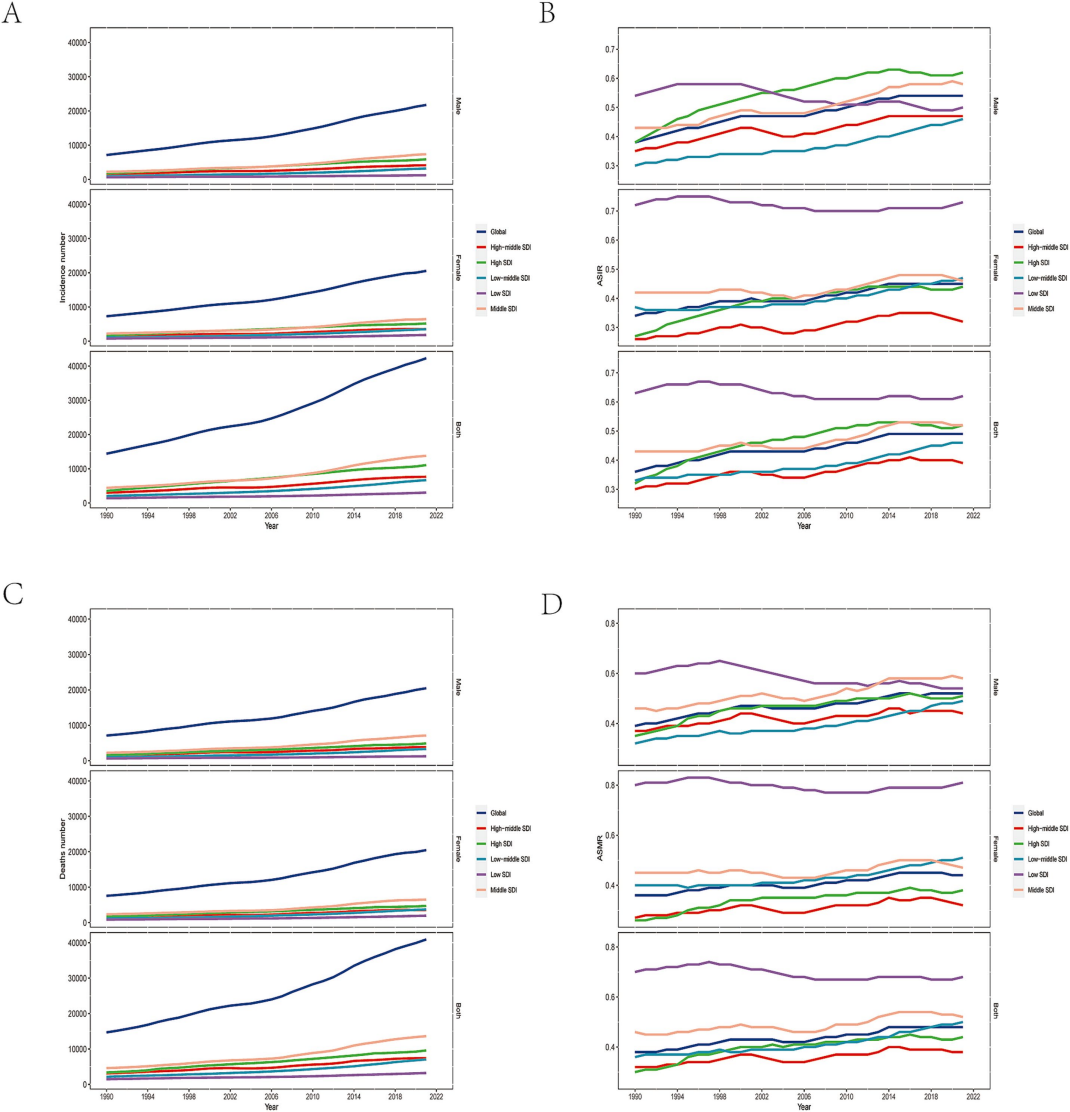
Global burden of NRLC. The global incidence number **(A)** and ASIR **(B)** of NRLC in 2021; The global deaths number **(C)** and ASMR(D) of NRLC in 2021.

The global new cases of NRLC were 42,291 (95% UI: 34,033 to 51,129) in 2021, reflecting a 193.41% increase from 14,414 (95% UI: 11,471 to 17,854) new cases in 1990. The ASIR increased by 36.07%, from 0.36 (95% UI: 0.29 to 0.45) in 1990 to 0.49 (95% UI: 0.40 to 0.60) in 2021 (Figures 1A,B; Table 1). Between 1990 and 2021, the number of deaths of NRLC increased by 178.87%, from 14,675 (95% UI: 11,621 to 18,159) in 1990 to 40,925 (95% UI: 32,961 to 49,610) in 2021. The global ASMR grew from 0.38 (95% UI: 0.30 to 0.47) per 100,000 population in 1990 to 0.48 (95% UI: 0.39 to 0.58) per 100,000 in 2021, representing a 27.78% increase (Figures 1C,D; Table 1). Additionally, the global number of DALYs from NRLC increased by 146.40%, from 404,013 (95% UI: 321,351 to 499,991) in 1990 to 995,475 (95% UI: 808,799 to 1,201,789) in 2021. The ASDR rose by 19.45%, from 9.63 (95% UI: 7.66 to 11.90) to 11.5 (95% UI: 9.39 to 13.84) per 100,000 population (Supplementary Figures S1, S2; Table 1).

**Table 1:** The numbers and age-standardized rates (ASRs) of mortality, incident cases, and disability-adjusted life years (DALYs) for NRLC in 2021 and percentage changes of the ASRs from 1990 to 2021.

	Incidence (95% uncertainty interval)			Mortality (95% uncertainty interval)			DALYs (95% uncertainty interval)		
Location name	Number_2021	ASIR per 100,000 Population (95% UI) in 2021	Percentage change in ASIRs per 100,000 population (95% UI)	Number_2021	ASMR per 100,000 Population (95% UI) in 2021	Percentage change in ASMRs per 100,000 population (95% UI)	Number_2021	ASDR per 100,000 Population (95% UI) in 2021	Percentage change in ASDRs per 100,000 population (95% UI)
Global	42,291 (34,033 to 51,129)	0.49 (0.4 to 0.6)	36.07 (19.14 to 53.43)	40,925 (32,961 to 49,610)	0.48 (0.39 to 0.58)	27.78 (11.59 to 44.49)	995,475 (808,799 to 1,201,789)	11.5 (9.39 to 13.84)	19.45 (4.67 to 35.25)
Sex									
Female	20,537 (16,754 to 24,995)	0.45 (0.36 to 0.54)	31.15 (10.5 to 53.07)	20,456 (16,632 to 25,003)	0.44 (0.36 to 0.54)	24.42 (4.84 to 45.64)	474,886 (387,453 to 575,501)	10.48 (8.57 to 12.63)	16.35 (−2.2 to 36.78)
Male	21,754 (17,209 to 26,961)	0.54 (0.43 to 0.68)	41.61 (23.15 to 67.65)	20,469 (16,180 to 25,363)	0.52 (0.41 to 0.64)	31.95 (14.57 to 56.62)	520,588 (405,448 to 652,528)	12.59 (9.89 to 15.72)	22.82 (6.35 to 47.59)
High SDI	11,075 (8,723 to 13,793)	0.52 (0.42 to 0.65)	63.44 (52.51 to 73.48)	9,585 (7,410 to 11,975)	0.44 (0.35 to 0.55)	44.82 (34.73 to 54.69)	189,918 (152,357 to 237,336)	9.62 (7.83 to 11.98)	31.89 (21.76 to 40.97)
High-middle SDI	7,675 (6,044 to 9,290)	0.39 (0.31 to 0.47)	29.65 (9 to 53.44)	7,438 (5,952 to 9,089)	0.38 (0.3 to 0.46)	19.58 (1.01 to 40.64)	176,293 (139,425 to 212,738)	9.11 (7.2 to 10.93)	8.96 (−9.61 to 29.86)
Low SDI	3,042 (2,156 to 4,185)	0.62 (0.43 to 0.85)	−2.83 (−22.38 to 30.88)	3,222 (2,283 to 4,455)	0.68 (0.48 to 0.94)	−2.34 (−22.55 to 30.96)	91,453 (66,083 to 124,972)	16.13 (11.52 to 22.31)	−4.47 (−23.08 to 27.62)
Low-middle SDI	6,690 (5,329 to 8,245)	0.46 (0.37 to 0.58)	39.27 (8.37 to 72)	7,040 (5,612 to 8,681)	0.5 (0.4 to 0.62)	38.69 (7.91 to 71.94)	193,460 (154,628 to 237,210)	12.55 (10.02 to 15.42)	36.56 (8.69 to 68.8)
Middle SDI	13,783 (10,897 to 16,783)	0.52 (0.41 to 0.63)	21.6 (1.67 to 44.64)	13,613 (10,818 to 16,683)	0.52 (0.42 to 0.64)	14.07 (−4.3 to 35.45)	343,704 (271,248 to 417,417)	12.49 (9.94 to 15.18)	6.86 (−10.19 to 26.45)
Andean Latin America	192 (125 to 276)	0.33 (0.22 to 0.48)	27.55 (−3.36 to 64.66)	213 (139 to 306)	0.37 (0.24 to 0.53)	27.52 (−3.52 to 64.71)	4,624 (3,034 to 6,605)	7.8 (5.09 to 11.18)	19.78 (−9.09 to 54.66)
Australasia	322 (231 to 437)	0.61 (0.44 to 0.83)	299.94 (247.07 to 360.96)	297 (211 to 404)	0.55 (0.39 to 0.74)	257.98 (208.99 to 314.34)	6,245 (4,495 to 8,411)	12.66 (9.28 to 16.87)	238.39 (193.69 to 290.06)
Caribbean	112 (76 to 155)	0.21 (0.14 to 0.29)	9.13 (−6.07 to 25.84)	121 (82 to 167)	0.22 (0.15 to 0.31)	6.74 (−8.26 to 22.99)	2,762 (1904 to 3,764)	5.15 (3.55 to 6.97)	8.09 (−7.57 to 25.12)
Central Asia	447 (299 to 645)	0.56 (0.38 to 0.8)	12.9 (−4.14 to 35.72)	474 (318 to 679)	0.61 (0.41 to 0.87)	14.44 (−3.07 to 37.61)	12,598 (8,557 to 18,122)	14.71 (10.1 to 20.96)	6.62 (−9.77 to 29)
Central Europe	610 (431 to 823)	0.27(0.19 to 0.36)	8.14(−3.51 to 22.13)	669(475 to 908)	0.29(0.21 to 0.39)	5.97(−5.62 to 19.74)	13,720(9,793 to 18,559)	6.43(4.73 to 8.69)	2.62(−9.45 to 16.61)
Central Latin America	739(585 to 927)	0.3(0.24 to 0.38)	27.06(13.16 to 40.7)	803(631 to 1,014)	0.33(0.26 to 0.42)	26.22 (12.33 to 39.57)	18,874 (14,895 to 23,204)	7.47 (5.91 to 9.2)	23.42 (10.13 to 37.27)
Central Sub-Saharan Africa	224 (93 to 504)	0.41 (0.16 to 0.99)	−16.12 (−39.26 to 14.7)	233 (96 to 535)	0.46 (0.18 to 1.12)	−15.08 (−38.78 to 17.04)	7,222 (3,040 to 16,482)	11.1 (4.55 to 25.62)	−18.34 (−41.31 to 13.14)
East Asia	11,858 (9,095 to 15,058)	0.55 (0.43 to 0.69)	15.41 (−10.03 to 46.58)	10,936 (8,458 to 13,798)	0.51 (0.4 to 0.64)	2.15 (−20.04 to 29.04)	268,803 (205,739 to 340,213)	12.38 (9.61 to 15.55)	−7.71 (−28.83 to 19.52)
Eastern Europe	735 (615 to 864)	0.21 (0.18 to 0.25)	62.07 (49.48 to 76.72)	796 (668 to 935)	0.23 (0.19 to 0.27)	62.27 (49.56 to 77.44)	18,092 (15,174 to 21,280)	5.45 (4.65 to 6.34)	52.53 (40.61 to 67.16)
Eastern Sub-Saharan Africa	1,193 (815 to 1,651)	0.73 (0.5 to 1)	4.81 (−18.97 to 42.18)	1,261 (865 to 1739)	0.8 (0.55 to 1.11)	5.3 (−19.14 to 42.92)	36,288 (24,652 to 49,884)	18.91 (12.89 to 26.16)	2.65 (−19.94 to 39.54)
High-income Asia Pacific	3,167 (2,414 to 4,058)	0.65 (0.5 to 0.84)	−17.74 (−30.9 to −4.7)	2,490 (1864 to 3,212)	0.48 (0.37 to 0.62)	−32.43 (−42.99 to −21.34)	43,135 (32,958 to 56,348)	9.88 (7.67 to 12.91)	−43.52 (−52.26 to −33.55)
High-income North America	3,904 (3,250 to 4,642)	0.59 (0.5 to 0.7)	163.67 (153.52 to 173.98)	3,302 (2,726 to 3,914)	0.49 (0.41 to 0.58)	137.31 (127.91 to 146.33)	69,421 (58,562 to 82,160)	10.98 (9.33 to 12.85)	136.91 (128.06 to 146.4)
North Africa and Middle East	2,859 (2021 to 3,924)	0.64 (0.44 to 0.87)	60.68 (6.79 to 122.43)	2,966 (2066 to 4,066)	0.69 (0.47 to 0.94)	57.58 (4.09 to 118.42)	79,893 (56,400 to 108,507)	16.43 (11.62 to 22.51)	55.25 (4.5 to 114.04)
Oceania	24 (14 to 45)	0.33 (0.19 to 0.58)	−8.91 (−33.32 to 31.27)	25 (14 to 46)	0.36 (0.2 to 0.62)	−9.71 (−34.41 to 30.26)	750 (429 to 1,362)	8.83 (5.02 to 16.23)	−10.74 (−34.91 to 29.99)
South Asia	5,159 (4,336 to 6,147)	0.35 (0.29 to 0.42)	56.34 (34.63 to 81.46)	5,469 (4,600 to 6,486)	0.38 (0.32 to 0.45)	57.84 (36.07 to 83.29)	146,108 (124,852 to 173,247)	9.34 (7.93 to 11.07)	48.87 (28.93 to 71.79)
Southeast Asia	4,174 (2,922 to 5,708)	0.65 (0.45 to 0.89)	16.12 (−14.05 to 47.17)	4,267 (3,013 to 5,833)	0.69 (0.48 to 0.94)	13.45 (−16.44 to 44.36)	113,299 (78,517 to 156,617)	16.41 (11.55 to 22.39)	8.06 (−18.94 to 37.36)
Southern Latin America	161 (106 to 226)	0.18 (0.12 to 0.26)	182.5 (140.51 to 226.59)	171 (113 to 242)	0.19 (0.13 to 0.27)	173.7 (133.31 to 214.6)	3,727 (2,564 to 5,189)	4.32 (2.99 to 5.92)	167.57 (127.17 to 210.28)
Southern Sub-Saharan Africa	590 (466 to 729)	1.04 (0.83 to 1.3)	71.25 (2.58 to 187.64)	623 (492 to 769)	1.15 (0.92 to 1.42)	72.68 (3.47 to 189.41)	17,479 (13,944 to 21,666)	27.81 (22.24 to 34.31)	64.14 (−0.62 to 175.78)
Tropical Latin America	392 (330 to 457)	0.15 (0.13 to 0.18)	28.46 (21.93 to 35.59)	423 (356 to 493)	0.17 (0.14 to 0.19)	26.94 (20.51 to 34.02)	10,234 (8,694 to 11,822)	3.96 (3.36 to 4.56)	25.13 (18.52 to 31.94)
Western Europe	3,333 (2,440 to 4,489)	0.35 (0.26 to 0.46)	78.08 (66.02 to 90.7)	3,159 (2,255 to 4,272)	0.32 (0.23 to 0.42)	56.34 (45.54 to 67.42)	59,838 (44,237 to 78,710)	6.95 (5.25 to 9.12)	51.92 (42.66 to 62.87)
Western Sub-Saharan Africa	2098 (1,559 to 2,792)	1.11 (0.83 to 1.48)	−4.11 (−36.25 to 46.91)	2,227 (1,654 to 2,944)	1.23 (0.91 to 1.65)	−3.25 (−36.03 to 48.16)	62,362 (46,160 to 83,237)	28.33 (20.95 to 37.63)	−7.3 (−37.73 to 40.56)

### 3.2 Regional burden of NRLC

**Figure 2:**
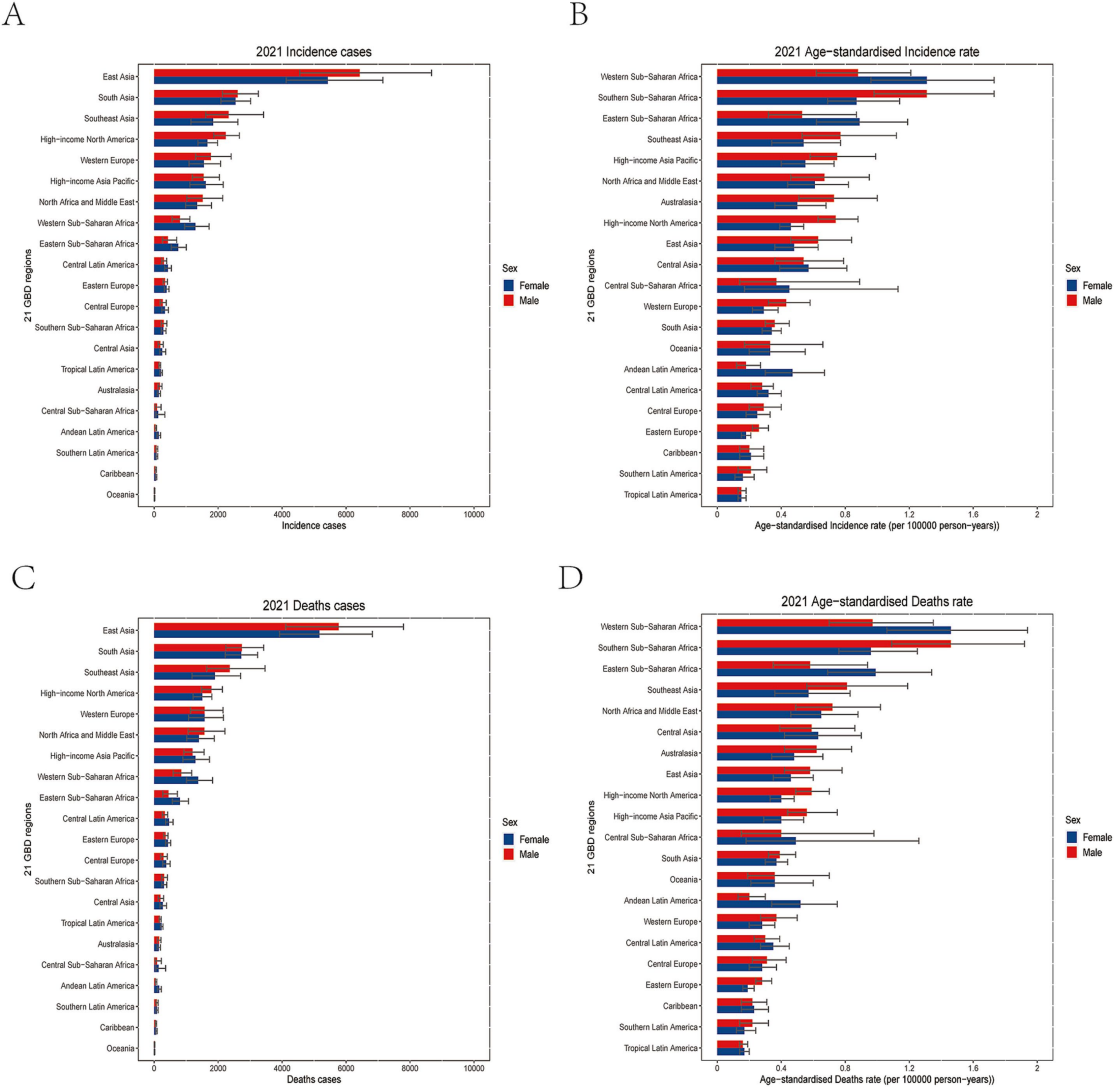
Regional burden of NRLC. The regional level incidence number **(A)** and ASIR **(B)** of NRLC in 2021; The regional level deaths number. **(C)** and ASMR **(D)** of NRLC in 2021.

In 2021, East Asia recorded the highest number of new cases of NRLC with 11,858 (95% UI: 9,095 to 15,057), followed by South Asia and Southeast Asia (Figure 2A; Table 1). The highest ASIRs were found in Western Sub-Saharan Africa (1.11 [95% UI: 0.83 to 1.48] per 100,000), followed by Southern Sub-Saharan Africa, and Eastern Sub-Saharan Africa (Figure 2B; Table 1). In terms of deaths and DALYs, the East Asia, South Asia, and Southeast Asia were also the top three regions with the highest numbers of death and DALYs (Figure 2C; Supplementary Figure S3; Table 1). The highest ASMRs and ASDRs were also seen in Western Sub-Saharan Africa, followed by Southern Sub-Saharan Africa, and Eastern Sub-Saharan Africa (Figure 2D; Supplementary Figure S4; Table 1).

From 1990 to 2021, most regions experienced an increase in ASR for incidence, deaths, and DALYs. Australasia [300.00% (95% UI: 247.10 to 360.96%)], Southern Latin America, and High-income North America exhibited the largest increases in ASIR, ASMR, and ASDR, while High-income Asia Pacific [−17.74% (95% UI: −30.90% to −4.70%)], Central Sub-Saharan Africa, and Oceania saw the greatest decreases in ASIR, ASMR, and ASDR (Table 1).

### 3.3 National burden of NRLC

**Figure 3:**
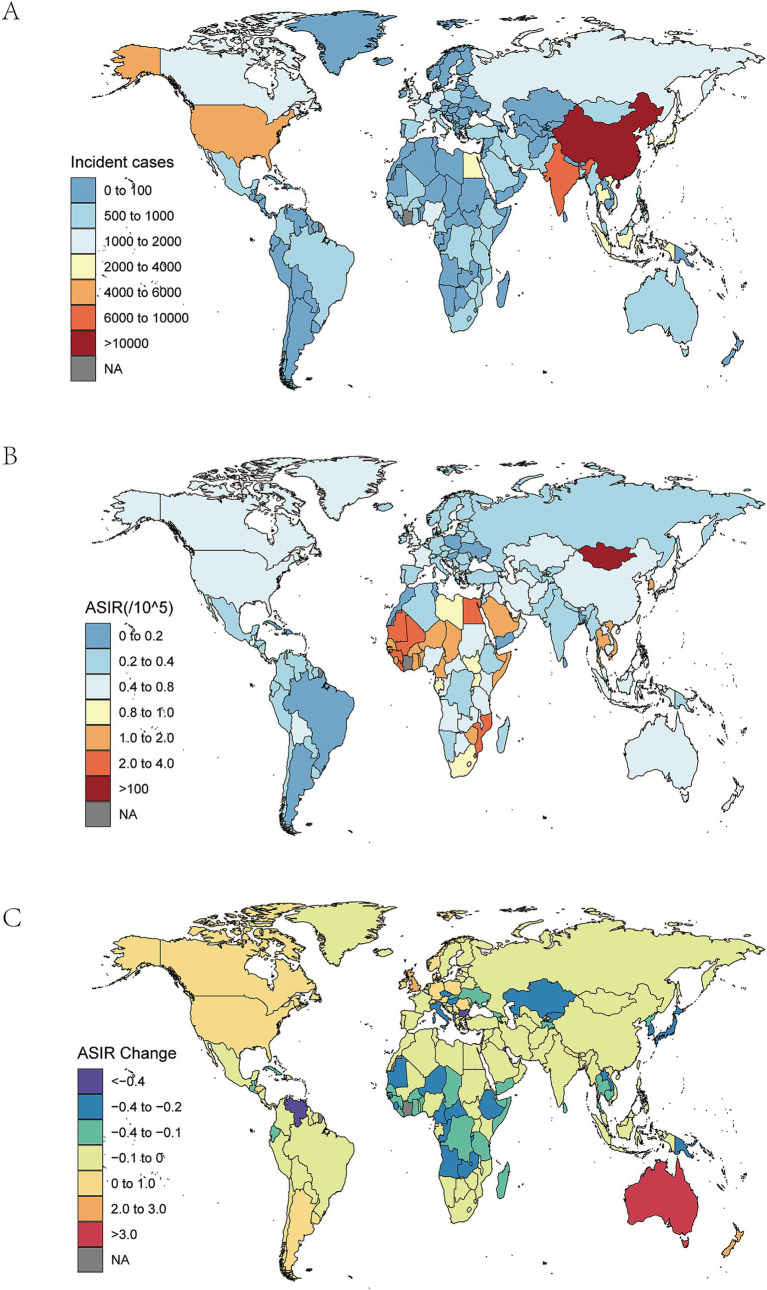
National burden of NRLC. **(A)**The national level incident cases of NRLC in 2021; **(B)** The national level ASIR of NRLC in 2021; **(C)** The national level changes of ASIR of NRLC from 1990 to 2021.

In 2021, China, India, and the United States had the highest number of new cases of NRLC, with China leading at 11,293 cases (95% UI 8,663–14,314), followed by India and the United States (Figure 3A; Supplementary Table S1). These three countries also reported the highest numbers of deaths and DALYs (Supplementary Figures S5, S6; Supplementary Tables S2, S3).

Countries with the highest ASIRs in 2021 were Mongolia (5.26 per 100,000; 95% UI 3.28–8.13), followed by Gambia, and Mozambique (Figure 3B; Supplementary Table S1). In contrast, the countries with the lowest ASIRs included Morocco (0.06; 95% UI 0.04–0.09), Mauritius, and Argentina (Figure 3B; Supplementary Table S1). Mongolia also had the highest ASMR of NRLC (5.87 per 100,000; 95% UI 3.61–9.12), followed by Gambia and Mozambique (Supplementary Figure S7; Supplementary Table S2). The lowest ASMRs were observed in Morocco (0.06; 95% UI 0.03–0.09), the Mauritius, and Argentina (Supplementary Figure S7; Supplementary Table S2). Regarding ASDRs, Mongolia, Gambia, and Mozambique had the highest figures (Supplementary Figure S8; Supplementary Table S3), respectively. The countries with the lowest ASDRs were the Morocco (10.56; 95% UI 5.91–17.00), Mauritius, and Argentina (Supplementary Figure S8; Supplementary Table S3).

From 1990 to 2021, the greatest percentage increases in ASIR occurred in Australia (326.88%; 95% UI 260.30–406.06%), the United Kingdom, and New Zealand (Figure 3C; Supplementary Table S1). On the other hand, the largest declines were seen in Mauritius (−79.12%; 95% UI -81.52% to −76.71%), Bulgaria, and Kuwait (Figure 3C, Supplementary Table S1). Similarly, Australia, the United Kingdom, and Lesotho saw the largest increases in ASMR between 1990 and 2021 (Supplementary Figure S9; Supplementary Table S2). The most significant reductions in ASMR also occurred in Mauritius, Bulgaria, and Kuwait (Supplementary Figure S9; Supplementary Table S2). The greatest rises in ASDR were recorded in Australia (260.26%; 95% UI 204.89–325.35%), Lesotho, and the United Kingdom (Supplementary Figure S10; Supplementary Table S3). Meanwhile, the sharpest declines in ASDR were seen in Mauritius, Kuwait, and South Korea (Supplementary Figure S10; Supplementary Table S3).

### 3.4 Burden of NRLC by age and sex

**Figure 4:**
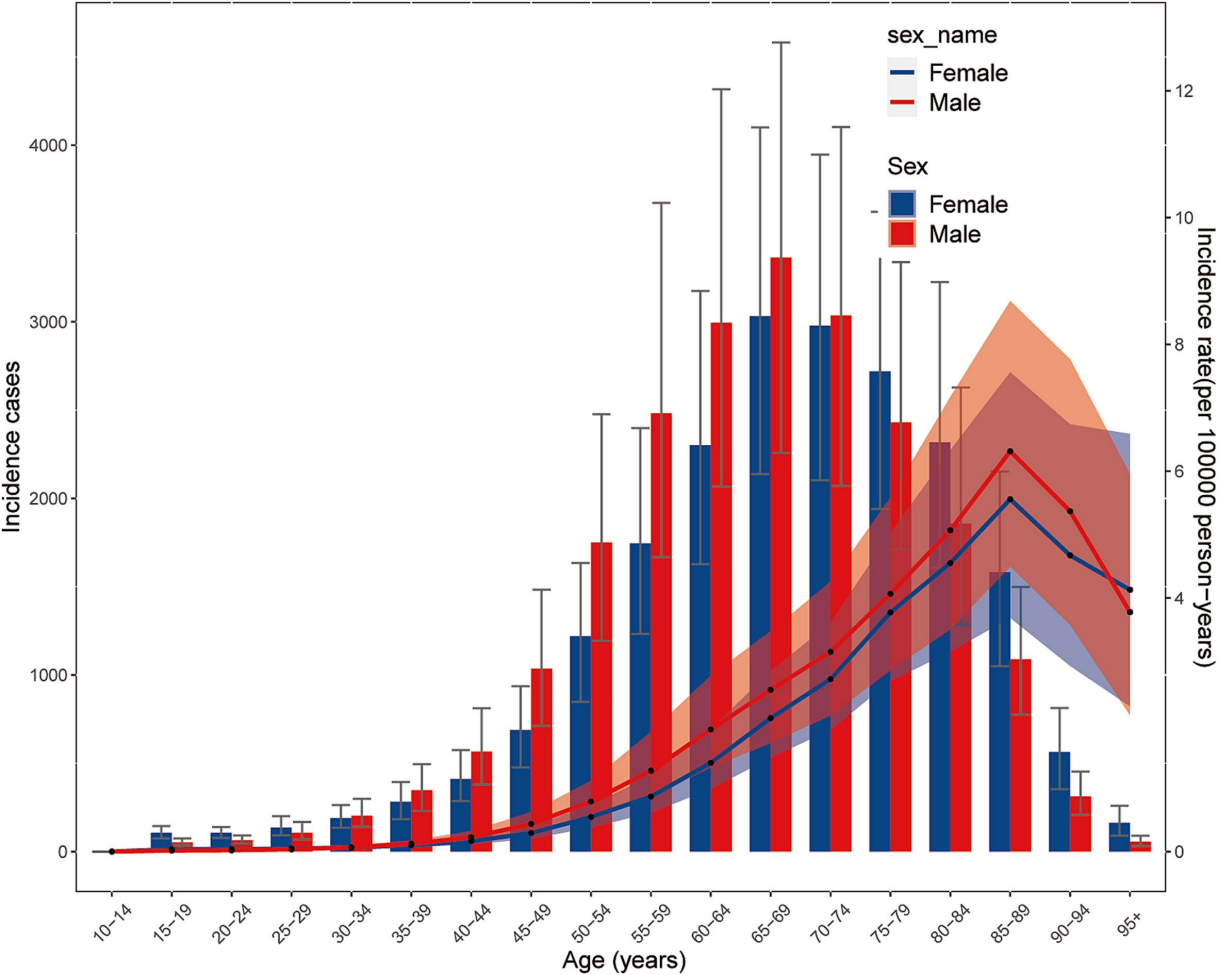
Burden of NRLC by age and sex. The incidence cases number and rates in different level of age and sex.

In 2021, global incidence rates of NRLC increased with age, peaking at 85–89 years for both women [5.56 (95% UI 3.69 to 7.56)] and men [6.31 (95% UI 4.50 to 8.69)], before declining after the age of 89. The incidence rate in women exceeded that of men before the age of 30 and after 95, while men had higher rates between 30 and 94. The number of new cases followed a similar pattern, increasing with age until 65–69, then decreasing. Men had more cases than women between 30 and 74, while women had more cases outside this age range (Figure 4A).

In 2021, global mortality rates from NRLC were highest between ages 90–94 for both women [6.48 (95% UI 4.07 to 9.35)] and men [7.46 (95% UI 4.95 to 10.78)], with mortality increasing before 90 and declining after 94. Mortality rates in women were lower than men between 35 and 94, but higher at other ages. The number of deaths mirrored this trend, increasing with age until 65–69, then decreasing. Men had higher death counts than women between 30 and 69, while women had more deaths after age 70 (Supplementary Figure S11).

DALY rates also varied by age. Both men and women saw increases in DALYs rates until 80–84 years, followed by a decrease after 84. The highest DALYs rate for men was 71.03 (95% UI 49.12 to 100.68), and for women, it was 65.39 (95% UI 45.51 to 90.56). Women had lower DALYs rates than men between 30 and 94, but higher rates at other ages. The number of DALYs reflected the DALYs rate pattern, peaking at 60–64 for men and 65–69 for women, before declining. Between 30 and 69, men had more DALYs, while women surpassed men outside this age range (Supplementary Figure S12).

### 3.5 Burden of NRLC by sociodemographic index

**Figure 5:**
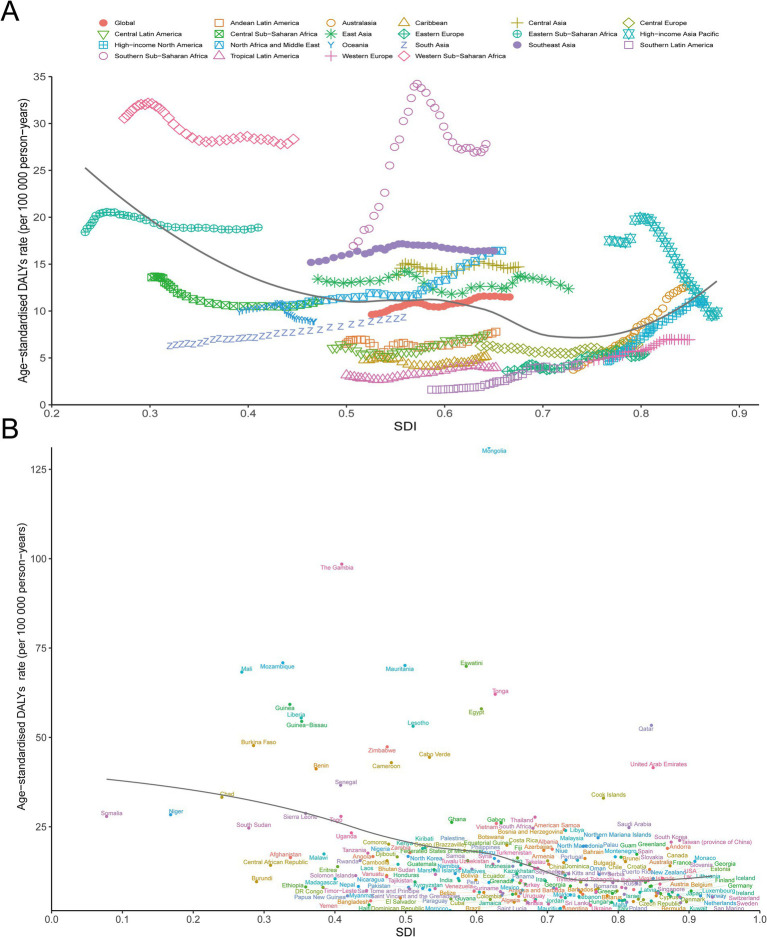
Burden of NRLC by sociodemographic index. **(A)** The association between ASDR and SDI of NRLC of 21 GBD regions from 1990 to 2021; **(B)** The association between ASDR and SDI of NRLC of 204 countries and territory in 2021.

The burden of NRLC by SDI was analyzed regionally between 1990 and 2021, focusing on the correlation between ASDR and SDI. We found that ASDR initially decreased and then increased as SDI rose. Generally, there was a negative correlation between DALYs of NRLC and SDI from 1990 to 2021(*ρ* = −0.383, *p* < 0.05). The lowest SDI point was observed at approximately 0.587. In regions such as Western Sub-Saharan Africa, Southern Sub-Saharan Africa, Southeast Asia, and Central Asia, the observed ASDRs were higher than expected based on their SDI, while Southern Latin America, Tropical Latin America, the Caribbean, Central Europe, Eastern Europe, and South Asia had ASDRs lower than expected (Figure 5A). The patterns of ASIR (Supplementary Figure S13) and ASMR (Supplementary Figure S14) were similar to ASDR.

At the national level, ASDRs of NRLC were generally negatively associated with SDI (*ρ* = −0.379, *p* < 0.05). Countries including The Gambia, Mozambique, Mauritania, Eswatini, and Mali had ASDRs significantly higher than expected, while countries like Brazil, Morocco, Mauritius, and Saint Lucia had ASDRs significantly lower than expected (Figure 5B). The patterns of ASIR (Supplementary Figure S15) and ASMR (Supplementary Figure S16) were similar to ASDR.

## 4 Discussion

This study provides an updated analysis of the global incidence, mortality, and DALYs associated with NRLC from 1990 to 2021. In 2021, approximately 42,290 new cases, 40,930 deaths, and 9.95 million DALYs were reported worldwide. From 1990 to 2021, the ASIR, ASMR, and ASDR of NRLC increased by 36.07, 27.78, and 19.45%, respectively. These findings indicate a significant global rise in NRLC, which is consistent with earlier studies (7, 14). This trend may be linked to the growing global population, as well as advances in diagnostic capabilities and improvements in dietary quality (15). Furthermore, we assessed the effects of age and sex on NRLC in 2021. Notably, this is the first study to investigate the relationship between the SDI and ASIR, ASMR, and ASDR of NRLC across global, regional, and national levels from 1990 to 2021.

Due to the widespread availability of vaccines and antiviral therapies that have led to a decline in HBV and HCV, NASH has become a leading cause of liver cancer, surpassing the declining burden of liver cancer caused by HBV and HCV. This shift has contributed to the fastest increase in liver cancer rates (7, 16, 17). Previous study reported that nearly 15% of liver cancer was attributed to NASH (18). NASH, which progress from NAFLD, is characterized by inflammation, liver injury, and fibrosis (19). Metabolic conditions, such as diabetes mellitus (DM) and obesity, are the two primary risk factors for NASH (19, 20). Previous studies have reported a significant rise in the global prevalence and incidence of NAFLD in recent years. Between 1990 and 2019, the prevalence of NAFLD exceeded 9 million individuals aged over 30 years (21). Moreover, the increased incidence of NAFLD has been linked to the growing incidence of metabolic syndrome, insulin resistance, and DM (22, 23). Our study found that ASIR of NRLC significantly increased by 36.07% from 1990 to 2021, consistent with previous findings that NRLC incidence has been rising steadily since 1990 (24). Additionally, previous research has shown that DM accounts for approximately 7.59% of all DALYs and 8.76% of NRLC-related deaths due to high fasting plasma glucose (16). Therefore, weight loss become the only evidence-based method to prevent or delay the progression from NRLC (25).

Our study identified significant regional disparities in the burden of NRLC across the 21 GBD regions. In 2021, Western and Southern Sub-Saharan Africa reported the highest ASR of NRLC. These trends are likely driven by increasingly sedentary lifestyles, evolving dietary habits, and the widespread introduction of antiretroviral therapy (ART) for HIV. These factors have likely contributed to the rise in obesity and diabetes in Sub-Saharan Africa between 2000 and 2014 (26). Therefore, in regions with a high NRLC burden, efforts should focus on reducing the incidence of NAFLD/NASH through dietary modifications, weight loss, and increased physical activity. Additionally, it is crucial to increase funding in Sub-Saharan Africa to manage type 2 diabetes, hypertension, and dyslipidemia, which are significant contributors to NAFLD (21). Although Southern Latin America had the second-lowest burden of NRLC in 2021, it experienced the second-largest increase from 1990 to 2021. This is consistent with reports indicating that Southern Latin America, particularly Argentina, has the lowest prevalence of NAFLD (21, 27, 28). Additionally, limited healthcare infrastructure in this region, which leads to less frequent early histological evaluation and treatment, is a critical factor contributing to the increasing burden (29). NAFLD is strongly linked to various metabolic disorders, including type 2 diabetes, obesity, metabolic syndrome, hypertension, and hyperlipidemia. As a result, its incidence is expected to rise alongside the growing prevalence of obesity and type 2 diabetes (27). Previous studies have reported that globalization and the adoption of Western dietary habits have contributed to rising obesity and chronic disease rates in Southern Latin America, which are major risk factors for NASH. Therefore, the increase in NRLC cases in this region may be closely related to lifestyle changes (27, 28, 30, 31). Therefore, greater attention and effort are needed in Southern Latin America to mitigate this rising trend.

In 2021, the countries with the highest numbers of new cases, deaths, and DALYs of NRLC were China, India, and the United States. This can be attributed to their large populations (31). Additionally, environmental contamination from heavy metals, primarily stemming from industrial and agricultural activities in these countries, is an important factor contributing to the progression of NAFLD to liver cancer through mechanisms related to inflammation and insulin resistance (32). Our study also identified Mongolia, Gambia, and Mozambique as having the highest ASRs of NRLC in 2021. Similarly, Batsaikhan O reported that Mongolia had the highest ASMR of liver cancer globally in 2019 (33). This situation may be linked to Mongolia’s high-fat diet, which is predominantly composed of meat and dairy products, contributing to the development of NRLC. In contrast, the Mediterranean diet, rich in fruits, vegetables, and healthy fats, has been shown to reduce NAFLD progression and is less commonly adopted in regions with high NRLC rates (34). Therefore, regulators should focus on expanding and fortifying food supplies and promoting healthier food options, while implementing national surveillance systems to promote healthier diets rich in micronutrients, which may help reduce the incidence of chronic diseases (35). Furthermore, sedentary lifestyles and lack of physical activity are key contributors to the incidence of NASH, a major driver of NRLC (36). For instance, the highest increasing changes in the ASRs of NRLC from 1990 to 2021 were observed in the United Kingdom and Australia. Previous studies also indicate that one of the reasons for the increasing trend of NASH in both Australia and the United Kingdom are due to the life of inactivity in these countries (37, 38). Surveys in the UK and Australia indicate that despite growing health awareness, many individuals do not meet the recommended daily physical activity levels due to factors such as high work pressure and fast-paced lifestyles, leading to an increase in obesity, insulin resistance, NAFLD, and other chronic diseases (39–42). Public health data in the UK show that many adults do not meet the recommended weekly amount of physical activity (such as at least 150 min of moderate-intensity exercise per week) (43). Australia faces similar challenges, particularly among older age groups with low physical activity levels (44). Our study found that the incidence, mortality, and DALYs of NRLC generally increased with age, peaking between 80 and 94 years. Previous research has shown that older adults are more likely to develop liver cancer, with advancing age is strongly associated with HCC (45). This is likely due to the longer exposure of older individuals to risk factors for NASH, such as obesity, DM, and other metabolic disorders, all of which are closely linked to the disease (16). Additionally, NASH tends to progress more slowly than HBV and HCV, requiring more time to advance to liver cancer. Furthermore, the aging immune system and declining cellular repair mechanisms may contribute to increased susceptibility to liver cancer (46).

Our study found that the burden of incidence, mortality, and DALYs due to NRLC is generally higher in males than in females. This result aligns with previous research, Global burden of liver cancer in males and females: Changing etiological basis and the growing contribution of NASH (5). Several factors may contribute to the higher burden of NRLC in males. First, hormonal differences between men and women may play a role. Estrogen, which is present at higher levels in women, particularly premenopausal women, has been shown to have anti-inflammatory properties and plays a crucial role in maintaining liver health. Research indicates that premenopausal women are less likely to develop liver inflammation and fibrosis, both of which are precursors to HCC, due to the protective effects of estrogen (47). Estrogen also regulates pathways involved in lipid metabolism and insulin sensitivity, both of which are crucial in the progression of NASH to liver cancer. However, postmenopausal women lose this hormonal protection as estrogen levels decline, increasing the risk of liver disease and HCC (48). This decline in estrogen may explain why, after the age of 75, the incidence, mortality, and DALYs in females surpass those in males. Second, men tend to have a higher prevalence of metabolic syndrome, which includes obesity, insulin resistance, hypertension, and dyslipidemia—conditions that are significant risk factors for both NASH and liver cancer. Studies suggest that men are more prone to accumulating visceral fat, which is metabolically active and contributes to liver inflammation, compared to women, who typically accumulate subcutaneous fat (49). Third, higher rates of alcohol consumption and smoking in men, compared to women, may also contribute to this disparity. Although alcohol consumption and smoking are not direct causes of NASH, they exacerbate liver inflammation and fibrosis in individuals with NASH, increasing the likelihood of liver cancer development (32, 45, 50, 51). In individuals under 30, both males and females likely experience less cumulative exposure to major risk factors for NASH, such as obesity, metabolic syndrome, and insulin resistance. Interestingly, the disease burden in females under 30 tends to be slightly higher than in males. This could be attributed to the role of DHEA and DHEA-S, which begin to increase around the ages of 7 to 9, peak between 20 and 30 years, and then decline. These hormones serve as critical precursors for male androgens and female estrogens, requiring sufficient time to accumulate. The differences between males and females in this age group may be influenced by variations in hormone levels (52). Therefore, in regions with a higher burden of NRLC among men, government policies to limit tobacco and alcohol sales could help reduce the disease burden. Additionally, regular physical activity produces endogenous active mediators with anti-inflammatory effects, which can help prevent NASH-induced liver fibrosis and, subsequently, NRLC (53).

Although Liu et al. (54) reported the burden of NRLC from 1990 to 2019, no previous research has investigated the association between ASR and the SDI across different regions and countries. In our study, we observed a generally negative correlation between SDI and the ASDR of NRLC across the 21 GBD regions from 1990 to 2021, as well as in 204 countries in 2021. This suggests that lower-SDI countries generally bear a higher burden of NRLC. This finding aligns with previous global studies on NAFLD, which demonstrated that incidence, prevalence, mortality, and DALYs were generally negatively associated with SDI (55). It is widely understood that the burden of diseases tends to decrease as SDI increases, due to improvements in medical resources, enhanced health literacy, and the implementation of preventive healthcare measures (16). For example, promoting a balanced diet to reduce the intake of processed and high-sugar foods, alongside physical exercise to reduce the risk of chronic diseases such as cardiovascular disease, diabetes, obesity, and NAFLD (27, 56). In contrast, individuals in lower-income regions often cannot afford nutritious foods like vegetables, fruits, and lean meats, making them more vulnerable to obesity and its associated complications. Moreover, they typically lack access to high-quality healthcare services, such as early diagnosis and advanced treatments, contributing to higher rates of mortality and DALYs in these populations.

This study examined the global and regional burdens and trends of NRLC from 1990 to 2021. However, several limitations should be considered. Firstly, the robustness and accuracy of the present study largely depend on the quality and quantity of data from the GBD 2021 database. The GBD database integrates data from various sources, including national surveys, hospital records, and registries, which may vary in quality and completeness across regions. This heterogeneity could affect the comparability of data, particularly when analyzing trends in low- and middle-income countries. Moreover, in low-income regions, underreporting of disease burden due to limited healthcare infrastructure and incomplete vital registration systems is a well-documented challenge. This limitation may lead to an underestimation of the true burden of NRLC and other conditions. In these regions, where data quality is often compromised by economic constraints, epidemiological information on NRLC may be less reliable, directly impacting the robustness of our findings. Secondly, the coexistence of NASH with other risk factors such as HBV and HCV complicate the identification of NRLC’s primary cause. This presents a challenge in fully assessing NRLC in such cases. This study did not evaluate the influence of differences in diagnostic criteria, preventive strategies, and treatment approaches across various regions and countries. Such variations likely exist, even among countries with similar SDI levels, potentially impacting the findings. Finally, a comprehensive consideration of the COVID-19 pandemic within the broader context of factors contributing to health loss is necessary. Understanding the pandemic’s effects on health outcomes is crucial for interpreting recent trends.

## 5 Conclusion

We found that NRLC has been a significant global health threat. Overall, the global burden of NRLC has increased substantially from 1990 to 2021. The highest incidence, mortality, and DALYs were observed in East Asia, South Asia, and Southeast Asia, while the lowest were reported in Western, Southern, and Eastern Sub-Saharan Africa. Countries with the highest ASRs were Mongolia, Gambia, and Mozambique. The burden of NRLC is generally greater in men than in women, with incidence increasing with age. These findings provide critical insights for policymakers and offer valuable evidence to develop more effective strategies to guide the development of more effective strategies to mitigate the burden of NRLC.

## Data Availability

The original contributions presented in the study are included in the article/Supplementary material, further inquiries can be directed to the corresponding authors.
