# Supplementary material for: Burden of NASH related liver cancer from 1990 to 2021 at the global, regional, and national levels
Source: Front Nutr. 2025 Jan 27;12:1510563. doi: 10.3389/fnut.2025.1510563 (PMC11807830; doi:10.3389/fnut.2025.1510563)
Supplement: Supplementary file 1 [file Data_Sheet_1.pdf]

Additional file 1

Global Burden of NASH related liver cancer from 1990 to 2021 at the global, regional, and national levels

Fig S1: The DALYs number of NRLC at the global and regional levels from 1990 through 2021. 2

Fig S2: The ASDR of NRLC at the global and regional levels from 1990 through 2021. 3

Fig S3: The DALYs cases of NRLC in 2021 for 21 GBD regions, by sex. 4

Fig S4: The ASDR of NRLC in 2021 for 21 GBD regions, by sex. 5

Fig S5: The deaths cases of NRLC in 2021 for 204 countries and territory. 6

Fig S6: The DALYs cases of NRLC in 2021 for 204 countries and territory. 7

Fig S7: The ASMR of NRLC in 2021 for 204 countries and territory. 8

Fig S8: The ASDR of NRLC in 2021 for 204 countries and territory. 9

Fig S9: The change of ASMR from 1990 to 2021 in 204 countries and territory. 10

Fig S10: The change of ASDR from 1990 to 2021 in 204 countries and territory. 11

Fig S11: The effect of SDI to Deaths cases and rate in 2021, for age and sex. 12

Fig S12: The effect of SDI to DALYs cases and rate in 2021, for age and sex. 13

Fig S13: The association between SDI and ASIR from 1990 to 2021 in 21 GBD regions. 14

Fig S14: The association between SDI and ASMR from 1990 to 2021 in 21 GBD regions. 15

Fig S15: The association between SDI and ASIR in 2021 for 204 countries and territory. 16

Fig S16: The association between SDI and ASMR in 2021 for 204 countries and territory. 17

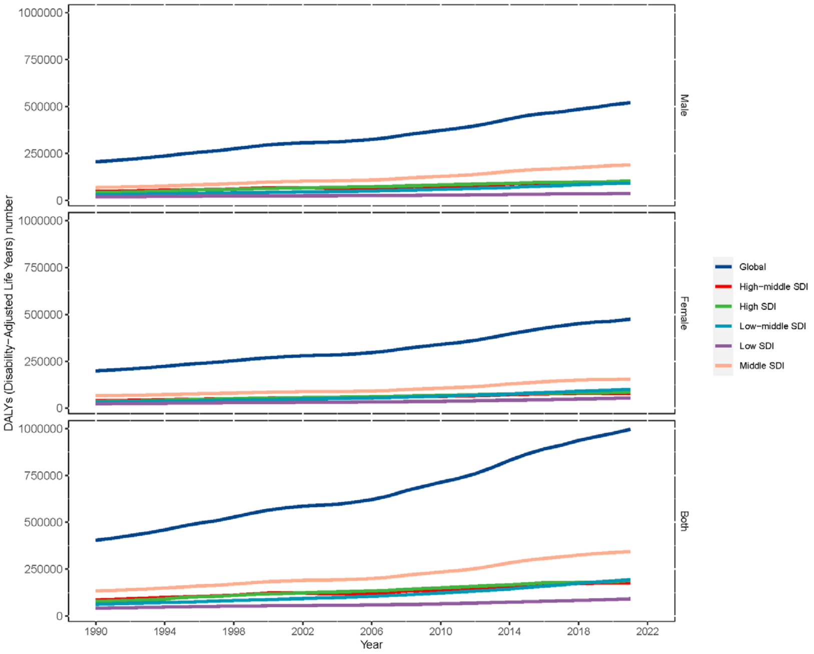

Figure S1. The DALYs number of NRLC at the global and regional levels from 1990 through 2021

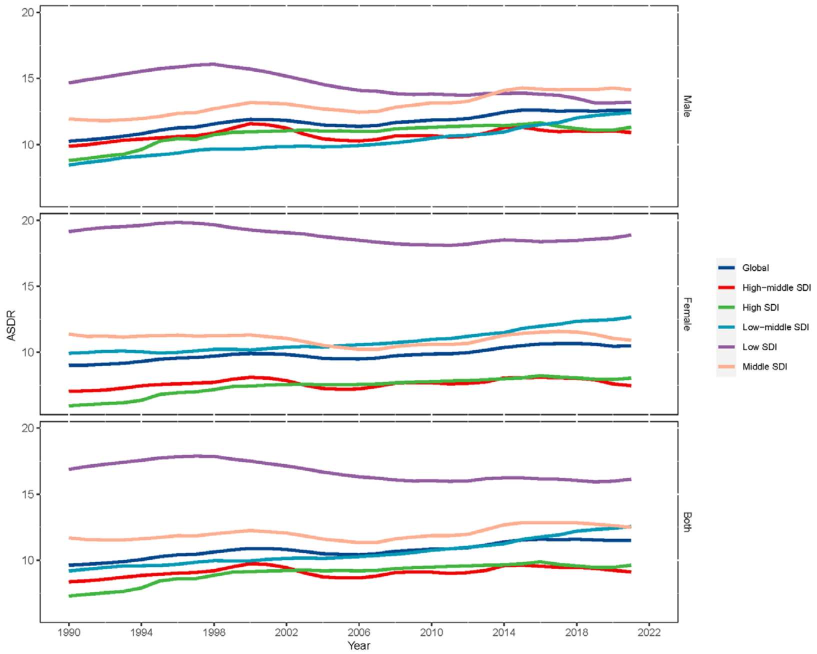

Figure S2. The ASDR of NRLC at the global and regional levels from 1990 through 2021

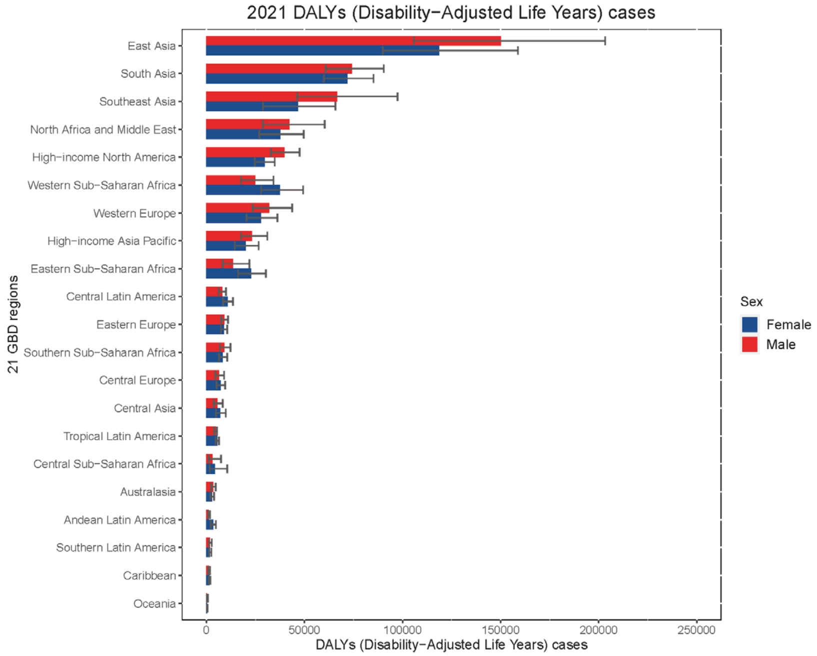

Figure S3. The DALYs cases of NRLC in 2021 for 21 GBD regions, by sex

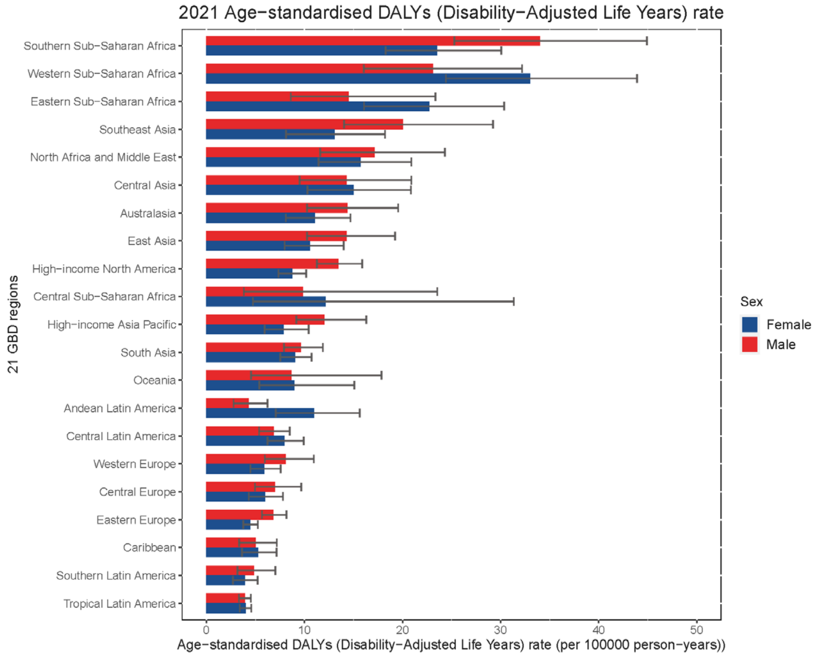

Figure S4. The ASDR of NRLC in 2021 for 21 GBD regions, by sex

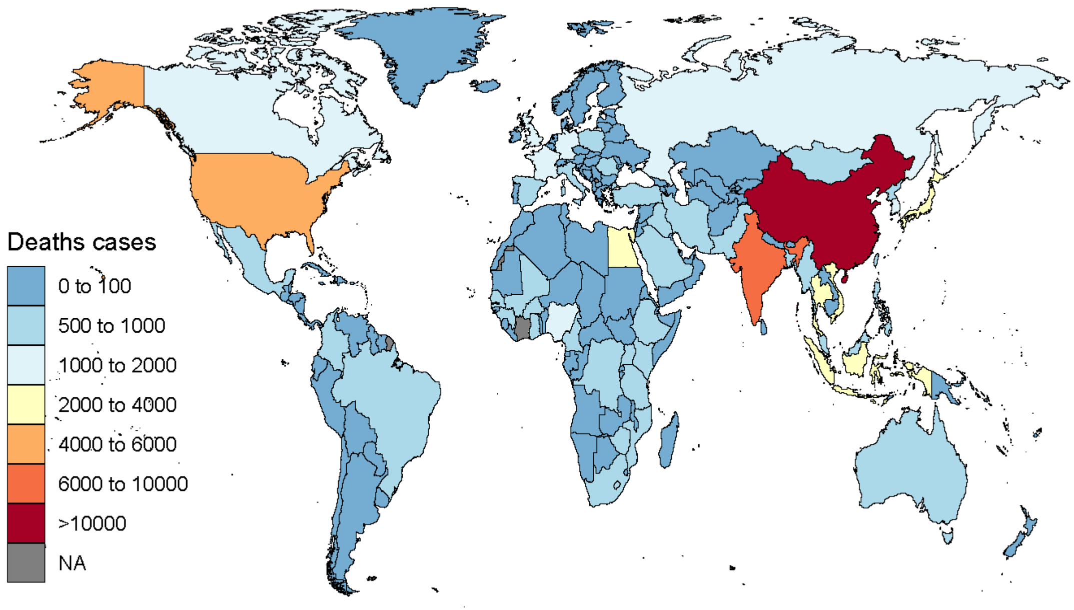

Figure S5. The deaths cases of NRLC in 2021 for 204 countries and territory

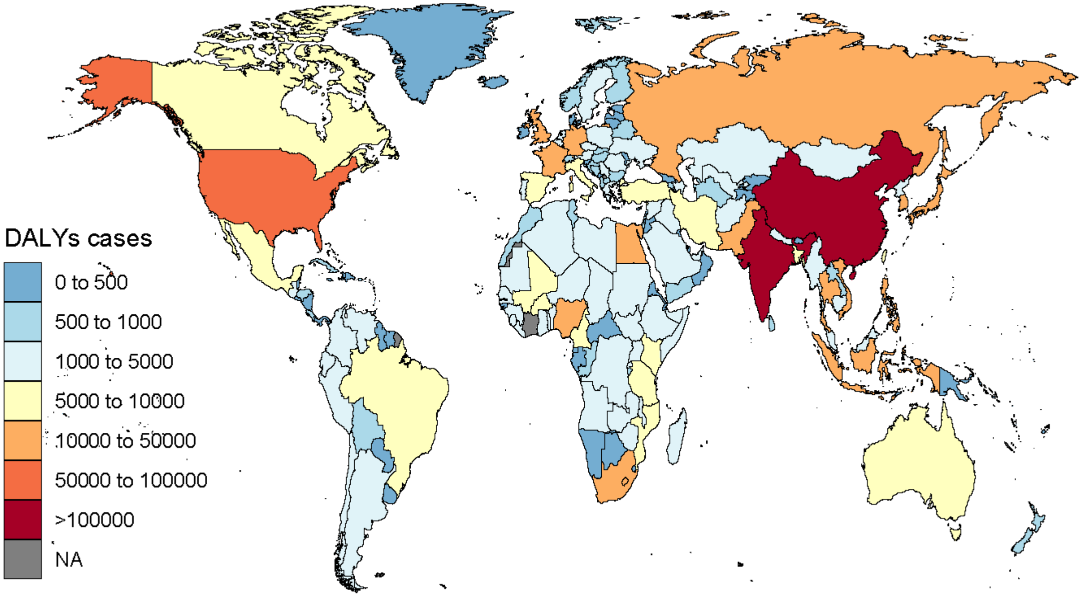

Figure S6. The DALYs cases of NRLC in 2021 for 204 countries and territory

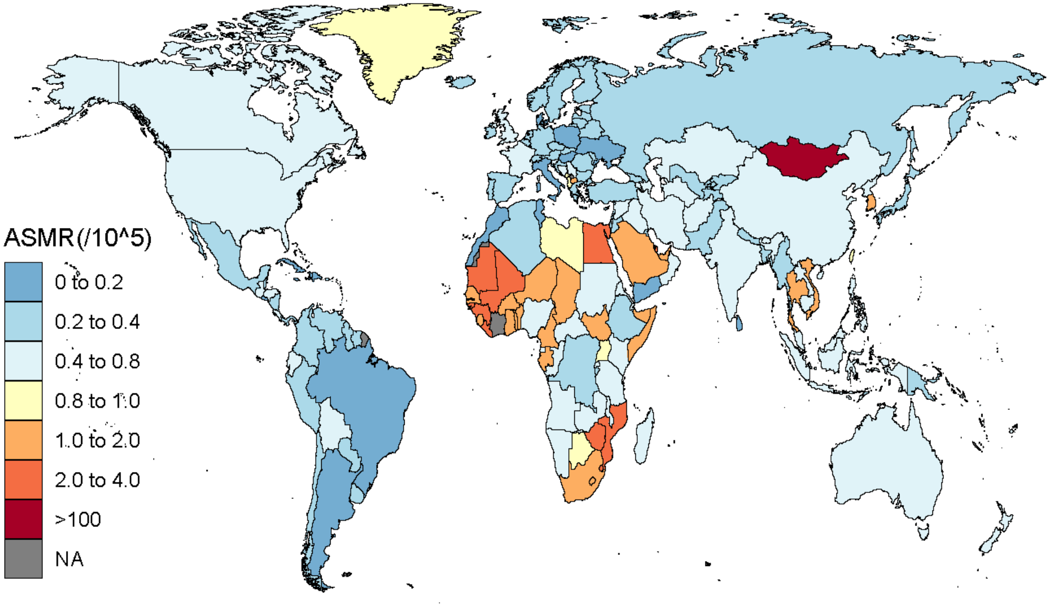

Figure S7. The ASMR of NRLC in 2021 for 204 countries and territory

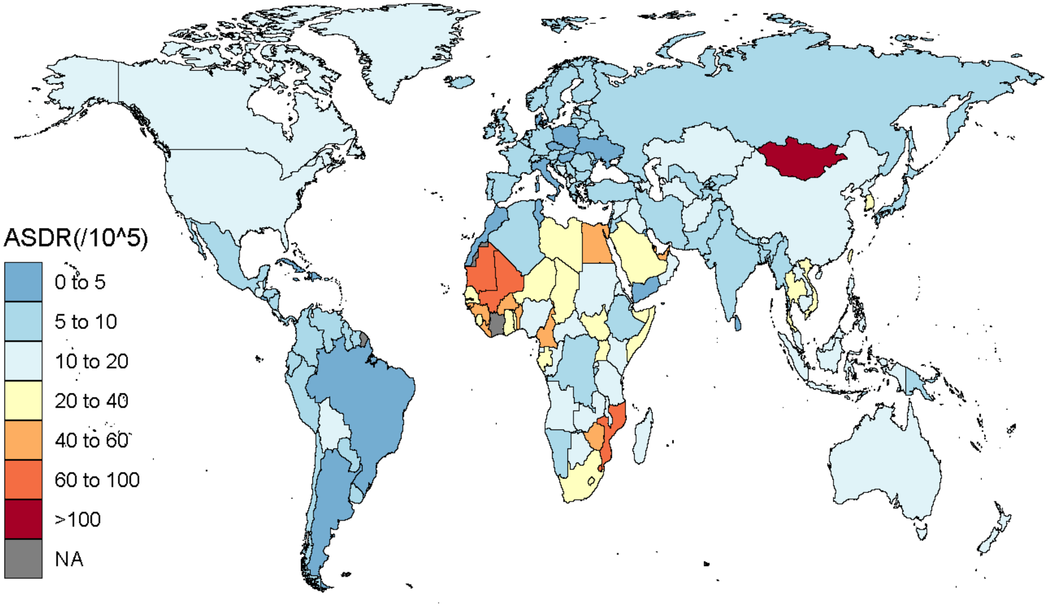

Figure S8. The ASDR of NRLC in 2021 for 204 countries and territory

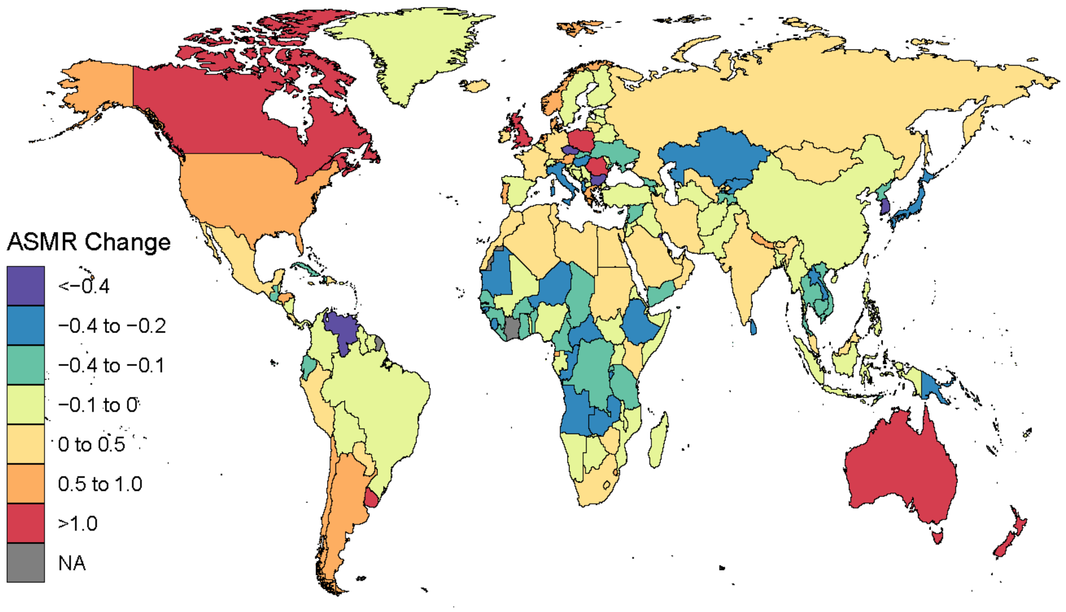

Figure S9. The change of ASMR from 1990 to 2021 in 204 countries and territory

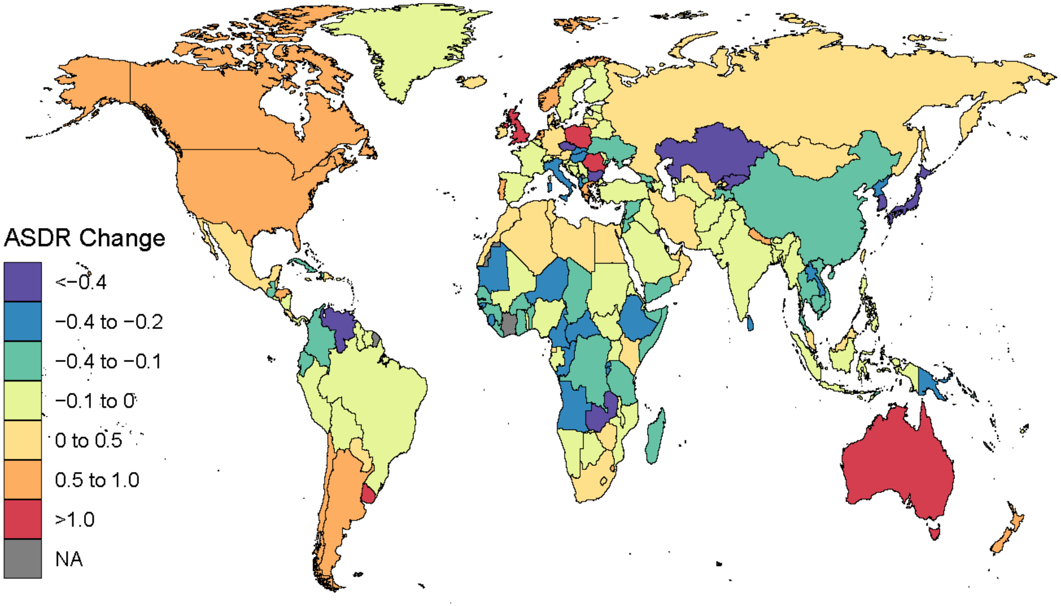

Figure S10. The change of ASDR from 1990 to 2021 in 204 countries and territory

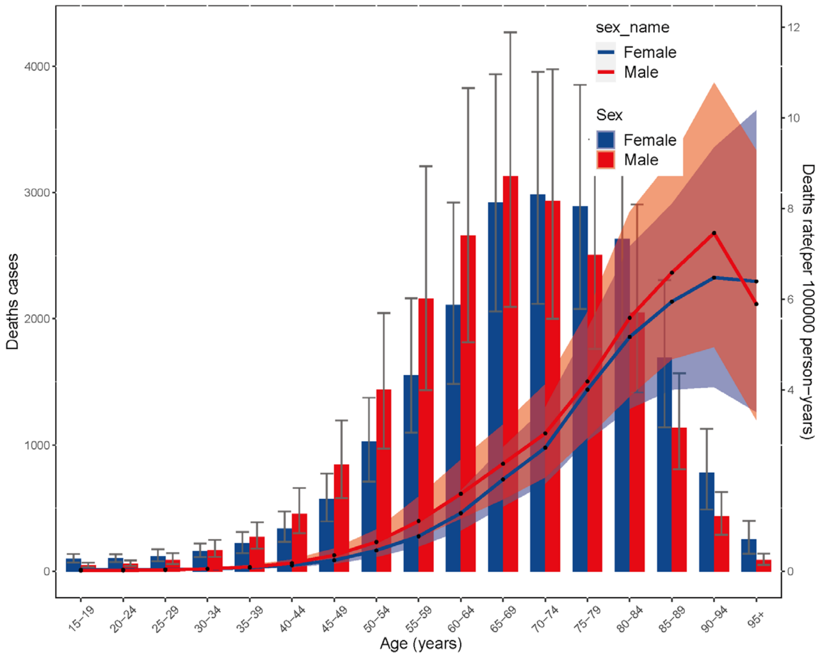

Figure S11. The effect of age and sex to deaths cases number and rate in 2021

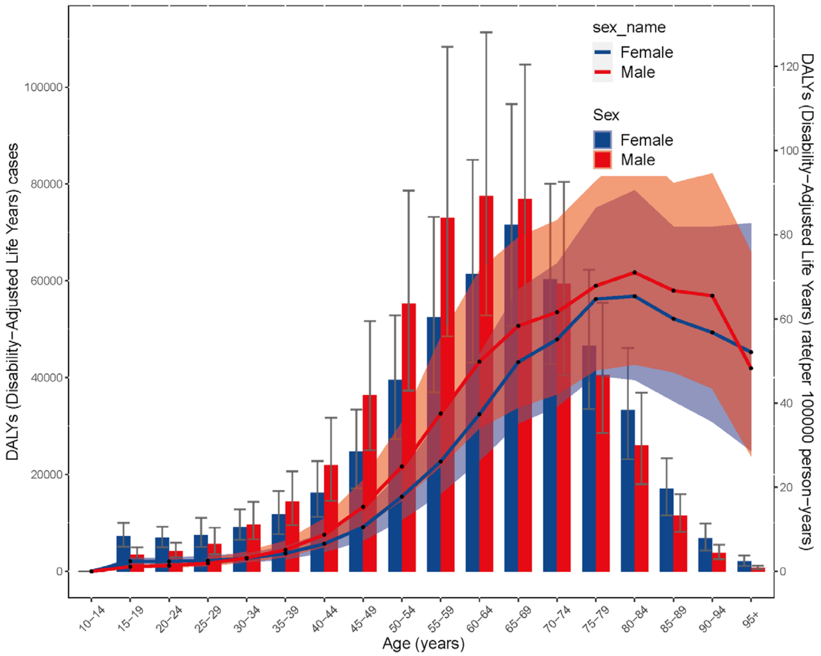

Figure S12. The effect of age and sex to DALYs cases number and rate in 2021

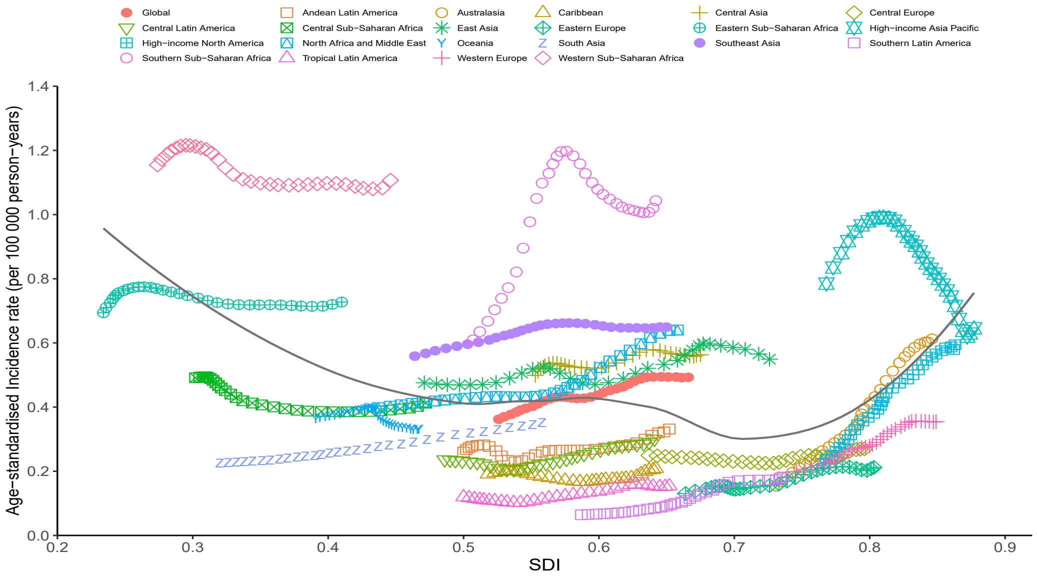

Figure S13. The association between SDI and ASIR from 1990 to 2021 in 21 GBD regions

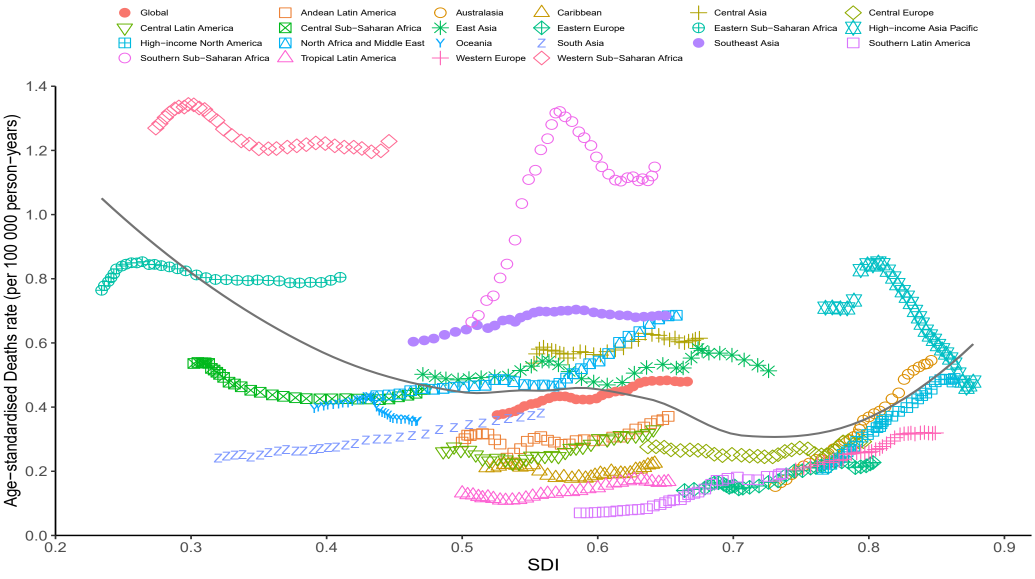

Figure S14. The association between SDI and ASMR from 1990 to 2021 in 21 GBD regions

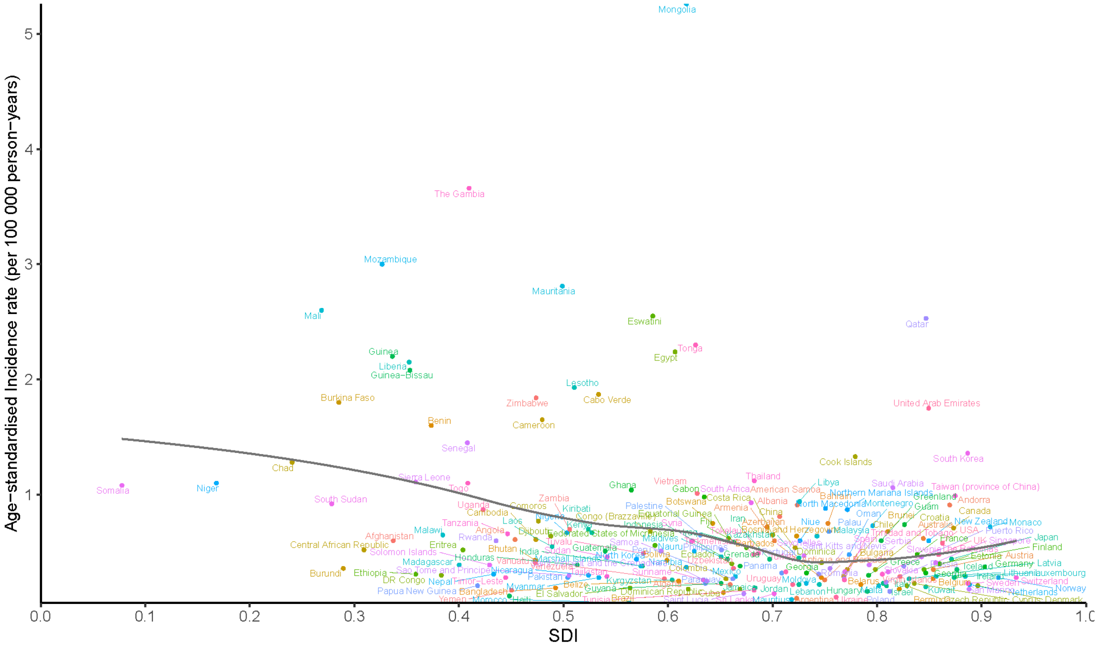

Figure S15. The association between SDI and ASIR in 2021 for 204 countries and territory

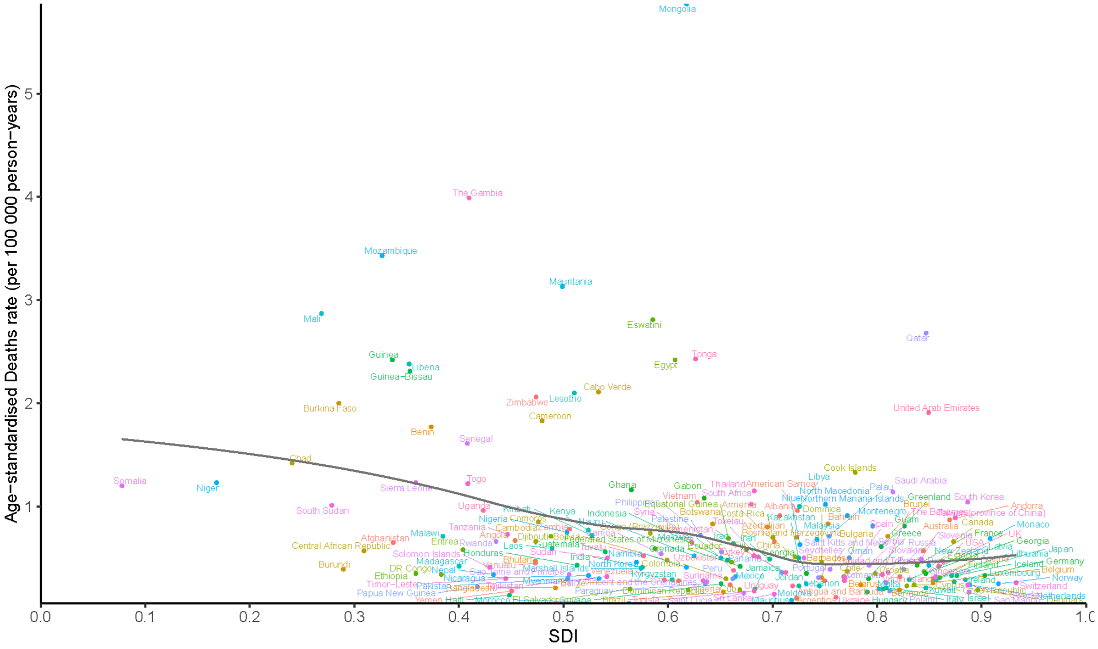

Figure S16. The association between SDI and ASMR in 2021 for 204 countries and territory
