# Supplementary material for: Burden of NASH related liver cancer from 1990 to 2021 at the global, regional, and national levels
Source: Front Nutr. 2025 Jan 27;12:1510563. doi: 10.3389/fnut.2025.1510563 (PMC11807830; doi:10.3389/fnut.2025.1510563)
Supplement: Supplementary file 2 [file Data_Sheet_2.pdf]

Additional file 2

Global Burden of NASH related liver cancer from 1990 to 2021 at the global, regional, and national levels

Table S1. The incidence of NRLC between 1990 and 2021 at national level, both sexes………………. 2-10

Table S2. The deaths of NRLC between 1990 and 2021 at national level, both sexes……………………10-18

Table S3. The DALYs of NRLC between 1990 and 2021 at national level, both sexes…………………..19-27

Table S1. The incidence of NRLC between 1990 and 2021 at national level, both sexes						
region	Case in 1990	Case in 2021	Change in absolute number (95% UI)	ASR in 1990	ASR in 2021	change in ASR per 100 000 population (95% UI)
China	4057(3237 to 4978)	11293(8663 to 14314)	178.36(111.36 to 257.8)	0.48(0.38 to 0.58)	0.54(0.42 to 0.68)	14.01(-12.27 to 46.47)
Democratic People's Republic of Korea	88(45 to 157)	110(69 to 171)	66.11(2.74 to 194.98)	0.53(0.28 to 0.97)	5.26(3.28 to 8.13)	-17.12(-48.75 to 47.38)
Taiwan (Province of China)	75(54 to 103)	4(3 to 6)	458.93(337.4 to 593.01)	0.45(0.32 to 0.62)	0.54(0.34 to 0.82)	121.05(78.77 to 165.98)
Cambodia	31(14 to 60)	20(13 to 29)	133.97(50.85 to 284.09)	0.69(0.31 to 1.38)	0.48(0.31 to 0.72)	-11.96(-42.54 to 46.48)
Indonesia	305(189 to 449)	134(59 to 248)	246.26(148.3 to 345.89)	0.3(0.19 to 0.44)	0.28(0.12 to 0.52)	48.76(5.69 to 92.09)
Lao People's Democratic Republic	15(9 to 24)	0(0 to 0)	67.54(7.44 to 155.4)	0.72(0.44 to 1.17)	0.38(0.21 to 0.62)	-24(-49.5 to 14.51)
Malaysia	34(22 to 50)	420(288 to 586)	417.81(258.68 to 631.92)	0.38(0.25 to 0.57)	0.99(0.69 to 1.38)	67.72(16.97 to 138.72)
Maldives	0(0 to 1)	26(17 to 38)	229.58(119.67 to 393.84)	0.51(0.3 to 0.8)	0.6(0.4 to 0.86)	-9.77(-39.05 to 32.25)
Myanmar	60(22 to 111)	72(36 to 131)	121.96(44.9 to 255.5)	0.26(0.1 to 0.47)	0.61(0.3 to 1.15)	7.68(-29.18 to 66.71)
Philippines	145(98 to 206)	39(22 to 62)	193.68(94.56 to 368.3)	0.48(0.32 to 0.69)	0.14(0.08 to 0.23)	9.41(-29.2 to 74.85)
Sri Lanka	18(12 to 25)	11(5 to 26)	117.7(36.88 to 233.02)	0.17(0.12 to 0.25)	0.21(0.09 to 0.51)	-18.07(-48.48 to 24.47)
Thailand	412(265 to 612)	2(1 to 4)	195.07(93.8 to 332.6)	1.18(0.75 to 1.75)	0.28(0.15 to 0.45)	-4.55(-37.05 to 40.38)
Timor-Leste	1(0 to 1)	1(1 to 2)	194.41(96.49 to 345.21)	0.3(0.18 to 0.47)	0.39(0.21 to 0.68)	-6.94(-36.35 to 42.42)
Viet Nam	394(244 to 620)	25(14 to 39)	158.34(61.72 to 309.99)	0.97(0.6 to 1.52)	0.55(0.31 to 0.86)	3.86(-33.17 to 60.82)
Fiji	1(1 to 2)	41(25 to 62)	207.49(75.24 to 414.75)	0.37(0.22 to 0.61)	0.64(0.4 to 0.96)	46.61(-15.52 to 140.81)
Kiribati	0(0 to 0)	12(8 to 18)	108.51(30.51 to 221.92)	0.63(0.41 to 0.92)	0.26(0.17 to 0.4)	7.36(-29.76 to 60.13)
Marshall Islands	0(0 to 0)	19(13 to 28)	192.35(75.13 to 397.02)	0.28(0.14 to 0.53)	0.32(0.22 to 0.46)	35.11(-16.67 to 119.71)
Micronesia (Federated States of)	0(0 to 0)	1(1 to 2)	93.11(20.39 to 210.68)	0.43(0.27 to 0.67)	0.46(0.28 to 0.7)	28.41(-19.67 to 101.39)
Papua New Guinea	5(2 to 12)	16(9 to 27)	114.14(41.32 to 262.18)	0.28(0.12 to 0.69)	0.29(0.16 to 0.49)	-26.04(-49.99 to 22.73)
Samoa	0(0 to 1)	1(0 to 1)	73.32(14.86 to 161.14)	0.5(0.31 to 0.78)	0.41(0.23 to 0.72)	1.06(-31.68 to 49.32)
Solomon Islands	1(0 to 1)	427(340 to 538)	138.37(33.17 to 499.55)	0.43(0.15 to 1.02)	0.52(0.42 to 0.65)	-9.13(-48.08 to 119.03)

Tonga	1(1 to 2)	0(0 to 1)	74.91(2.92 to 190.25)	1.9(1.07 to 3.18)	0.56(0.33 to 0.88)	21.26(-28.82 to 103.26)
Vanuatu	0(0 to 0)	146(86 to 236)	211.2(105.6 to 425.41)	0.37(0.2 to 0.78)	0.44(0.26 to 0.71)	10.48(-25.52 to 80.92)
Armenia	16(10 to 23)	1215(780 to 1801)	65.32(18.92 to 122.81)	0.61(0.4 to 0.9)	1.12(0.72 to 1.65)	-2.86(-29.49 to 30.37)
Azerbaijan	24(12 to 42)	1(0 to 1)	198.84(36.6 to 561.7)	0.49(0.24 to 0.87)	0.67(0.41 to 0.94)	48.38(-32.89 to 225.64)
Georgia	22(15 to 32)	96(60 to 144)	-13.66(-29.1 to 5.14)	0.35(0.24 to 0.51)	0.36(0.22 to 0.54)	-8.3(-23.85 to 11.65)
Kazakhstan	87(60 to 123)	48(31 to 72)	-11.54(-31.55 to 16.67)	0.68(0.47 to 0.97)	0.21(0.14 to 0.32)	-33.61(-49.3 to -12.73)
Kyrgyzstan	12(8 to 18)	1057(567 to 1644)	-2.7(-27.51 to 28.11)	0.41(0.28 to 0.59)	0.44(0.23 to 0.69)	-36.7(-52.99 to -16.54)
Mongolia	32(20 to 51)	36(21 to 58)	240.15(133.05 to 368.79)	3.03(1.85 to 4.76)	0.81(0.48 to 1.31)	73.72(17.12 to 147.26)
Tajikistan	9(5 to 15)	77(51 to 110)	86.12(0.79 to 241.42)	0.32(0.17 to 0.57)	0.45(0.29 to 0.63)	-7.58(-49.56 to 70.9)
Turkmenistan	7(5 to 9)	32(18 to 49)	200.98(112.19 to 317.12)	0.34(0.24 to 0.48)	0.33(0.2 to 0.51)	43.08(1.28 to 95.22)
Uzbekistan	26(16 to 40)	1017(635 to 1537)	271.68(145.79 to 473.66)	0.22(0.14 to 0.35)	1.01(0.65 to 1.51)	62.04(9.28 to 147.51)
Albania	21(13 to 32)	1(0 to 1)	68.63(-2.34 to 181.12)	1.11(0.68 to 1.67)	0.51(0.32 to 0.74)	-26.91(-57.51 to 21)
Bosnia and Herzegovina	22(14 to 31)	70(36 to 131)	87.61(33.85 to 163.04)	0.56(0.36 to 0.79)	0.72(0.36 to 1.34)	14.25(-18.16 to 57.52)
Bulgaria	78(51 to 115)	26(16 to 40)	-47.37(-63.82 to -22.94)	0.67(0.44 to 0.98)	0.88(0.54 to 1.33)	-56.65(-69.72 to -37.19)
Croatia	16(11 to 23)	177(116 to 259)	106.17(49.15 to 188.28)	0.28(0.19 to 0.41)	0.64(0.43 to 0.95)	23.07(-10.64 to 69.7)
Czechia	48(33 to 70)	86(60 to 115)	-0.47(-23.41 to 29.26)	0.35(0.24 to 0.5)	0.11(0.08 to 0.15)	-38.41(-52.52 to -19.81)
Hungary	35(24 to 51)	54(35 to 78)	-8.39(-33.52 to 21.02)	0.24(0.16 to 0.35)	0.4(0.26 to 0.57)	-32.94(-50.79 to -12.42)
North Macedonia	14(9 to 21)	2(1 to 3)	78.96(28.73 to 149.06)	0.81(0.52 to 1.2)	2.3(1.39 to 3.43)	8.94(-22.03 to 52.93)
Montenegro	3(2 to 5)	10(6 to 14)	118.93(43.36 to 224.11)	0.52(0.33 to 0.8)	0.35(0.23 to 0.5)	39.97(-7.99 to 108.47)
Poland	25(21 to 29)	49(40 to 58)	364.22(316.96 to 418.77)	0.06(0.05 to 0.07)	0.6(0.5 to 0.7)	172.22(142.41 to 203.94)
Romania	27(18 to 38)	41(25 to 63)	270.14(184.62 to 377.22)	0.1(0.07 to 0.14)	0.29(0.19 to 0.44)	167.74(109.45 to 237.74)
Serbia	39(24 to 60)	117(97 to 138)	70.92(17.45 to 141.19)	0.37(0.22 to 0.55)	0.16(0.14 to 0.19)	7.77(-22.91 to 54.18)
Slovakia	24(15 to 35)	337(272 to 407)	31.53(-10.15 to 93.82)	0.4(0.26 to 0.59)	0.23(0.19 to 0.28)	-17.59(-43.79 to 20.87)
Slovenia	8(6 to 12)	1834(1387 to 2272)	157.13(95.42 to 236.01)	0.34(0.24 to 0.47)	0.44(0.35 to 0.53)	36.99(5.8 to 76.47)
Belarus	23(16 to 33)	733(489 to 1066)	52.75(14.52 to 100.54)	0.18(0.12 to 0.25)	0.37(0.26 to 0.52)	25.28(-6.16 to 63.93)

Estonia	5(3 to 7)	17(11 to 23)	95.58(50.46 to 149.77)	0.24(0.16 to 0.34)	0.29(0.2 to 0.41)	45.88(13.42 to 84.02)
Latvia	7(5 to 10)	64(41 to 91)	48.45(15.27 to 91.14)	0.2(0.13 to 0.29)	0.27(0.17 to 0.38)	32.7(3.88 to 64.7)
Lithuania	8(5 to 11)	2(1 to 3)	114.61(73.31 to 160.37)	0.17(0.12 to 0.24)	0.21(0.13 to 0.3)	68.31(36.1 to 106.15)
Republic of Moldova	8(6 to 11)	1(1 to 2)	70(43.6 to 100.34)	0.18(0.13 to 0.25)	0.91(0.53 to 1.45)	23.84(3.92 to 44.23)
Russian Federation	214(182 to 247)	35(23 to 52)	163.31(139.38 to 187.86)	0.12(0.1 to 0.14)	0.41(0.27 to 0.62)	99.93(82.85 to 117.27)
Ukraine	99(78 to 122)	564(478 to 657)	-12.76(-37.98 to 18.76)	0.14(0.11 to 0.17)	0.24(0.2 to 0.28)	-17.69(-41.52 to 11.01)
Brunei Darussalam	1(0 to 1)	22(14 to 32)	177.8(90.7 to 311.55)	0.82(0.47 to 1.36)	0.46(0.3 to 0.69)	-17.51(-43.39 to 20.63)
Japan	1010(863 to 1171)	35(22 to 51)	81.52(49.39 to 113.62)	0.59(0.5 to 0.68)	0.22(0.14 to 0.32)	-25.36(-34.54 to -17.04)
Republic of Korea	583(373 to 873)	27(17 to 38)	122.34(59.07 to 213.52)	2.09(1.35 to 3.16)	0.21(0.14 to 0.31)	-34.79(-51.48 to -9.95)
Singapore	8(5 to 12)	273(185 to 381)	332.08(259.09 to 415.71)	0.38(0.25 to 0.57)	0.62(0.42 to 0.85)	8.23(-9.5 to 28.19)
Australia	28(20 to 39)	33(21 to 48)	865.86(713.3 to 1048.8)	0.14(0.1 to 0.2)	0.35(0.22 to 0.5)	326.88(260.3 to 406.08)
New Zealand	8(6 to 9)	26(18 to 37)	533.7(460.85 to 614.67)	0.2(0.17 to 0.23)	0.21(0.14 to 0.29)	201.19(166.79 to 239.15)
Andorra	0(0 to 1)	11(7 to 15)	216.18(94.26 to 415.77)	0.78(0.45 to 1.27)	0.27(0.18 to 0.36)	17.18(-27.17 to 88.45)
Austria	18(12 to 26)	66(40 to 102)	253.02(198.63 to 315.2)	0.15(0.1 to 0.21)	0.39(0.24 to 0.6)	132.69(98.39 to 172.72)
Belgium	27(17 to 40)	3(2 to 5)	133.33(98.74 to 172.02)	0.17(0.11 to 0.25)	0.29(0.19 to 0.42)	56.74(33.66 to 82.93)
Cyprus	2(1 to 2)	64(43 to 92)	207.08(113.98 to 344.92)	0.21(0.13 to 0.32)	0.35(0.24 to 0.5)	8.97(-21.86 to 54.2)
Denmark	9(6 to 12)	675(431 to 958)	209.17(161.84 to 265.31)	0.11(0.07 to 0.15)	0.48(0.32 to 0.68)	103.61(74.56 to 140.39)
Finland	17(11 to 25)	1297(834 to 1978)	210.29(161.57 to 271.73)	0.24(0.15 to 0.34)	1.36(0.88 to 2.06)	70.07(46.27 to 100.01)
France	224(151 to 326)	27(22 to 32)	200.71(154.09 to 254.64)	0.27(0.18 to 0.39)	0.28(0.23 to 0.32)	78.56(51.8 to 108.27)
Germany	241(163 to 343)	79(52 to 109)	203.51(156.08 to 261.81)	0.18(0.13 to 0.26)	0.22(0.15 to 0.3)	101.42(72.48 to 137.27)
Greece	21(14 to 30)	13(9 to 19)	254.42(204 to 307.82)	0.14(0.09 to 0.2)	0.23(0.16 to 0.32)	113.39(84.86 to 144.37)
Iceland	1(0 to 1)	5(3 to 8)	292.76(225.25 to 374.8)	0.18(0.12 to 0.26)	0.23(0.14 to 0.37)	91.67(58.49 to 131.28)
Ireland	5(4 to 8)	22(15 to 32)	313.59(240.29 to 393.66)	0.13(0.09 to 0.19)	0.28(0.18 to 0.39)	113.21(77.32 to 154.49)
Israel	9(6 to 13)	58(39 to 82)	197.86(150.34 to 254.17)	0.18(0.12 to 0.25)	0.1(0.07 to 0.14)	18.53(0.1 to 39.22)
Italy	263(218 to 313)	322(209 to 467)	28.04(16.15 to 40.74)	0.29(0.24 to 0.34)	0.33(0.22 to 0.49)	-20.11(-25.9 to -13.91)

Luxembourg	1(1 to 2)	18(11 to 27)	179.55(140.77 to 224.45)	0.2(0.14 to 0.29)	0.18(0.11 to 0.27)	43.95(25.48 to 64.94)
Malta	1(0 to 1)	2(1 to 2)	270.38(210.22 to 350.32)	0.13(0.09 to 0.19)	0.26(0.16 to 0.39)	59.13(34.34 to 93.29)
Netherlands	20(13 to 27)	1(1 to 2)	302.66(248.11 to 366.02)	0.1(0.07 to 0.13)	0.31(0.21 to 0.45)	126.1(96.33 to 161.91)
Norway	7(6 to 9)	1(1 to 1)	272.08(248.38 to 296.25)	0.11(0.09 to 0.12)	0.25(0.17 to 0.35)	157.92(141.11 to 173.23)
Portugal	16(11 to 23)	12(8 to 17)	353.72(287.97 to 432.28)	0.12(0.08 to 0.17)	0.22(0.14 to 0.31)	158.2(122.72 to 208.4)
Spain	108(71 to 155)	2(1 to 3)	198.28(149.42 to 254.36)	0.2(0.13 to 0.28)	0.35(0.23 to 0.5)	69.86(44.18 to 99.59)
Sweden	29(24 to 34)	12(7 to 17)	68.3(45.28 to 91.3)	0.19(0.16 to 0.22)	0.19(0.12 to 0.27)	19.28(3.22 to 36.5)
Switzerland	24(16 to 34)	1(1 to 2)	117.07(80.71 to 155.31)	0.23(0.16 to 0.32)	0.23(0.15 to 0.33)	22.6(3.54 to 43.85)
United Kingdom	125(103 to 149)	522(367 to 720)	407.23(376.98 to 434.84)	0.14(0.12 to 0.16)	0.71(0.51 to 0.97)	250.1(234.02 to 264.66)
Argentina	13(9 to 19)	32(20 to 47)	338.38(268.49 to 418.88)	0.04(0.03 to 0.06)	0.16(0.1 to 0.24)	151(111.69 to 196.7)
Chile	14(9 to 19)	65(45 to 92)	565.69(429.82 to 714.86)	0.14(0.09 to 0.2)	0.4(0.28 to 0.57)	152.31(102.17 to 208.13)
Uruguay	3(2 to 4)	52(34 to 76)	311.1(231.23 to 395.53)	0.07(0.05 to 0.11)	0.28(0.18 to 0.41)	198.58(140.72 to 262.09)
Canada	85(60 to 114)	6(4 to 8)	518.05(426.51 to 614.69)	0.26(0.19 to 0.35)	0.19(0.12 to 0.27)	175.12(136.37 to 215.18)
United States of America	717(612 to 830)	24(14 to 38)	371.57(351.32 to 391.57)	0.22(0.19 to 0.26)	0.39(0.22 to 0.62)	160.96(150.85 to 170.85)
Antigua and Barbuda	0(0 to 0)	0(0 to 0)	99.84(70.87 to 131.07)	0.24(0.16 to 0.34)	0.41(0.25 to 0.61)	5.53(-10.51 to 22.22)
Bahamas	0(0 to 1)	0(0 to 0)	166.62(103.91 to 237.27)	0.3(0.21 to 0.41)	0.25(0.17 to 0.35)	3.58(-19.26 to 31.71)
Barbados	1(0 to 1)	0(0 to 1)	122.14(75.45 to 186.87)	0.22(0.15 to 0.3)	0.38(0.26 to 0.54)	29.21(1.66 to 66.88)
Belize	0(0 to 0)	1(1 to 2)	374.08(265.87 to 478.62)	0.17(0.12 to 0.24)	0.28(0.18 to 0.42)	49.82(19.08 to 81.29)
Cuba	21(15 to 30)	5(3 to 7)	68.03(38.28 to 102.56)	0.21(0.14 to 0.29)	0.26(0.17 to 0.38)	-11.94(-27.78 to 5.7)
Dominica	0(0 to 0)	7(4 to 10)	132.05(41.19 to 255.84)	0.24(0.14 to 0.39)	0.73(0.46 to 1.08)	71.54(6.94 to 161.29)
Dominican Republic	4(3 to 6)	35(24 to 50)	354.3(196.15 to 562.73)	0.11(0.07 to 0.16)	0.65(0.44 to 0.92)	65.91(8.08 to 141.5)
Grenada	0(0 to 0)	100(67 to 148)	199.99(124.28 to 285.37)	0.18(0.12 to 0.26)	0.27(0.18 to 0.39)	107.65(55.02 to 170.13)
Guyana	1(1 to 1)	0(0 to 0)	84.79(33.41 to 151.34)	0.21(0.14 to 0.3)	0.26(0.18 to 0.36)	8.31(-21.25 to 45.53)
Haiti	4(2 to 9)	0(0 to 0)	97.35(24.2 to 197.2)	0.14(0.07 to 0.28)	0.14(0.1 to 0.21)	-10.27(-41.65 to 33.85)
Jamaica	2(1 to 3)	8(4 to 17)	199.42(112.01 to 293.38)	0.1(0.07 to 0.15)	0.12(0.06 to 0.26)	77.24(27.24 to 132.2)

Saint Lucia	0(0 to 0)	15(9 to 21)	119.6(67.61 to 184.09)	0.18(0.12 to 0.26)	0.3(0.2 to 0.45)	-21.47(-39.43 to 0.65)
Saint Vincent and the Grenadines	0(0 to 0)	13(8 to 20)	79.12(43.3 to 119.11)	0.27(0.19 to 0.38)	0.23(0.14 to 0.35)	-8.53(-25.78 to 11.35)
Suriname	0(0 to 1)	90(55 to 133)	258.32(138.07 to 414.86)	0.18(0.12 to 0.27)	0.27(0.17 to 0.41)	40.82(-6.43 to 102.12)
Trinidad and Tobago	2(1 to 3)	49(39 to 60)	164.15(94.17 to 246.94)	0.23(0.16 to 0.32)	0.23(0.18 to 0.28)	12.03(-17.32 to 45.92)
Bolivia (Plurinational State of)	10(6 to 16)	55(38 to 78)	277.52(140.21 to 464.63)	0.32(0.2 to 0.52)	0.51(0.34 to 0.73)	32.94(-14.85 to 97.84)
Ecuador	22(16 to 30)	131(80 to 191)	194.57(126 to 288.78)	0.42(0.3 to 0.58)	0.59(0.36 to 0.87)	-5(-26.48 to 24.74)
Peru	20(13 to 30)	36(25 to 49)	353.99(189.44 to 593.38)	0.17(0.11 to 0.26)	0.18(0.13 to 0.25)	59.94(1.86 to 146.24)
Colombia	42(30 to 59)	2(1 to 3)	247.61(180.99 to 329.98)	0.25(0.17 to 0.35)	0.68(0.42 to 1.03)	7.46(-12.47 to 30.62)
Costa Rica	7(5 to 10)	360(300 to 424)	410.03(313.57 to 510.13)	0.4(0.27 to 0.55)	0.29(0.24 to 0.34)	62.76(33.16 to 94.31)
El Salvador	5(3 to 7)	147(98 to 210)	137.29(78.19 to 222.17)	0.16(0.11 to 0.24)	0.27(0.18 to 0.38)	12.96(-16.74 to 53.7)
Guatemala	19(13 to 26)	75(51 to 109)	194.08(145.75 to 255.09)	0.57(0.4 to 0.79)	0.3(0.21 to 0.42)	-10.34(-24.26 to 7.14)
Honduras	4(2 to 7)	5(3 to 7)	572.1(332.94 to 969.45)	0.17(0.09 to 0.35)	0.2(0.13 to 0.29)	122.2(40.14 to 259.35)
Mexico	65(56 to 76)	636(522 to 756)	451.09(388.82 to 514.62)	0.16(0.13 to 0.18)	0.48(0.4 to 0.57)	85.37(65.52 to 107.26)
Nicaragua	4(2 to 6)	3381(2855 to 3958)	296.19(192.34 to 433.95)	0.24(0.15 to 0.38)	0.58(0.49 to 0.68)	25.75(-7.79 to 69.71)
Panama	4(3 to 6)	77(50 to 116)	226.98(161.19 to 299.78)	0.3(0.2 to 0.43)	0.27(0.17 to 0.4)	7.77(-14.15 to 32.68)
Venezuela (Bolivarian Republic of)	43(29 to 59)	172(113 to 256)	81.08(37.21 to 136.39)	0.45(0.3 to 0.63)	1.06(0.68 to 1.53)	-41.32(-55.58 to -23.25)
Brazil	105(91 to 120)	22(13 to 36)	261.82(239.49 to 284.36)	0.12(0.1 to 0.14)	0.17(0.1 to 0.27)	27.28(20.78 to 34.28)
Paraguay	3(2 to 4)	5(3 to 8)	364.82(210.27 to 580.01)	0.13(0.09 to 0.19)	0.75(0.48 to 1.12)	78(19.52 to 159.46)
Algeria	14(9 to 22)	379(320 to 440)	431.78(244.39 to 721.59)	0.12(0.08 to 0.18)	0.15(0.13 to 0.18)	88.61(23.86 to 189.54)
Bahrain	1(1 to 2)	48(30 to 71)	296.9(182.54 to 467.36)	0.89(0.56 to 1.31)	0.94(0.59 to 1.41)	-16.1(-38.1 to 15.77)
Egypt	298(167 to 553)	73(17 to 192)	366(152.16 to 713.25)	1.15(0.62 to 2.21)	0.61(0.15 to 1.61)	94.98(3.67 to 242.36)
Iran (Islamic Republic of)	55(43 to 73)	262(161 to 399)	467.82(347.45 to 580.04)	0.25(0.19 to 0.33)	0.19(0.12 to 0.29)	77.9(40.97 to 113.19)
Iraq	31(20 to 47)	15(10 to 22)	318.81(174.99 to 500.41)	0.39(0.24 to 0.61)	0.65(0.42 to 0.98)	48.49(-4.11 to 112.94)
Jordan	3(2 to 4)	313(257 to 371)	473.29(251.82 to 838.63)	0.21(0.12 to 0.36)	0.44(0.36 to 0.52)	3.57(-37.73 to 73.9)
Kuwait	3(2 to 4)	4458(3741 to 5340)	87.64(41.37 to 138.2)	0.45(0.3 to 0.63)	0.38(0.32 to 0.45)	-55.94(-66.25 to -44.65)

Lebanon	5(3 to 8)	17(11 to 26)	170.17(86.32 to 279.95)	0.24(0.15 to 0.36)	2.53(1.57 to 3.96)	-5.58(-34.88 to 34.63)
Libya	10(6 to 15)	74(49 to 108)	379.15(179.12 to 711.82)	0.53(0.31 to 0.86)	0.31(0.21 to 0.44)	75.1(2.73 to 191.08)
Morocco	5(3 to 7)	14(9 to 21)	326.71(173.27 to 559.07)	0.03(0.02 to 0.05)	0.32(0.21 to 0.48)	78.47(13.3 to 176.91)
Palestine	6(3 to 9)	91(59 to 130)	171.54(85.13 to 312.31)	0.67(0.41 to 1.04)	0.35(0.23 to 0.5)	-3.11(-33.85 to 47.47)
Oman	2(1 to 3)	15(9 to 23)	486.12(241.94 to 980.92)	0.24(0.13 to 0.43)	0.22(0.13 to 0.33)	100.11(10.21 to 263.26)
Qatar	1(1 to 2)	60(37 to 95)	1317.21(835.98 to 1925.6)	1.36(0.86 to 2)	0.6(0.35 to 1.01)	85.18(27.71 to 155.59)
Saudi Arabia	38(20 to 62)	37(20 to 60)	352.62(140.5 to 720.17)	0.67(0.34 to 1.12)	0.43(0.24 to 0.7)	57.85(-14.67 to 173.89)
Syrian Arab Republic	30(18 to 50)	77(49 to 113)	151.29(43.15 to 327.89)	0.59(0.34 to 1)	0.22(0.14 to 0.33)	1.18(-43.22 to 74.8)
Tunisia	5(3 to 8)	9(6 to 14)	331.86(139.2 to 591.87)	0.1(0.06 to 0.16)	0.48(0.3 to 0.71)	61.41(-10.81 to 160.66)
Turkey	78(51 to 120)	48(31 to 69)	283.9(168.82 to 454.99)	0.24(0.15 to 0.37)	0.65(0.4 to 0.94)	37.95(-3.52 to 103.1)
United Arab Emirates	4(3 to 6)	301(191 to 438)	1368.22(870.07 to 2135.95)	1.02(0.59 to 1.62)	0.33(0.21 to 0.47)	72.05(16.54 to 157.39)
Yemen	9(3 to 20)	1390(884 to 2005)	144.64(61.17 to 335.16)	0.2(0.07 to 0.44)	2.24(1.41 to 3.28)	-14.6(-43.43 to 54.31)
Afghanistan	33(18 to 57)	3(1 to 4)	82.45(19.95 to 179.35)	0.48(0.26 to 0.87)	0.43(0.24 to 0.69)	24.05(-15.11 to 80.28)
Bangladesh	74(48 to 112)	11(5 to 25)	254.52(146.41 to 424.43)	0.15(0.1 to 0.23)	0.52(0.2 to 1.18)	25.03(-12.56 to 82.73)
Bhutan	1(0 to 1)	4(2 to 6)	277.75(126 to 543.71)	0.28(0.15 to 0.45)	0.64(0.39 to 1.04)	55.09(-5.96 to 155.21)
India	1117(894 to 1337)	282(142 to 523)	299.26(238.88 to 366.99)	0.24(0.19 to 0.29)	3(1.52 to 5.68)	58.56(34.83 to 85.4)
Nepal	13(8 to 20)	108(47 to 257)	448.06(240.77 to 725.99)	0.14(0.09 to 0.22)	0.3(0.12 to 0.78)	123.02(40.4 to 236.44)
Pakistan	113(86 to 145)	1(0 to 1)	220.27(129.05 to 345.43)	0.19(0.15 to 0.25)	0.47(0.3 to 0.68)	42.98(0.27 to 95.86)
Angola	32(5 to 94)	166(80 to 318)	129.87(48.84 to 336.75)	0.8(0.12 to 2.34)	1.8(0.87 to 3.48)	-23.58(-50.94 to 48.96)
Central African Republic	9(3 to 19)	125(72 to 188)	29.68(-15.24 to 87.93)	0.77(0.28 to 1.73)	2.2(1.24 to 3.28)	-33.23(-54.85 to -5.1)
Congo	10(4 to 23)	44(28 to 67)	76.78(15.35 to 178.71)	0.99(0.38 to 2.22)	2.15(1.33 to 3.26)	-31.47(-52.14 to 4.36)
Democratic Republic of the Congo	55(26 to 112)	10(6 to 17)	97.18(22.93 to 196.6)	0.35(0.16 to 0.77)	0.98(0.55 to 1.64)	-13.72(-47.3 to 30.28)
Equatorial Guinea	1(0 to 1)	8(5 to 12)	458.49(206.48 to 919.23)	0.3(0.15 to 0.62)	1.87(1.1 to 2.86)	105.58(13.38 to 260.66)
Gabon	4(2 to 9)	4(2 to 6)	136.73(31.38 to 325.51)	0.75(0.3 to 1.66)	0.77(0.44 to 1.17)	31.36(-26.31 to 135.25)
Burundi	12(7 to 21)	50(25 to 81)	41.43(-8.75 to 134.56)	0.52(0.3 to 0.88)	0.46(0.24 to 0.74)	-32.14(-55.55 to 7.77)

Comoros	2(1 to 2)	2(1 to 2)	137.74(51.87 to 263.06)	0.82(0.48 to 1.28)	0.09(0.06 to 0.12)	-5.75(-38.76 to 41.22)
Djibouti	1(0 to 1)	119(68 to 181)	510.76(290.37 to 881.92)	0.47(0.28 to 0.79)	1.84(1.07 to 2.83)	36.96(-12.03 to 111.27)
Eritrea	5(3 to 9)	37(22 to 55)	164.48(78.78 to 301.04)	0.5(0.29 to 0.86)	0.6(0.36 to 0.92)	4.27(-28.5 to 50.93)
Ethiopia	80(59 to 111)	81(52 to 123)	70.69(16.75 to 161.65)	0.41(0.3 to 0.58)	1.6(1.02 to 2.43)	-23.52(-46.56 to 13.24)
Kenya	32(22 to 49)	62(31 to 117)	411.17(279.75 to 611.6)	0.38(0.26 to 0.58)	1.08(0.53 to 2.09)	87.38(36.8 to 161.69)
Madagascar	20(13 to 31)	203(103 to 324)	113.7(41.32 to 213.65)	0.39(0.24 to 0.6)	1.65(0.82 to 2.65)	-0.42(-32.87 to 51.42)
Malawi	17(11 to 26)	41(25 to 67)	181.15(85.71 to 328.96)	0.44(0.27 to 0.66)	1.11(0.67 to 1.73)	46.61(-1.86 to 120.79)
Mauritius	3(2 to 4)	60(36 to 95)	-44.06(-50.63 to -37.04)	0.43(0.29 to 0.59)	1.75(1.05 to 2.7)	-79.12(-81.52 to -76.71)
Mozambique	111(67 to 171)	18(8 to 40)	153.89(19.08 to 459.2)	2.13(1.28 to 3.24)	0.68(0.28 to 1.52)	41.12(-35.68 to 198.24)
Rwanda	21(13 to 32)	18(11 to 29)	73.21(11.76 to 162.27)	0.74(0.45 to 1.11)	0.36(0.21 to 0.58)	-18.9(-45.3 to 18.27)
Seychelles	0(0 to 1)	13(8 to 20)	19.49(-10.91 to 66.4)	0.8(0.54 to 1.14)	0.22(0.14 to 0.34)	-41.28(-55.48 to -19.58)
Somalia	25(12 to 46)	363(269 to 489)	142.38(60.23 to 285.88)	1.08(0.5 to 2)	0.28(0.2 to 0.37)	0.05(-36.03 to 54.67)
United Republic of Tanzania	76(50 to 118)	3(2 to 5)	121.79(55.96 to 220.5)	0.7(0.45 to 1.07)	0.62(0.35 to 1.04)	-4.99(-32.44 to 35.79)
Uganda	52(32 to 77)	75(46 to 112)	152.62(51.41 to 304.97)	0.81(0.5 to 1.21)	0.6(0.37 to 0.89)	8.51(-34.92 to 73.35)
Zambia	32(19 to 54)	23(12 to 43)	41.82(-29.26 to 152.13)	1.09(0.64 to 1.83)	0.17(0.08 to 0.33)	-36.2(-65.69 to 8.27)
Botswana	3(1 to 6)	72(41 to 116)	248.97(68.43 to 678.15)	0.6(0.27 to 1.16)	0.31(0.18 to 0.49)	26.38(-39.69 to 182.97)
Lesotho	5(2 to 11)	20(12 to 30)	284.59(22.77 to 1185.03)	0.65(0.24 to 1.39)	0.06(0.04 to 0.09)	198.61(0.83 to 849.08)
Namibia	2(1 to 3)	136(89 to 218)	210.01(76.07 to 435.57)	0.26(0.13 to 0.43)	0.31(0.21 to 0.5)	42.82(-20.91 to 147.13)
South Africa	108(66 to 173)	420(338 to 508)	289.91(131.39 to 566.73)	0.5(0.3 to 0.81)	0.93(0.76 to 1.12)	87.18(8.51 to 225.6)
Eswatini	3(1 to 6)	170(107 to 259)	347.16(37.65 to 1329.75)	1.15(0.5 to 2.06)	0.66(0.4 to 1)	122.6(-30.49 to 577.56)
Zimbabwe	48(28 to 75)	43(25 to 71)	149.59(37.6 to 318.14)	1.22(0.7 to 1.9)	0.39(0.22 to 0.62)	50.67(-14.29 to 148.94)
Benin	38(19 to 68)	14(8 to 23)	114.5(29.38 to 288.34)	1.89(0.91 to 3.44)	0.52(0.3 to 0.86)	-15.62(-49.99 to 53.46)
Burkina Faso	89(38 to 194)	164(92 to 256)	86.72(27.38 to 193)	2.1(0.9 to 4.58)	1.04(0.57 to 1.62)	-14.07(-41.97 to 34.48)
Cameroon	90(54 to 135)	131(82 to 200)	126.83(32.66 to 280.15)	2.06(1.2 to 3.13)	0.87(0.53 to 1.34)	-19.64(-52.55 to 35.38)
Cabo Verde	3(2 to 5)	11(5 to 22)	188.01(30.99 to 408.07)	1.16(0.69 to 1.97)	0.75(0.39 to 1.45)	61.4(-27.84 to 184.34)

Chad	37(15 to 79)	46(19 to 109)	95.21(29.73 to 218.87)	1.31(0.53 to 2.8)	0.7(0.3 to 1.6)	-2.52(-33.53 to 57.22)
C 么 te d'Ivoire	23(14 to 36)	14(6 to 31)	112.96(2.14 to 288.9)	0.6(0.35 to 0.93)	2.55(1.11 to 5.43)	-23.43(-63.88 to 39.25)
Gambia	9(6 to 13)	15(9 to 23)	314.19(135.28 to 598.62)	2.49(1.59 to 3.74)	2.08(1.32 to 3.15)	47.02(-16.99 to 145.38)
Ghana	65(37 to 115)	20(9 to 44)	151.62(12.21 to 432.67)	1.08(0.58 to 1.94)	1.93(0.83 to 4.1)	-3.6(-58.28 to 102.19)
Guinea	77(49 to 114)	58(32 to 92)	62.29(-1.25 to 167.38)	2.33(1.5 to 3.43)	2.81(1.51 to 4.44)	-5.7(-42.1 to 53.32)
Guinea-Bissau	11(5 to 18)	108(69 to 162)	35(-9.34 to 162.37)	2.71(1.17 to 4.67)	1.45(0.9 to 2.17)	-23.22(-48.76 to 52.1)
Liberia	25(13 to 42)	163(119 to 219)	78.43(8.12 to 182.03)	2.21(1.16 to 3.74)	0.7(0.52 to 0.94)	-2.61(-40.36 to 56.03)
Mali	86(55 to 127)	36(21 to 57)	160.7(84.06 to 270.77)	2.19(1.43 to 3.29)	3.66(2.03 to 5.89)	18.67(-15.8 to 68.4)
Mauritania	38(11 to 85)	0(0 to 0)	52.44(-19.59 to 278.74)	3.85(1.07 to 8.47)	0.18(0.12 to 0.27)	-26.95(-62.53 to 79.51)
Niger	46(21 to 91)	0(0 to 1)	90.03(27.68 to 223.91)	1.67(0.77 to 3.33)	1.33(0.86 to 1.95)	-33.85(-54.95 to 14.05)
Nigeria	268(126 to 532)	1(1 to 2)	114.34(27.01 to 304.01)	0.63(0.3 to 1.25)	0.6(0.42 to 0.86)	6.92(-36.88 to 101.77)
Sao Tome and Principe	0(0 to 0)	0(0 to 0)	86.34(-4.11 to 234.83)	0.27(0.18 to 0.4)	0.49(0.27 to 0.83)	10.05(-43.5 to 98.55)
Senegal	48(32 to 72)	5(3 to 7)	122.18(38.62 to 250.41)	1.52(1 to 2.26)	0.37(0.24 to 0.55)	-4.76(-41.27 to 52.41)
Sierra Leone	32(12 to 67)	223(144 to 347)	28.59(-29.37 to 207.77)	1.58(0.57 to 3.3)	2.6(1.65 to 4.07)	-30.1(-61 to 67.14)
Togo	12(8 to 17)	0(0 to 1)	235.62(96.57 to 422.65)	0.95(0.61 to 1.44)	0.3(0.16 to 0.49)	15.41(-31.76 to 83.6)
American Samoa	0(0 to 0)	575(379 to 853)	275.51(156.62 to 432.7)	0.51(0.33 to 0.76)	0.67(0.46 to 0.97)	76.68(21.56 to 148.39)
Bermuda	0(0 to 0)	72(39 to 136)	38.87(12.64 to 72.42)	0.3(0.21 to 0.41)	1.28(0.67 to 2.43)	-39.06(-50.37 to -25.29)
Cook Islands	0(0 to 0)	39(24 to 61)	160.22(74.45 to 274.55)	1.07(0.7 to 1.59)	1.1(0.64 to 1.74)	24.04(-16.4 to 78.3)
Greenland	0(0 to 0)	0(0 to 1)	146.56(67.85 to 250.13)	0.6(0.4 to 0.9)	0.91(0.6 to 1.32)	22.16(-17.1 to 72.14)
Guam	0(0 to 0)	0(0 to 1)	435.67(328.36 to 566.74)	0.31(0.21 to 0.45)	0.74(0.45 to 1.11)	92.86(51.36 to 141.66)
Monaco	0(0 to 0)	87(49 to 155)	225.91(103.22 to 438.52)	0.3(0.18 to 0.49)	1.1(0.61 to 1.93)	139.95(57.38 to 281.67)
Nauru	0(0 to 0)	27(18 to 38)	22.94(-23.62 to 95.16)	0.58(0.36 to 0.86)	0.38(0.26 to 0.52)	-12.15(-45.14 to 42.12)
Niue	0(0 to 0)	87(50 to 136)	44.2(-16.34 to 133.35)	0.4(0.24 to 0.65)	0.46(0.26 to 0.72)	47.26(-12.61 to 136.16)
Northern Mariana Islands	0(0 to 0)	0(0 to 1)	307.45(193.01 to 468.43)	0.62(0.4 to 0.94)	0.87(0.56 to 1.26)	41.66(4.1 to 86.19)
Palau	0(0 to 0)	1(0 to 1)	215.37(83.51 to 446.81)	0.52(0.31 to 0.9)	0.72(0.44 to 1.09)	30.96(-20.88 to 117.89)

Puerto Rico	10(7 to 15)	0(0 to 0)	153.9(100.05 to 217.9)	0.29(0.2 to 0.4)	0.68(0.41 to 1.07)	30.8(4.11 to 61.66)
Saint Kitts and Nevis	0(0 to 0)	0(0 to 0)	70.16(31.59 to 118.53)	0.35(0.24 to 0.5)	0.36(0.24 to 0.51)	1.01(-19.48 to 23.68)
San Marino	0(0 to 0)	0(0 to 0)	173.1(73.45 to 324.59)	0.14(0.08 to 0.21)	0.49(0.29 to 0.79)	32.92(-15.76 to 108.74)
Tokelau	0(0 to 0)	1(0 to 1)	51.13(-1.83 to 134.43)	0.36(0.19 to 0.69)	0.29(0.18 to 0.46)	35.28(-11.05 to 110.42)
Tuvalu	0(0 to 0)	0(0 to 0)	95.32(40.81 to 180.82)	0.4(0.23 to 0.72)	0.51(0.31 to 0.8)	22.23(-13.12 to 76.83)
United States Virgin Islands	0(0 to 0)	35(20 to 55)	259.77(111.39 to 483.99)	0.19(0.12 to 0.31)	0.92(0.53 to 1.48)	50.94(-13.54 to 138.04)
South Sudan	16(9 to 26)	0(0 to 0)	118.42(48.19 to 223.82)	0.63(0.38 to 1.01)	0.18(0.1 to 0.3)	47.68(0.38 to 115.62)
Sudan	27(13 to 54)	0(0 to 0)	216.47(70.34 to 461.29)	0.3(0.14 to 0.61)	0.6(0.36 to 0.94)	52.93(-19.23 to 170.64)

Table S2. The deaths of NRLC between 1990 and 2021 at national level, both sexes

region	Case in 1990	Case in 2021	Change in absolute number (95% UI)	ASR in 1990	ASR in 2021	change in ASR per 100 000 population (95% UI)
China	4128(3293 to 5068)	10409(8036 to 13180)	152.15(91.18 to 220.92)	0.5(0.4 to 0.61)	0.51(0.39 to 0.64)	0.83(-22.16 to 28.53)
Democratic People's Republic of Korea	90(47 to 164)	149(86 to 241)	64.74(2.18 to 190.36)	0.56(0.3 to 1.04)	0.46(0.26 to 0.72)	-19.37(-49.55 to 41.55)
Taiwan (Province of China)	74(53 to 101)	378(257 to 531)	411.22(296.45 to 542.31)	0.45(0.32 to 0.63)	0.89(0.61 to 1.25)	95.32(58.47 to 136.39)
Cambodia	32(15 to 64)	75(37 to 137)	134.34(51.32 to 282.75)	0.75(0.33 to 1.55)	0.66(0.33 to 1.28)	-11.53(-42.53 to 47.19)

Indonesia	314(195 to 463)	1085(576 to 1699)	245.64(147.33 to 342.74)	0.32(0.2 to 0.47)	0.48(0.25 to 0.75)	49.64(6.3 to 94.53)
Lao People's Democratic Republic	16(9 to 25)	26(15 to 41)	67.35(8.72 to 158.37)	0.78(0.48 to 1.26)	0.59(0.33 to 0.94)	-23.83(-49.7 to 15.12)
Malaysia	36(24 to 53)	182(121 to 269)	401.55(248.35 to 611.27)	0.41(0.27 to 0.62)	0.68(0.45 to 1.01)	63.32(13.51 to 133.45)
Maldives	0(0 to 1)	1(1 to 2)	229.16(119.21 to 395.15)	0.56(0.33 to 0.9)	0.5(0.3 to 0.76)	-11.55(-40.37 to 31.74)
Myanmar	63(23 to 116)	140(62 to 261)	122.5(44.31 to 253.84)	0.28(0.11 to 0.51)	0.3(0.13 to 0.56)	7.23(-29.36 to 64.91)
Philippines	150(101 to 213)	444(353 to 559)	195.1(94.18 to 372.89)	0.52(0.34 to 0.75)	0.56(0.45 to 0.7)	8.43(-30.69 to 74.02)
Sri Lanka	19(13 to 27)	39(22 to 64)	108.6(30.2 to 215.11)	0.19(0.13 to 0.28)	0.15(0.08 to 0.24)	-23.24(-51.97 to 15.35)
Thailand	430(277 to 641)	1243(803 to 1863)	188.72(88.62 to 325.75)	1.27(0.81 to 1.9)	1.15(0.74 to 1.71)	-9.84(-40.5 to 31.71)
Timor-Leste	1(0 to 1)	2(1 to 4)	201.83(102.39 to 362.78)	0.32(0.2 to 0.51)	0.3(0.16 to 0.5)	-7.45(-36.54 to 41.71)
Viet Nam	416(256 to 652)	1020(646 to 1527)	145.01(53.74 to 286.53)	1.04(0.64 to 1.62)	1.04(0.67 to 1.57)	-0.37(-36.21 to 54.12)
Fiji	1(1 to 2)	4(3 to 7)	208.33(76.12 to 416.4)	0.4(0.23 to 0.67)	0.58(0.36 to 0.88)	46.09(-15.6 to 142.51)
Kiribati	0(0 to 0)	1(0 to 1)	107.43(31.4 to 216.17)	0.67(0.44 to 0.99)	0.72(0.44 to 1.02)	7.83(-29.48 to 60.75)
Marshall Islands	0(0 to 0)	0(0 to 0)	184.47(71.98 to 378.33)	0.3(0.15 to 0.57)	0.4(0.22 to 0.67)	34.11(-17.34 to 116.84)
Micronesia (Federated States of)	0(0 to 0)	0(0 to 1)	89.01(17.03 to 196.46)	0.46(0.29 to 0.71)	0.59(0.34 to 0.93)	28.54(-19.22 to 101.47)
Papua New Guinea	5(2 to 13)	11(5 to 27)	112.85(38.81 to 264.34)	0.3(0.13 to 0.77)	0.22(0.09 to 0.55)	-26.65(-51.15 to 24.73)
Samoa	0(0 to 1)	1(0 to 1)	70.74(13.48 to 156.54)	0.55(0.34 to 0.85)	0.54(0.34 to 0.79)	-0.51(-32.42 to 46.86)
Solomon Islands	1(0 to 1)	1(1 to 3)	134.81(30.95 to 487.31)	0.45(0.16 to 1.1)	0.41(0.23 to 0.72)	-10.54(-48.93 to 115.44)
Tonga	1(1 to 2)	2(1 to 3)	75.27(2.42 to 186.03)	2.03(1.14 to 3.38)	2.43(1.47 to 3.6)	19.66(-30.52 to 99.65)
Vanuatu	0(0 to 0)	1(0 to 1)	210.9(106 to 420.81)	0.41(0.21 to 0.89)	0.45(0.25 to 0.77)	9.7(-26.6 to 79.99)
Armenia	17(11 to 25)	29(19 to 42)	67.77(20.02 to 125.71)	0.68(0.44 to 0.99)	0.66(0.43 to 0.96)	-3.53(-30.46 to 29.49)
Azerbaijan	25(13 to 45)	75(38 to 137)	196.3(36.43 to 548.22)	0.54(0.27 to 0.97)	0.8(0.39 to 1.46)	47.77(-32.74 to 227.66)
Georgia	24(16 to 34)	21(14 to 31)	-11.12(-27.22 to 8.21)	0.38(0.25 to 0.55)	0.35(0.23 to 0.51)	-7.54(-22.89 to 11.35)
Kazakhstan	91(64 to 130)	82(54 to 117)	-10.29(-31.42 to 18.79)	0.73(0.51 to 1.05)	0.5(0.32 to 0.7)	-31.73(-47.88 to -9.95)
Kyrgyzstan	13(9 to 19)	13(8 to 19)	-3.04(-26.97 to 26.78)	0.45(0.3 to 0.64)	0.29(0.18 to 0.43)	-36(-52.03 to -15.52)
Mongolia	34(21 to 53)	118(73 to 183)	243.99(137.51 to 376.1)	3.26(2.01 to 5.13)	5.87(3.61 to 9.12)	79.88(22.93 to 156.7)

Tajikistan	9(5 to 17)	17(9 to 29)	82.21(-3.34 to 236.8)	0.35(0.18 to 0.65)	0.32(0.17 to 0.54)	-7.43(-50.81 to 74.62)
Turkmenistan	7(5 to 10)	20(13 to 31)	198.34(111.29 to 311.03)	0.36(0.25 to 0.51)	0.52(0.33 to 0.77)	41.99(1.97 to 93.65)
Uzbekistan	27(17 to 43)	99(62 to 150)	265.92(142.53 to 461.61)	0.24(0.15 to 0.37)	0.38(0.24 to 0.58)	61.67(9.66 to 146.33)
Albania	23(15 to 35)	40(23 to 65)	70.93(-1.45 to 187.09)	1.26(0.76 to 1.88)	0.91(0.53 to 1.48)	-27.67(-58.39 to 20.66)
Bosnia and Herzegovina	23(15 to 33)	45(28 to 68)	93.49(38.03 to 172.48)	0.62(0.4 to 0.87)	0.7(0.43 to 1.04)	14.03(-18.64 to 57.21)
Bulgaria	85(55 to 127)	46(28 to 70)	-46.09(-63.07 to -21.37)	0.76(0.49 to 1.12)	0.32(0.21 to 0.48)	-57.65(-70.79 to -38.21)
Croatia	17(12 to 24)	33(21 to 49)	94.31(41.24 to 172.19)	0.31(0.21 to 0.45)	0.35(0.22 to 0.5)	12.3(-18.42 to 55.54)
Czechia	53(36 to 77)	52(33 to 79)	-2.54(-25.01 to 27.2)	0.38(0.26 to 0.55)	0.23(0.15 to 0.34)	-40.5(-54.27 to -22.38)
Hungary	39(26 to 56)	35(23 to 52)	-9.07(-33.95 to 20.11)	0.27(0.18 to 0.38)	0.17(0.11 to 0.26)	-34.9(-51.98 to -15.22)
North Macedonia	16(10 to 23)	28(17 to 43)	79.65(28.78 to 151.78)	0.9(0.58 to 1.35)	1.02(0.62 to 1.53)	12.96(-19.75 to 59.82)
Montenegro	3(2 to 5)	8(5 to 11)	120.03(41.95 to 226.49)	0.57(0.36 to 0.88)	0.81(0.51 to 1.2)	41.03(-8.87 to 111.51)
Poland	29(24 to 33)	132(110 to 156)	362.7(315.15 to 416.57)	0.07(0.06 to 0.08)	0.18(0.15 to 0.21)	163.65(136.03 to 194.5)
Romania	29(20 to 43)	110(74 to 162)	274.4(188.8 to 382.93)	0.11(0.07 to 0.16)	0.29(0.2 to 0.42)	160.8(105.05 to 229.43)
Serbia	42(26 to 64)	72(44 to 112)	73.44(19.05 to 146.56)	0.41(0.25 to 0.62)	0.43(0.26 to 0.65)	4.71(-24.75 to 50.29)
Slovakia	26(17 to 39)	34(20 to 54)	30.87(-10.26 to 92.63)	0.44(0.28 to 0.64)	0.36(0.21 to 0.56)	-18.59(-44.41 to 18.38)
Slovenia	9(6 to 13)	23(15 to 34)	154.68(93.83 to 233.22)	0.37(0.25 to 0.51)	0.49(0.32 to 0.72)	32.97(2.13 to 71.85)
Belarus	25(17 to 35)	38(24 to 55)	51.33(13.2 to 100.96)	0.19(0.13 to 0.27)	0.24(0.15 to 0.34)	23.03(-8.21 to 61.76)
Estonia	5(4 to 8)	11(7 to 16)	98.87(53.59 to 153.33)	0.26(0.18 to 0.37)	0.37(0.25 to 0.54)	44.07(12.39 to 80.39)
Latvia	8(5 to 11)	12(8 to 17)	53.21(18.82 to 98.57)	0.22(0.14 to 0.32)	0.29(0.2 to 0.39)	32.68(3.56 to 64.69)
Lithuania	8(6 to 12)	17(12 to 24)	111.64(70.06 to 158.08)	0.18(0.12 to 0.26)	0.29(0.2 to 0.41)	61.18(30.46 to 96.13)
Republic of Moldova	8(6 to 12)	15(10 to 21)	73.87(46.94 to 104.66)	0.2(0.14 to 0.27)	0.25(0.17 to 0.35)	23.15(3.51 to 43.39)
Russian Federation	229(194 to 265)	614(520 to 717)	168.52(143.51 to 193.64)	0.13(0.11 to 0.15)	0.26(0.22 to 0.3)	100.33(82.9 to 117.84)
Ukraine	104(82 to 129)	90(63 to 119)	-13.22(-38.25 to 17.29)	0.14(0.12 to 0.18)	0.12(0.08 to 0.15)	-19.2(-42.44 to 8.47)
Brunei Darussalam	1(0 to 1)	2(1 to 3)	164.37(81.95 to 288.84)	0.88(0.51 to 1.45)	0.71(0.44 to 1.08)	-19.32(-44.53 to 17.57)
Japan	837(714 to 977)	1470(1105 to 1848)	75.56(43.88 to 106.59)	0.49(0.42 to 0.57)	0.33(0.26 to 0.4)	-33.07(-41.46 to -25.42)

Republic of Korea	590(371 to 883)	990(626 to 1485)	67.8(19.78 to 136.68)	2.21(1.45 to 3.35)	1.04(0.66 to 1.56)	-52.84(-64.6 to -35.46)
Singapore	8(5 to 12)	27(18 to 41)	242.6(184.97 to 302.92)	0.38(0.25 to 0.58)	0.32(0.21 to 0.49)	-15.63(-29.16 to -0.98)
Australia	29(20 to 41)	260(179 to 365)	791.84(652.13 to 970.44)	0.15(0.1 to 0.21)	0.57(0.39 to 0.79)	280.42(222.58 to 349.9)
New Zealand	7(6 to 8)	37(30 to 44)	451.59(385.93 to 523.67)	0.17(0.14 to 0.2)	0.43(0.36 to 0.52)	155.58(127.28 to 188.68)
Andorra	0(0 to 1)	1(1 to 2)	209.26(89.37 to 398.68)	0.79(0.46 to 1.3)	0.87(0.51 to 1.39)	10.04(-31.22 to 73.27)
Austria	18(12 to 27)	58(38 to 84)	213.28(165.65 to 270.39)	0.15(0.1 to 0.21)	0.3(0.2 to 0.43)	101.7(72.15 to 136.08)
Belgium	30(18 to 44)	66(41 to 95)	120.45(87.41 to 158.74)	0.18(0.12 to 0.27)	0.26(0.17 to 0.38)	43.39(22.64 to 68.15)
Cyprus	2(1 to 3)	5(3 to 8)	186.05(100.57 to 310.79)	0.24(0.15 to 0.37)	0.24(0.15 to 0.37)	-1.32(-28.58 to 39.69)
Denmark	7(5 to 11)	25(15 to 36)	233.17(184.33 to 291.8)	0.09(0.06 to 0.12)	0.19(0.12 to 0.28)	117.36(86.38 to 155.26)
Finland	16(10 to 23)	43(28 to 63)	173.66(131.86 to 228.02)	0.21(0.14 to 0.31)	0.31(0.2 to 0.44)	45.47(25.42 to 70.45)
France	238(158 to 345)	639(406 to 924)	168.28(126.41 to 220.4)	0.28(0.19 to 0.41)	0.43(0.28 to 0.61)	52.21(29.7 to 76.88)
Germany	256(171 to 366)	676(446 to 1019)	164.44(124.52 to 216)	0.19(0.13 to 0.27)	0.33(0.22 to 0.47)	69.67(44.16 to 100.62)
Greece	23(15 to 33)	81(55 to 117)	257.86(206.55 to 315.69)	0.15(0.1 to 0.22)	0.3(0.21 to 0.43)	101.92(75.16 to 129.24)
Iceland	1(0 to 1)	2(1 to 3)	270.37(205.84 to 347.43)	0.18(0.12 to 0.27)	0.33(0.22 to 0.47)	78.3(47.03 to 116)
Ireland	6(4 to 8)	22(14 to 32)	279.02(213.96 to 353.16)	0.14(0.09 to 0.2)	0.27(0.18 to 0.38)	92.17(59.76 to 129.14)
Israel	10(6 to 14)	27(18 to 39)	183.61(139.12 to 239.49)	0.19(0.13 to 0.27)	0.21(0.14 to 0.3)	10.55(-6.45 to 30.55)
Italy	265(218 to 315)	315(248 to 386)	18.82(6.62 to 31.45)	0.29(0.24 to 0.34)	0.2(0.17 to 0.24)	-30.63(-35.92 to -25.24)
Luxembourg	1(1 to 2)	3(2 to 5)	163.44(126.94 to 205.58)	0.21(0.15 to 0.31)	0.29(0.18 to 0.41)	33.34(16.24 to 52.97)
Malta	1(0 to 1)	2(1 to 3)	252.29(194.53 to 329.7)	0.14(0.09 to 0.2)	0.2(0.13 to 0.3)	46.04(23.38 to 76.55)
Netherlands	22(15 to 30)	87(58 to 126)	300.77(246.86 to 362.56)	0.11(0.07 to 0.15)	0.24(0.16 to 0.33)	122.03(92.48 to 158.63)
Norway	8(6 to 9)	26(21 to 30)	238.34(216.29 to 260.3)	0.11(0.09 to 0.13)	0.25(0.21 to 0.29)	131.19(116.21 to 145.44)
Portugal	18(12 to 26)	79(52 to 115)	342.21(276.41 to 421.91)	0.13(0.09 to 0.18)	0.31(0.21 to 0.45)	139.64(109.08 to 185.64)
Spain	114(74 to 167)	313(200 to 466)	173.83(128.44 to 228.74)	0.21(0.14 to 0.3)	0.31(0.2 to 0.45)	47.89(25.6 to 73.44)
Sweden	33(26 to 39)	53(42 to 65)	64.25(41.93 to 86.55)	0.21(0.17 to 0.25)	0.24(0.19 to 0.29)	13.68(-1.59 to 29.72)
Switzerland	24(16 to 35)	49(31 to 74)	106.12(70.74 to 145.37)	0.22(0.15 to 0.32)	0.26(0.17 to 0.37)	14.41(-3.87 to 34.69)

United Kingdom	125(102 to 149)	582(474 to 699)	366.75(337.17 to 393.83)	0.13(0.11 to 0.16)	0.42(0.35 to 0.5)	215.26(199.81 to 229.12)
Argentina	14(10 to 21)	63(42 to 89)	336.71(268.19 to 421.61)	0.05(0.03 to 0.06)	0.11(0.07 to 0.16)	145.62(107.92 to 189.49)
Chile	15(10 to 21)	96(62 to 137)	550.29(418.2 to 695.46)	0.15(0.1 to 0.22)	0.37(0.24 to 0.52)	142.52(94.33 to 197.39)
Uruguay	3(2 to 5)	13(8 to 18)	307.86(229.95 to 392.57)	0.08(0.05 to 0.11)	0.23(0.15 to 0.33)	190.56(134.76 to 253.06)
Canada	84(59 to 114)	502(348 to 700)	496.43(408.43 to 594.63)	0.26(0.18 to 0.34)	0.66(0.47 to 0.91)	157.5(121.74 to 195.03)
United States of America	660(561 to 771)	2799(2341 to 3270)	324.07(307.33 to 341.52)	0.2(0.17 to 0.23)	0.47(0.4 to 0.55)	132.49(124.4 to 140.63)
Antigua and Barbuda	0(0 to 0)	0(0 to 0)	93.28(65.35 to 123.15)	0.27(0.18 to 0.37)	0.28(0.19 to 0.4)	5.63(-10.11 to 22.15)
Bahamas	0(0 to 1)	1(1 to 2)	166.42(105.1 to 237.65)	0.32(0.22 to 0.44)	0.34(0.23 to 0.49)	3.76(-19.19 to 32.18)
Barbados	1(0 to 1)	2(1 to 2)	118.73(72.26 to 181.34)	0.24(0.17 to 0.33)	0.31(0.2 to 0.46)	28.7(0.87 to 65.24)
Belize	0(0 to 0)	1(1 to 1)	356.79(251.98 to 458.98)	0.19(0.13 to 0.26)	0.28(0.18 to 0.39)	47.41(16.79 to 78.42)
Cuba	23(16 to 33)	39(26 to 53)	65.64(35.02 to 99.92)	0.23(0.16 to 0.32)	0.19(0.13 to 0.26)	-14.92(-30.45 to 2.59)
Dominica	0(0 to 0)	0(0 to 1)	131.21(39.38 to 255.11)	0.27(0.16 to 0.44)	0.46(0.28 to 0.69)	72.91(6.87 to 164.6)
Dominican Republic	4(3 to 6)	19(12 to 29)	357.49(197.67 to 565.66)	0.12(0.08 to 0.18)	0.19(0.12 to 0.29)	62.89(6.14 to 138.4)
Grenada	0(0 to 0)	0(0 to 1)	190.17(116.54 to 275.09)	0.2(0.13 to 0.3)	0.42(0.29 to 0.6)	110.85(58.19 to 175.86)
Guyana	1(1 to 1)	2(1 to 2)	82.89(32.9 to 144.97)	0.23(0.15 to 0.33)	0.25(0.16 to 0.36)	7.71(-21.26 to 43.58)
Haiti	4(2 to 9)	9(4 to 18)	98.12(25.87 to 200.2)	0.15(0.08 to 0.31)	0.14(0.06 to 0.29)	-9.7(-40.46 to 35.86)
Jamaica	2(1 to 3)	6(4 to 9)	194.77(110.44 to 289.91)	0.12(0.08 to 0.17)	0.2(0.13 to 0.3)	74.8(25.21 to 129.81)
Saint Lucia	0(0 to 0)	0(0 to 1)	116.71(65.54 to 178.72)	0.21(0.14 to 0.29)	0.16(0.11 to 0.23)	-23.55(-41.32 to -2.12)
Saint Vincent and the Grenadines	0(0 to 0)	0(0 to 1)	76.6(41.97 to 115.93)	0.3(0.21 to 0.42)	0.27(0.18 to 0.38)	-9.7(-26.75 to 10.18)
Suriname	0(0 to 1)	2(1 to 3)	257.48(137.72 to 410.47)	0.2(0.13 to 0.3)	0.28(0.17 to 0.42)	39.37(-6.64 to 101.34)
Trinidad and Tobago	2(1 to 3)	5(3 to 8)	161.13(91.79 to 243.37)	0.26(0.17 to 0.36)	0.28(0.18 to 0.4)	8.59(-20.51 to 41.87)
Bolivia (Plurinational State of)	11(6 to 17)	40(23 to 66)	282.99(144.8 to 471.17)	0.36(0.22 to 0.59)	0.48(0.27 to 0.8)	34.21(-13.83 to 99.72)
Ecuador	24(17 to 33)	73(50 to 104)	200.66(132.39 to 293.06)	0.48(0.33 to 0.66)	0.46(0.32 to 0.65)	-3.98(-25.77 to 25.04)
Peru	22(14 to 33)	100(60 to 152)	357.08(191.88 to 598.39)	0.19(0.12 to 0.29)	0.3(0.18 to 0.46)	58.52(1.26 to 145.35)
Colombia	46(32 to 64)	163(107 to 233)	255.95(186.77 to 343.69)	0.28(0.19 to 0.39)	0.3(0.2 to 0.43)	6.99(-11.89 to 30.9)

Costa Rica	8(5 to 10)	38(25 to 54)	410.21(311.1 to 513.41)	0.44(0.3 to 0.61)	0.7(0.46 to 0.99)	60.68(31.39 to 93.04)
El Salvador	5(4 to 8)	13(8 to 18)	138.77(79.19 to 223.69)	0.18(0.12 to 0.26)	0.2(0.13 to 0.29)	11.4(-17.63 to 49.7)
Guatemala	20(14 to 27)	60(41 to 85)	199.82(150.54 to 261.41)	0.63(0.44 to 0.89)	0.56(0.37 to 0.8)	-10.61(-24.75 to 6.09)
Honduras	4(2 to 7)	26(15 to 40)	579.55(335.38 to 988.43)	0.19(0.1 to 0.39)	0.43(0.25 to 0.69)	123.3(39.07 to 265.86)
Mexico	70(60 to 82)	389(324 to 458)	453.38(392.69 to 516.09)	0.17(0.15 to 0.2)	0.32(0.27 to 0.38)	82.84(63.47 to 103.95)
Nicaragua	4(3 to 6)	16(10 to 23)	296.07(194.75 to 433.1)	0.27(0.17 to 0.42)	0.33(0.21 to 0.49)	24.99(-7.76 to 68.53)
Panama	5(3 to 7)	16(10 to 23)	227.3(159.41 to 300.46)	0.33(0.23 to 0.47)	0.36(0.23 to 0.52)	6.08(-16.11 to 30.82)
Venezuela (Bolivarian Republic of)	46(31 to 64)	83(52 to 124)	81.97(36.94 to 138.47)	0.5(0.33 to 0.7)	0.29(0.18 to 0.43)	-41.44(-56.01 to -23.43)
Brazil	111(97 to 128)	409(345 to 477)	267.07(244.49 to 289.87)	0.13(0.11 to 0.15)	0.17(0.14 to 0.19)	25.79(19.33 to 32.84)
Paraguay	3(2 to 5)	14(9 to 21)	361.73(209.57 to 573.77)	0.14(0.09 to 0.21)	0.25(0.16 to 0.37)	76.3(17.89 to 154)
Algeria	15(10 to 23)	81(51 to 118)	427.93(240.23 to 717.32)	0.13(0.08 to 0.2)	0.24(0.15 to 0.37)	86.97(21.84 to 186.75)
Bahrain	1(1 to 2)	5(3 to 8)	279.58(171.22 to 442.83)	1.01(0.63 to 1.47)	0.83(0.53 to 1.26)	-17.51(-39.37 to 14.96)
Egypt	311(174 to 581)	1427(889 to 2052)	358.63(147.7 to 698.79)	1.26(0.66 to 2.44)	2.42(1.5 to 3.54)	91.39(0.5 to 235.21)
Iran (Islamic Republic of)	59(45 to 78)	338(277 to 401)	476.19(353.72 to 589.89)	0.28(0.21 to 0.37)	0.49(0.4 to 0.58)	74.68(38.44 to 109.97)
Iraq	33(21 to 50)	136(82 to 199)	311.12(167.47 to 490.55)	0.43(0.25 to 0.65)	0.64(0.4 to 0.95)	49.79(-3.86 to 115.08)
Jordan	3(2 to 5)	16(9 to 23)	461.42(238.83 to 821.42)	0.23(0.13 to 0.39)	0.23(0.14 to 0.36)	2.17(-39.62 to 71.11)
Kuwait	3(2 to 4)	5(3 to 7)	86.9(39.89 to 137.76)	0.48(0.32 to 0.67)	0.21(0.14 to 0.3)	-56.02(-66.05 to -44.57)
Lebanon	5(3 to 8)	14(9 to 21)	167.35(83.43 to 277)	0.26(0.16 to 0.39)	0.23(0.15 to 0.35)	-10.04(-37.63 to 29.32)
Libya	11(6 to 16)	49(31 to 74)	368.24(175 to 683.63)	0.58(0.33 to 0.94)	1(0.64 to 1.52)	73.47(2.75 to 185.82)
Morocco	5(3 to 8)	21(13 to 31)	324.72(168.99 to 556.72)	0.04(0.02 to 0.05)	0.06(0.04 to 0.09)	78.46(12.4 to 179.4)
Palestine	6(4 to 9)	16(10 to 24)	162.39(79.2 to 298.05)	0.76(0.46 to 1.16)	0.72(0.47 to 1.07)	-4.33(-34.41 to 43.56)
Oman	2(1 to 3)	9(6 to 14)	456.53(218.12 to 926.73)	0.26(0.14 to 0.46)	0.5(0.32 to 0.74)	96.18(6.65 to 257.96)
Qatar	1(1 to 2)	16(10 to 24)	1219.65(767.04 to 1781.62)	1.53(0.96 to 2.26)	2.68(1.65 to 4.21)	74.88(21.23 to 139.66)
Saudi Arabia	40(21 to 66)	172(112 to 252)	330.01(132.37 to 679.17)	0.73(0.37 to 1.24)	1.14(0.74 to 1.67)	56.56(-15.89 to 168.48)
Syrian Arab Republic	32(19 to 53)	78(48 to 117)	145.72(38.98 to 323.52)	0.65(0.37 to 1.11)	0.65(0.4 to 0.97)	-0.61(-44.79 to 71.39)

Tunisia	5(3 to 9)	23(13 to 38)	325.76(137.14 to 583.2)	0.11(0.07 to 0.18)	0.18(0.1 to 0.29)	56.62(-13.52 to 153.09)
Turkey	85(54 to 130)	322(205 to 466)	281.46(164.46 to 459.37)	0.27(0.17 to 0.41)	0.36(0.23 to 0.52)	35.3(-6.22 to 103.22)
United Arab Emirates	4(3 to 6)	58(35 to 92)	1301.65(822.32 to 2022.55)	1.1(0.64 to 1.76)	1.91(1.15 to 2.96)	74.34(17.04 to 162.81)
Yemen	10(4 to 21)	24(12 to 47)	145.96(60.32 to 340.16)	0.21(0.08 to 0.49)	0.18(0.09 to 0.37)	-13.84(-43.05 to 55.98)
Afghanistan	35(19 to 62)	62(38 to 99)	78.31(16.88 to 172.33)	0.52(0.28 to 0.97)	0.65(0.38 to 1.11)	23.99(-15.86 to 81.5)
Bangladesh	78(50 to 117)	280(171 to 431)	259.67(150.29 to 430.18)	0.16(0.1 to 0.25)	0.21(0.13 to 0.32)	26.47(-12.26 to 87.59)
Bhutan	1(0 to 1)	3(2 to 4)	288.68(130.88 to 562.67)	0.3(0.16 to 0.49)	0.47(0.26 to 0.75)	55.29(-7.02 to 154.47)
India	1163(932 to 1392)	4735(3969 to 5648)	307.08(244.67 to 376.03)	0.26(0.21 to 0.31)	0.41(0.34 to 0.49)	60.26(36.4 to 87.32)
Nepal	14(8 to 21)	77(44 to 123)	456.82(246.92 to 742.88)	0.15(0.09 to 0.23)	0.34(0.19 to 0.54)	123.75(40.26 to 239.98)
Pakistan	119(91 to 154)	375(278 to 501)	213.94(123.62 to 337.35)	0.21(0.16 to 0.27)	0.3(0.22 to 0.39)	42.01(0.02 to 95.47)
Angola	33(5 to 94)	76(18 to 203)	131.29(48.8 to 337.46)	0.87(0.13 to 2.59)	0.67(0.16 to 1.78)	-22.57(-50.5 to 49.49)
Central African Republic	9(3 to 19)	12(5 to 26)	29.03(-16.01 to 85.48)	0.84(0.3 to 1.89)	0.57(0.21 to 1.34)	-32.5(-54.51 to -4.06)
Congo	11(4 to 24)	19(8 to 41)	75.46(16 to 171.53)	1.08(0.4 to 2.52)	0.74(0.3 to 1.72)	-31.08(-52.23 to 4.75)
Democratic Republic of the Congo	57(27 to 116)	113(48 to 275)	98.6(21.34 to 205.33)	0.38(0.17 to 0.87)	0.34(0.13 to 0.87)	-12.34(-47.14 to 32.75)
Equatorial Guinea	1(0 to 1)	3(2 to 5)	454.21(198.84 to 910.48)	0.33(0.16 to 0.68)	0.69(0.39 to 1.15)	106.48(13.51 to 260.54)
Gabon	5(2 to 10)	11(6 to 18)	133.69(29.87 to 318.51)	0.81(0.31 to 1.85)	1.08(0.6 to 1.82)	33.03(-25.72 to 141.08)
Burundi	13(7 to 22)	18(11 to 30)	40.27(-9.25 to 131.48)	0.57(0.32 to 0.97)	0.39(0.23 to 0.64)	-31.29(-54.83 to 9.56)
Comoros	2(1 to 3)	4(2 to 6)	139.94(52.72 to 266.44)	0.91(0.53 to 1.44)	0.85(0.49 to 1.29)	-5.9(-38.71 to 41.59)
Djibouti	1(0 to 1)	4(2 to 7)	516.6(296.15 to 881.69)	0.51(0.3 to 0.86)	0.71(0.43 to 1.13)	38.89(-10.77 to 113.44)
Eritrea	5(3 to 9)	15(8 to 24)	168.17(81.13 to 302.32)	0.55(0.32 to 0.96)	0.58(0.33 to 0.95)	4.71(-28.35 to 49.35)
Ethiopia	83(62 to 117)	145(95 to 231)	74.43(19.93 to 164.69)	0.45(0.32 to 0.65)	0.35(0.23 to 0.55)	-22.92(-45.88 to 12.02)
Kenya	34(23 to 51)	170(124 to 228)	406.6(275.04 to 605.12)	0.41(0.28 to 0.63)	0.77(0.57 to 1.02)	88.62(37.22 to 162.4)
Madagascar	21(13 to 32)	45(26 to 73)	111.43(38.94 to 209.89)	0.42(0.26 to 0.65)	0.42(0.24 to 0.66)	1.03(-32.43 to 52.91)
Malawi	18(11 to 27)	51(32 to 72)	180.75(86.9 to 324.88)	0.48(0.29 to 0.72)	0.71(0.44 to 1.03)	47.47(-0.78 to 120.4)
Mauritius	3(2 to 4)	2(1 to 2)	-45.58(-51.75 to -38.89)	0.47(0.32 to 0.64)	0.09(0.07 to 0.13)	-80.1(-82.29 to -77.85)

Mozambique	120(72 to 184)	304(154 to 564)	152.77(19.45 to 453.36)	2.41(1.45 to 3.7)	3.43(1.74 to 6.44)	42.15(-35.86 to 200.55)
Rwanda	22(13 to 33)	39(23 to 59)	75.22(14.62 to 164.42)	0.8(0.49 to 1.22)	0.66(0.4 to 1.01)	-17.46(-43.02 to 21.33)
Seychelles	0(0 to 1)	1(0 to 1)	14.03(-15.31 to 57.66)	0.87(0.59 to 1.26)	0.5(0.33 to 0.73)	-42.6(-56.64 to -21.48)
Somalia	26(12 to 48)	64(32 to 124)	144.96(61.2 to 284.89)	1.17(0.54 to 2.18)	1.2(0.58 to 2.29)	2.54(-33.26 to 58.42)
United Republic of Tanzania	81(53 to 124)	179(112 to 274)	122.63(56.42 to 224.83)	0.76(0.49 to 1.16)	0.73(0.44 to 1.09)	-4.29(-31.76 to 38.12)
Uganda	55(34 to 81)	137(85 to 211)	150.3(50.82 to 309.89)	0.88(0.54 to 1.34)	0.96(0.58 to 1.49)	9.17(-34.15 to 77.16)
Zambia	34(20 to 57)	49(20 to 115)	44.03(-26.86 to 151.32)	1.18(0.69 to 2.02)	0.78(0.34 to 1.78)	-33.95(-63.67 to 10.28)
Botswana	3(2 to 6)	11(6 to 23)	247.88(65.69 to 678.2)	0.67(0.3 to 1.31)	0.83(0.44 to 1.57)	25.05(-40.79 to 184.49)
Lesotho	6(2 to 12)	21(9 to 47)	273.66(20.4 to 1134.79)	0.71(0.26 to 1.54)	2.1(0.91 to 4.47)	195.96(1.01 to 844.26)
Namibia	2(1 to 3)	5(3 to 7)	209.09(74.12 to 434.93)	0.29(0.15 to 0.48)	0.41(0.26 to 0.62)	42.48(-21.51 to 148.72)
South Africa	113(69 to 183)	445(359 to 538)	294.03(130.84 to 575.33)	0.54(0.32 to 0.88)	1.02(0.84 to 1.23)	89.57(8.77 to 230.44)
Eswatini	3(1 to 6)	15(6 to 32)	340.03(36.57 to 1280.83)	1.27(0.55 to 2.28)	2.81(1.23 to 5.97)	121.11(-28.84 to 569.62)
Zimbabwe	51(30 to 81)	125(71 to 191)	147.08(37.7 to 310.38)	1.35(0.78 to 2.11)	2.06(1.18 to 3.22)	52.23(-12.26 to 150.46)
Benin	40(20 to 74)	86(55 to 131)	113.06(27.88 to 285.39)	2.07(0.98 to 3.79)	1.77(1.12 to 2.74)	-14.61(-49.79 to 56.99)
Burkina Faso	94(40 to 204)	177(85 to 344)	87.1(26.97 to 198.93)	2.3(0.99 to 5)	2(0.97 to 3.97)	-13.35(-42.02 to 36.23)
Cameroon	94(57 to 143)	214(109 to 340)	125.95(33.99 to 277.96)	2.26(1.31 to 3.44)	1.83(0.91 to 2.94)	-19.02(-51.54 to 32.94)
Cabo Verde	3(2 to 5)	9(5 to 13)	183.59(26.29 to 402.67)	1.29(0.75 to 2.19)	2.11(1.23 to 3.23)	62.77(-28.18 to 189.72)
Chad	40(16 to 85)	76(41 to 144)	92.3(28.04 to 213.23)	1.44(0.57 to 3.09)	1.42(0.74 to 2.73)	-1.57(-33.38 to 59.41)
C 么 te d'Ivoire	24(14 to 38)	52(26 to 83)	114.48(1.85 to 289.7)	0.66(0.39 to 1.03)	0.5(0.26 to 0.81)	-23.05(-63.77 to 39.29)
Gambia	9(6 to 14)	38(22 to 60)	316.16(133.62 to 603.65)	2.71(1.73 to 4.05)	3.99(2.18 to 6.4)	47.14(-17.25 to 146.64)
Ghana	69(38 to 122)	174(97 to 273)	153.26(11.77 to 434.58)	1.19(0.65 to 2.15)	1.16(0.63 to 1.8)	-2.68(-58.32 to 105.51)
Guinea	82(53 to 121)	133(76 to 198)	61.13(-1.29 to 168.24)	2.55(1.64 to 3.78)	2.42(1.36 to 3.61)	-5.14(-41.58 to 54.97)
Guinea-Bissau	11(5 to 19)	15(10 to 23)	34.53(-10.48 to 164.23)	2.95(1.28 to 5.15)	2.31(1.45 to 3.51)	-21.52(-47.75 to 56.06)
Liberia	27(14 to 45)	47(30 to 70)	74.62(7.3 to 174.61)	2.43(1.28 to 4.13)	2.38(1.5 to 3.62)	-2.07(-40 to 56.78)
Mali	90(58 to 134)	235(151 to 364)	160.82(83.63 to 274.57)	2.39(1.57 to 3.58)	2.87(1.83 to 4.47)	20.24(-14.42 to 70.93)

Mauritania	41(11 to 90)	63(35 to 99)	53.73(-20.02 to 279.89)	4.22(1.16 to 9.22)	3.13(1.66 to 4.95)	-25.85(-62.35 to 82.1)
Niger	48(22 to 94)	92(52 to 165)	93.2(30.55 to 227.53)	1.82(0.84 to 3.66)	1.23(0.68 to 2.16)	-32.78(-53.69 to 14.63)
Nigeria	289(137 to 575)	614(415 to 902)	112.37(25.5 to 301.06)	0.7(0.33 to 1.38)	0.75(0.52 to 1.07)	7.26(-36.61 to 102.13)
Sao Tome and Principe	0(0 to 0)	0(0 to 1)	81.88(-7.2 to 223.18)	0.3(0.2 to 0.45)	0.34(0.17 to 0.54)	10.62(-43.9 to 97.67)
Senegal	52(34 to 77)	116(73 to 175)	124.52(38.92 to 255.37)	1.67(1.09 to 2.48)	1.61(1.01 to 2.46)	-3.52(-40.86 to 55.35)
Sierra Leone	35(13 to 72)	44(27 to 71)	27.13(-29.13 to 204.49)	1.75(0.63 to 3.63)	1.23(0.74 to 1.95)	-29.72(-60.35 to 67.51)
Togo	12(8 to 19)	41(25 to 65)	236.44(96.44 to 435.11)	1.05(0.67 to 1.6)	1.22(0.72 to 1.93)	16.97(-31.45 to 88.87)
American Samoa	0(0 to 0)	0(0 to 1)	275.21(155.7 to 429.72)	0.55(0.36 to 0.82)	0.96(0.63 to 1.4)	73.74(18.59 to 142.64)
Bermuda	0(0 to 0)	0(0 to 0)	35.58(9.69 to 68.14)	0.34(0.23 to 0.45)	0.19(0.12 to 0.28)	-42.85(-53.42 to -29.97)
Cook Islands	0(0 to 0)	0(0 to 1)	149.84(67.41 to 256.82)	1.16(0.77 to 1.72)	1.33(0.87 to 1.94)	15.27(-22.33 to 65.84)
Greenland	0(0 to 0)	0(0 to 1)	150.26(67.75 to 255.43)	0.66(0.44 to 0.99)	0.81(0.48 to 1.21)	22.15(-17.38 to 75.47)
Guam	0(0 to 0)	1(1 to 2)	432.47(324.72 to 561.9)	0.34(0.23 to 0.48)	0.61(0.42 to 0.87)	82.45(42.7 to 127.76)
Monaco	0(0 to 0)	1(0 to 1)	211.3(95.02 to 427.8)	0.31(0.18 to 0.49)	0.69(0.41 to 1.05)	125.77(47.5 to 263.88)
Nauru	0(0 to 0)	0(0 to 0)	20.01(-25.55 to 90.31)	0.62(0.39 to 0.93)	0.52(0.32 to 0.82)	-16.4(-48.11 to 36.52)
Niue	0(0 to 0)	0(0 to 0)	38.77(-19.91 to 123.8)	0.44(0.26 to 0.71)	0.63(0.38 to 0.99)	44.67(-13.56 to 130.46)
Northern Mariana Islands	0(0 to 0)	0(0 to 1)	306.92(196.1 to 456.25)	0.66(0.42 to 1.01)	0.91(0.59 to 1.33)	37.14(1.21 to 81.45)
Palau	0(0 to 0)	0(0 to 0)	204.31(75.73 to 428.05)	0.55(0.33 to 0.95)	0.71(0.43 to 1.11)	27.76(-23.42 to 112.87)
Puerto Rico	11(8 to 16)	29(19 to 41)	154.17(101.09 to 219.62)	0.31(0.22 to 0.43)	0.39(0.26 to 0.55)	24.88(-0.37 to 53.97)
Saint Kitts and Nevis	0(0 to 0)	0(0 to 0)	62.83(26.33 to 109.64)	0.39(0.27 to 0.55)	0.39(0.26 to 0.56)	0.57(-19.76 to 22.91)
San Marino	0(0 to 0)	0(0 to 0)	166.7(71.88 to 315.85)	0.14(0.08 to 0.21)	0.17(0.09 to 0.29)	25.08(-20.11 to 93.22)
Tokelau	0(0 to 0)	0(0 to 0)	46.17(-5.13 to 126.59)	0.4(0.21 to 0.76)	0.51(0.28 to 0.89)	29.5(-16.57 to 101.04)
Tuvalu	0(0 to 0)	0(0 to 0)	94.97(41.69 to 184.46)	0.43(0.25 to 0.79)	0.52(0.31 to 0.84)	20.33(-14.06 to 76.83)
United States Virgin Islands	0(0 to 0)	1(0 to 1)	265.59(114.73 to 487.9)	0.22(0.13 to 0.35)	0.32(0.2 to 0.49)	45.81(-16.76 to 132.12)
South Sudan	17(10 to 27)	37(21 to 58)	115.49(48.19 to 219.13)	0.68(0.41 to 1.08)	1.01(0.6 to 1.65)	48.42(1.48 to 115.14)
Sudan	29(14 to 59)	92(53 to 145)	215.02(68.05 to 464.55)	0.33(0.15 to 0.69)	0.5(0.29 to 0.8)	53.63(-18.4 to 177.04)

Table S3. The DALYs of NRLC between 1990 and 2021 at national level, both sexes

region	Case in 1990	Case in 2021	Change in absolute number (95% UI)	ASR in 1990	ASR in 2021	change in ASR per 100 000 population (95% UI)
China	125153(100593 to 153269)	256209(194368 to 326023)	104.72(54.49 to 166.43)	13(11 to 16)	12(9 to 15)	-8.75(-30.26 to 19.66)
Democratic People's Republic of Korea	2736(1391 to 4825)	4024(2375 to 6592)	47.11(-10.59 to 171.87)	15(8 to 27)	12(7 to 19)	-21.69(-52.41 to 44.65)
Taiwan (Province of China)	2240(1635 to 3071)	8570(5868 to 11962)	282.63(200.49 to 375.89)	13(9 to 17)	21(14 to 28)	63.8(32.26 to 96.92)
Cambodia	943(436 to 1845)	2029(1001 to 3755)	115.21(37.91 to 256.73)	19(9 to 37)	15(8 to 28)	-17.54(-46.98 to 37.24)
Indonesia	9862(6149 to 14461)	31400(17330 to 48563)	218.38(128.49 to 310.04)	9(5 to 13)	12(6 to 18)	38.76(-0.13 to 78.75)
Lao People's Democratic Republic	456(276 to 737)	739(437 to 1159)	62.02(4.07 to 156.16)	20(12 to 32)	14(8 to 22)	-27.55(-53.06 to 11.57)
Malaysia	958(639 to 1386)	4587(3020 to 6647)	378.96(232.96 to 572.3)	10(7 to 14)	16(10 to 22)	58.77(10.63 to 122.91)
Maldives	12(7 to 19)	34(21 to 52)	177.4(82.56 to 330.42)	13(8 to 20)	10(6 to 15)	-21.81(-47.85 to 15.59)
Myanmar	1877(699 to 3503)	3785(1664 to 6939)	101.65(28.59 to 232.15)	7(3 to 14)	7(3 to 14)	1.31(-33.94 to 63.62)
Philippines	4692(3188 to 6498)	12456(9817 to 15699)	165.47(77.69 to 318.43)	13(9 to 19)	14(11 to 18)	4.18(-31.37 to 66.2)
Sri Lanka	494(339 to 707)	939(513 to 1536)	90.06(19.13 to 194.35)	4(3 to 6)	3(2 to 6)	-22.21(-50.84 to 18.88)
Thailand	11992(7934 to 17555)	29506(18535 to 45296)	146.04(57.98 to 253.94)	31(20 to 46)	28(18 to 42)	-10.95(-42.06 to 31.03)
Timor-Leste	25(15 to 40)	65(37 to 106)	155.36(67.72 to 287.96)	8(5 to 12)	7(4 to 12)	-9.09(-38.82 to 36.66)
Viet Nam	11248(6853 to 17592)	27547(17080 to 42393)	144.9(49.14 to 296.67)	27(16 to 42)	26(17 to 39)	-2.6(-38.92 to 55.43)

Fiji	44(26 to 70)	122(74 to 190)	180.57(63.1 to 381.74)	10(6 to 17)	15(9 to 22)	42.05(-18.25 to 137.21)
Kiribati	8(5 to 12)	16(10 to 24)	105.68(25.9 to 229.5)	18(12 to 28)	19(12 to 27)	4.97(-33.79 to 62.23)
Marshall Islands	1(1 to 3)	4(2 to 7)	199.19(79.01 to 408.87)	8(4 to 14)	11(6 to 17)	36.43(-16.72 to 125.97)
Micronesia (Federated States of)	7(4 to 11)	13(7 to 21)	91.53(19.32 to 208.08)	13(8 to 20)	16(9 to 25)	23.65(-22.43 to 96.68)
Papua New Guinea	161(68 to 403)	344(147 to 814)	114.19(37.7 to 262.65)	8(3 to 19)	6(2 to 14)	-25.73(-51.15 to 26.78)
Samoa	12(7 to 19)	20(12 to 30)	70.62(8.62 to 163.7)	13(8 to 20)	13(8 to 19)	0.87(-34.5 to 55.06)
Solomon Islands	20(7 to 46)	48(26 to 81)	143.58(31.64 to 532.53)	12(4 to 29)	11(6 to 19)	-7.11(-47.51 to 134.71)
Tonga	31(19 to 52)	52(31 to 77)	64.55(-2.35 to 175.27)	52(31 to 86)	62(37 to 93)	18.48(-30.42 to 95.75)
Vanuatu	7(4 to 14)	21(13 to 35)	200.33(91.29 to 423.03)	10(5 to 20)	11(6 to 18)	9.45(-27.64 to 83.24)
Armenia	412(275 to 602)	624(416 to 899)	51.31(8.61 to 102.79)	15(10 to 22)	15(10 to 21)	-3.58(-30.65 to 28.57)
Azerbaijan	675(362 to 1194)	1956(985 to 3669)	189.9(34.33 to 530.61)	13(7 to 23)	18(10 to 34)	42.82(-34.4 to 209.79)
Georgia	604(407 to 867)	479(320 to 684)	-20.66(-34.9 to -3.31)	9(6 to 13)	8(6 to 12)	-10.84(-26.52 to 8.2)
Kazakhstan	2557(1774 to 3629)	2091(1405 to 2981)	-18.22(-37.36 to 7.41)	19(13 to 27)	11(8 to 16)	-39.81(-53.65 to -20.31)
Kyrgyzstan	363(249 to 511)	337(213 to 508)	-7.05(-31.58 to 23.49)	12(8 to 16)	7(4 to 10)	-41.88(-56.7 to -22.64)
Mongolia	988(614 to 1562)	3125(1968 to 4877)	216.19(114.99 to 333.17)	85(52 to 132)	131(82 to 204)	54.33(5.42 to 117.3)
Tajikistan	264(166 to 413)	496(274 to 817)	87.85(3.64 to 224.33)	9(5 to 14)	8(4 to 13)	-10.96(-49.18 to 56.72)
Turkmenistan	201(146 to 278)	590(385 to 880)	193.39(106.11 to 306.12)	9(7 to 13)	13(9 to 20)	42.49(0.26 to 97.24)
Uzbekistan	789(494 to 1189)	2899(1856 to 4391)	267.68(142 to 466.1)	6(4 to 10)	10(6 to 15)	57.97(5.54 to 144.57)
Albania	555(353 to 823)	808(471 to 1310)	45.63(-16.17 to 146.46)	26(17 to 40)	19(11 to 30)	-29.18(-58.43 to 16)
Bosnia and Herzegovina	598(398 to 859)	932(578 to 1403)	55.96(10.25 to 116.35)	14(9 to 20)	15(9 to 22)	5.34(-24.86 to 43.65)
Bulgaria	2003(1311 to 2956)	966(607 to 1465)	-51.79(-67.25 to -29.06)	17(11 to 24)	7(5 to 11)	-55.47(-69.68 to -35.68)
Croatia	379(262 to 538)	626(395 to 896)	65.25(19.42 to 134.59)	6(4 to 9)	7(4 to 10)	9.86(-20.7 to 52.29)
Czechia	1173(804 to 1691)	1012(666 to 1526)	-13.73(-34.53 to 12.08)	9(6 to 12)	5(3 to 7)	-44.34(-57.82 to -27.61)
Hungary	868(601 to 1232)	721(465 to 1073)	-16.88(-39.92 to 11.53)	6(4 to 8)	4(3 to 6)	-36.26(-53.36 to -16.19)
North Macedonia	380(250 to 552)	615(373 to 924)	61.74(14.46 to 126.5)	20(13 to 29)	20(12 to 29)	-3.15(-31.12 to 34.54)

Montenegro	81(51 to 121)	160(100 to 239)	97.43(30.45 to 194.76)	13(8 to 19)	17(10 to 25)	29.25(-13.7 to 93.79)
Poland	601(515 to 700)	2684(2226 to 3164)	346.4(298.73 to 401.89)	1(1 to 2)	4(3 to 5)	181.28(150.47 to 215.65)
Romania	718(489 to 1023)	2324(1564 to 3436)	223.5(144.62 to 316.91)	3(2 to 4)	7(5 to 10)	155.54(97.13 to 225.52)
Serbia	1034(649 to 1562)	1506(915 to 2295)	45.69(0.97 to 108.68)	9(6 to 14)	9(6 to 14)	2.63(-28.04 to 46.9)
Slovakia	605(392 to 864)	730(421 to 1162)	20.73(-19.02 to 84.86)	10(7 to 14)	8(5 to 12)	-22.44(-48.29 to 16.94)
Slovenia	210(148 to 291)	437(285 to 652)	108.16(58.36 to 172.32)	9(6 to 12)	10(7 to 15)	16.68(-10.64 to 51.43)
Belarus	602(415 to 841)	876(564 to 1277)	45.65(8.05 to 94.57)	5(3 to 6)	6(4 to 8)	23.98(-8.26 to 64.89)
Estonia	126(88 to 183)	206(138 to 294)	62.62(25.84 to 107.92)	6(4 to 9)	8(6 to 12)	33.6(4.32 to 68.88)
Latvia	188(126 to 268)	239(163 to 328)	27.13(-1.18 to 63.35)	5(4 to 7)	7(5 to 9)	24.63(-3.56 to 57.27)
Lithuania	191(133 to 264)	361(241 to 517)	89.39(51.61 to 133.21)	4(3 to 6)	7(5 to 10)	61.98(29.26 to 101.83)
Republic of Moldova	224(162 to 306)	341(238 to 480)	51.87(28.67 to 81.3)	5(4 to 7)	6(4 to 8)	19.5(0.06 to 40.67)
Russian Federation	5928(5095 to 6803)	13867(11725 to 16124)	133.92(113.02 to 154.78)	3(3 to 4)	6(5 to 7)	85.35(68.88 to 101.29)
Ukraine	2665(2137 to 3267)	2201(1516 to 2902)	-17.4(-42.31 to 12.4)	4(3 to 5)	3(2 to 4)	-19.1(-43.71 to 10.09)
Brunei Darussalam	21(13 to 34)	55(35 to 84)	162.22(77.53 to 290.47)	20(11 to 33)	15(10 to 23)	-24.14(-48.14 to 13.03)
Japan	20628(17671 to 23869)	22522(17796 to 27361)	9.18(-9.07 to 27.7)	12(10 to 14)	6(5 to 7)	-48.45(-53.74 to -43.28)
Republic of Korea	15437(10307 to 22672)	20005(12991 to 30308)	29.59(-4.61 to 83.13)	50(32 to 74)	21(14 to 32)	-57.6(-68.54 to -41.75)
Singapore	193(129 to 287)	554(366 to 847)	186.84(138.26 to 240.16)	9(6 to 13)	6(4 to 10)	-24.68(-36.46 to -11.89)
Australia	701(498 to 956)	5432(3748 to 7535)	675.1(555.31 to 822.67)	4(3 to 5)	13(9 to 18)	260.26(204.89 to 325.35)
New Zealand	164(140 to 190)	813(679 to 955)	394.76(341.76 to 455.84)	4(4 to 5)	11(9 to 12)	144.28(117.57 to 173.62)
Andorra	11(6 to 17)	28(16 to 46)	168.94(63.51 to 326.39)	18(11 to 29)	19(11 to 30)	5.62(-35.27 to 67.26)
Austria	394(271 to 544)	1123(772 to 1585)	185.17(140.3 to 237.26)	3(2 to 5)	7(5 to 9)	90.91(60.76 to 124.4)
Belgium	582(380 to 838)	1243(797 to 1760)	113.8(80.74 to 148.06)	4(3 to 5)	6(4 to 8)	49.58(27.06 to 73.19)

Cyprus	36(22 to 54)	92(54 to 145)	159.07(81.89 to 280.19)	5(3 to 7)	5(3 to 7)	-0.74(-29.09 to 39.72)
Denmark	157(108 to 218)	474(303 to 679)	201.29(154.75 to 255.15)	2(1 to 3)	4(3 to 6)	96.03(66.06 to 131.89)
Finland	334(224 to 474)	778(503 to 1086)	132.51(97.33 to 175.04)	5(3 to 7)	6(4 to 9)	34.22(15.78 to 56.24)
France	5129(3421 to 7351)	12144(8006 to 17104)	136.77(100.43 to 179.2)	7(4 to 9)	10(6 to 13)	46.02(22.77 to 71.55)
Germany	5125(3552 to 7161)	12743(8726 to 17778)	148.66(112.53 to 195.46)	4(3 to 6)	7(5 to 10)	72.18(48.95 to 102.81)
Greece	444(312 to 625)	1414(984 to 1995)	218.52(177.51 to 262.33)	3(2 to 4)	6(5 to 9)	115.01(87.05 to 145.93)
Iceland	12(8 to 17)	38(26 to 54)	230.16(172.49 to 298.09)	4(3 to 6)	7(5 to 9)	63.19(34.56 to 95.97)
Ireland	122(82 to 175)	432(286 to 607)	254.94(193.62 to 316.23)	3(2 to 4)	6(4 to 8)	86.09(55.63 to 117.67)
Israel	200(135 to 285)	544(364 to 766)	171.66(130.32 to 219.48)	4(3 to 6)	5(3 to 6)	11.44(-5.89 to 31.44)
Italy	5792(4807 to 6807)	5778(4734 to 6940)	-0.25(-8.86 to 8.72)	7(6 to 8)	4(4 to 5)	-34.43(-38.9 to -29.72)
Luxembourg	25(17 to 36)	61(41 to 87)	140.06(108.85 to 178.23)	5(3 to 7)	6(4 to 8)	25.12(9.23 to 44.7)
Malta	13(8 to 18)	41(26 to 61)	219.43(164.85 to 292.05)	3(2 to 4)	4(3 to 6)	48.08(24.94 to 79.25)
Netherlands	463(324 to 630)	1728(1162 to 2410)	272.94(220.13 to 332.28)	2(2 to 3)	5(4 to 7)	115.92(86.67 to 149.1)
Norway	158(132 to 184)	522(434 to 607)	229.55(209.24 to 248.68)	3(2 to 3)	6(5 to 7)	126.77(112.51 to 140.45)
Portugal	397(274 to 553)	1589(1056 to 2273)	300.17(238.35 to 372.43)	3(2 to 4)	7(5 to 10)	147.33(108.31 to 199.21)
Spain	2530(1738 to 3567)	6093(4064 to 8931)	140.85(103.55 to 186.95)	5(3 to 7)	7(5 to 10)	44.14(23.02 to 70.07)
Sweden	665(552 to 778)	1029(822 to 1250)	54.69(33.19 to 77.18)	5(4 to 6)	5(4 to 6)	11.06(-4.43 to 28.06)
Switzerland	510(347 to 715)	923(606 to 1338)	81.08(52.71 to 113.54)	5(4 to 7)	5(4 to 8)	2.93(-13.05 to 21.42)
United Kingdom	2592(2162 to 3071)	10951(9098 to 13100)	322.49(301.64 to 342.86)	3(3 to 4)	9(8 to 11)	198.13(186.22 to 210.16)
Argentina	341(236 to 478)	1424(1004 to 1968)	317.09(248.83 to 391.73)	1(1 to 1)	3(2 to 4)	148.2(107.56 to 192.58)
Chile	343(233 to 484)	2026(1355 to 2859)	489.98(374.44 to 621.61)	3(2 to 5)	8(5 to 11)	133.15(88 to 186.08)
Uruguay	72(48 to 102)	277(183 to 394)	286(209.83 to 368.46)	2(1 to 3)	5(4 to 8)	190.52(135.46 to 251.67)
Canada	1863(1344 to 2484)	9742(6854 to 13263)	422.85(344.56 to 502.75)	6(4 to 8)	14(10 to 19)	143.2(108.38 to 179.87)
United States of America	14060(12187 to 16037)	59666(50446 to 70049)	324.35(305.4 to 343.43)	5(4 to 5)	11(9 to 12)	134.88(126.13 to 143.78)

Antigua and Barbuda	3(2 to 4)	7(5 to 9)	103.94(73.86 to 136.88)	6(4 to 8)	6(4 to 8)	-0.14(-15.44 to 15.97)
Bahamas	13(10 to 18)	34(23 to 49)	154.86(91.93 to 229.94)	8(6 to 11)	8(6 to 12)	1.12(-21.81 to 30.88)
Barbados	16(11 to 21)	34(22 to 49)	118.86(67.75 to 184.45)	5(4 to 7)	7(4 to 10)	23.26(-5.11 to 60.66)
Belize	4(3 to 6)	21(15 to 30)	413.52(293.34 to 519.43)	4(3 to 6)	7(5 to 9)	55.41(22.46 to 87.37)
Cuba	531(385 to 731)	845(579 to 1182)	59.15(28.78 to 92.75)	5(4 to 7)	4(3 to 6)	-12.5(-28.71 to 6.59)
Dominica	3(2 to 5)	8(5 to 12)	131.34(45.26 to 256.83)	6(4 to 9)	10(6 to 14)	65.81(3.9 to 153.61)
Dominican Republic	113(75 to 166)	480(306 to 715)	324.19(170.3 to 533.35)	3(2 to 4)	5(3 to 7)	68.25(9.8 to 149.91)
Grenada	3(2 to 5)	10(7 to 14)	209.07(132.06 to 296.47)	5(3 to 7)	9(6 to 13)	91.59(43.4 to 148.46)
Guyana	22(15 to 32)	42(27 to 60)	87.01(31.92 to 154.87)	5(4 to 8)	6(4 to 9)	11.57(-20.6 to 51.1)
Haiti	125(71 to 239)	243(121 to 489)	94.83(22.8 to 193.01)	4(2 to 7)	3(2 to 6)	-12.5(-43.96 to 32.91)
Jamaica	46(31 to 66)	143(95 to 206)	209.34(114.61 to 317.67)	3(2 to 4)	5(3 to 7)	80(26.53 to 142.01)
Saint Lucia	4(3 to 6)	9(6 to 13)	118.21(65.69 to 185.4)	5(3 to 6)	4(3 to 5)	-18.7(-37.9 to 4.65)
Saint Vincent and the Grenadines	5(4 to 7)	9(6 to 13)	81.31(45.46 to 122.1)	7(5 to 9)	6(4 to 9)	-6.08(-24.18 to 15.63)
Suriname	13(9 to 18)	44(27 to 67)	247.22(126.61 to 399.23)	5(3 to 7)	7(4 to 10)	43.19(-5.02 to 106.89)
Trinidad and Tobago	51(36 to 70)	131(86 to 190)	159.02(87.93 to 242.45)	6(4 to 8)	7(5 to 10)	16.61(-14.57 to 53.33)
Bolivia (Plurinational State of)	267(158 to 422)	925(529 to 1508)	246.43(124.95 to 422.75)	8(5 to 13)	10(6 to 17)	26.31(-18.48 to 89.09)
Ecuador	598(434 to 788)	1533(1058 to 2163)	156.33(95.58 to 240.43)	11(8 to 14)	9(6 to 13)	-11.85(-33.4 to 16.57)
Peru	544(365 to 800)	2166(1350 to 3205)	298.04(148.82 to 501.52)	4(3 to 6)	6(4 to 10)	49.82(-5.45 to 127.58)
Colombia	1175(869 to 1609)	3426(2304 to 4801)	191.6(131.79 to 257.24)	6(5 to 9)	6(4 to 9)	-0.67(-19.49 to 21.43)
Costa Rica	184(132 to 251)	846(578 to 1183)	361.18(273.88 to 457.99)	10(7 to 14)	15(11 to 21)	55.69(27.08 to 87.04)
El Salvador	136(96 to 186)	298(196 to 422)	118.14(64.11 to 199.39)	4(3 to 6)	5(3 to 7)	11.3(-17.79 to 52.25)
Guatemala	558(414 to 739)	1506(1039 to 2090)	169.83(125.53 to 225.42)	15(10 to 20)	13(9 to 18)	-11.35(-26.08 to 5.9)
Honduras	103(59 to 187)	629(383 to 991)	507.59(297.24 to 848.51)	5(2 to 9)	10(6 to 15)	108.95(34.18 to 227.76)
Mexico	1876(1635 to 2125)	9363(7771 to 11090)	399(339.63 to 458.8)	4(3 to 5)	7(6 to 9)	81.59(61.37 to 103.31)
Nicaragua	108(75 to 157)	397(259 to 577)	266.42(164.33 to 399.69)	6(4 to 9)	8(5 to 11)	22.67(-10.03 to 66.57)

Panama	114(82 to 154)	348(232 to 495)	205.54(140.6 to 273.47)	7(5 to 10)	8(5 to 11)	6.68(-15.71 to 31.56)
Venezuela (Bolivarian Republic of)	1170(836 to 1590)	2062(1350 to 3031)	76.22(31.2 to 134.01)	12(8 to 16)	7(5 to 10)	-39.61(-55.47 to -19.72)
Brazil	3070(2703 to 3468)	9880(8425 to 11386)	221.8(202.75 to 241.45)	3(3 to 4)	4(3 to 4)	23.88(17.42 to 30.62)
Paraguay	79(54 to 117)	354(223 to 531)	347.63(198.47 to 565.62)	3(2 to 5)	6(4 to 9)	75.88(17.1 to 158.7)
Algeria	423(282 to 629)	2062(1302 to 3037)	386.91(215.89 to 638.71)	3(2 to 5)	6(4 to 8)	78.3(16.36 to 170.82)
Bahrain	37(25 to 54)	140(88 to 220)	275.23(165.29 to 438.84)	21(13 to 31)	16(11 to 24)	-23.18(-44.6 to 7.35)
Egypt	9122(5294 to 16619)	40678(26282 to 58271)	345.95(145.43 to 657.74)	30(17 to 57)	58(37 to 83)	91.23(3.39 to 229.05)
Iran (Islamic Republic of)	1590(1251 to 2109)	7576(6267 to 8950)	376.59(278.13 to 463.32)	6(5 to 8)	10(8 to 12)	66.49(32.46 to 98.01)
Iraq	912(598 to 1347)	3621(2244 to 5263)	296.89(161.99 to 472.82)	11(7 to 16)	14(9 to 21)	35.19(-12.41 to 94.46)
Jordan	78(46 to 130)	407(246 to 604)	424.18(220.02 to 756.64)	5(3 to 9)	5(3 to 8)	-3.48(-41.67 to 59.08)
Kuwait	80(57 to 110)	124(86 to 176)	54.31(16.31 to 95.98)	11(8 to 16)	4(3 to 6)	-62.71(-71.61 to -52.68)
Lebanon	136(88 to 204)	319(208 to 476)	135.33(63.11 to 232.58)	6(4 to 9)	5(4 to 8)	-9.48(-36.96 to 27.28)
Libya	289(178 to 438)	1366(863 to 2035)	372.71(170.21 to 703.8)	14(8 to 22)	24(15 to 36)	70.9(-0.02 to 187.05)
Morocco	137(84 to 207)	555(343 to 835)	306.22(162.46 to 517.98)	1(1 to 1)	2(1 to 2)	72.67(11.78 to 167.13)
Palestine	146(89 to 228)	404(272 to 601)	175.54(89.09 to 311.61)	16(10 to 26)	15(10 to 23)	-6.23(-36.72 to 41.24)
Oman	48(26 to 85)	271(181 to 407)	469.32(242.47 to 953.02)	6(3 to 11)	12(8 to 18)	84.41(5.99 to 235.19)
Qatar	37(24 to 58)	471(288 to 731)	1156.53(724.46 to 1719.7)	32(20 to 48)	53(33 to 82)	67.02(11.99 to 135.73)
Saudi Arabia	1117(604 to 1857)	4910(3210 to 7313)	339.51(129.42 to 694.24)	17(9 to 29)	25(16 to 36)	42.07(-22.98 to 150.47)
Syrian Arab Republic	877(553 to 1438)	1991(1221 to 3049)	126.98(30.82 to 283.12)	15(9 to 25)	15(9 to 22)	-4.59(-45.58 to 63.39)
Tunisia	144(93 to 222)	568(337 to 937)	295.51(116.21 to 535.85)	3(2 to 4)	4(2 to 7)	56.96(-13.81 to 153.64)
Turkey	2159(1420 to 3183)	7211(4622 to 10620)	233.95(130.14 to 374.04)	6(4 to 9)	8(5 to 11)	27.09(-10.83 to 84.55)
United Arab Emirates	130(83 to 197)	1893(1156 to 2982)	1357.19(847.66 to 2102.93)	26(16 to 41)	42(26 to 65)	58.63(7.85 to 136.99)
Yemen	281(102 to 618)	677(347 to 1290)	140.74(56.65 to 331.67)	5(2 to 11)	4(2 to 8)	-17.53(-46.2 to 47.58)
Afghanistan	981(564 to 1668)	2003(1219 to 3200)	104.11(29.87 to 213.34)	14(8 to 23)	16(10 to 26)	20.64(-18.43 to 78.96)

Bangladesh	2321(1554 to 3413)	7394(4493 to 11425)	218.56(110.4 to 384.38)	4(3 to 7)	5(3 to 8)	18.37(-19.13 to 76.57)
Bhutan	22(12 to 36)	73(40 to 115)	223.56(91.79 to 458.5)	8(4 to 12)	11(6 to 18)	47.53(-11.49 to 149.66)
India	34407(27806 to 40764)	124114(104119 to 147676)	260.72(206.97 to 321.48)	7(5 to 8)	10(8 to 12)	50.25(28.35 to 75.27)
Nepal	417(249 to 639)	2066(1187 to 3290)	394.97(207.93 to 652.73)	4(2 to 6)	8(5 to 13)	113.38(33.92 to 220.71)
Pakistan	3663(2785 to 4698)	12462(9252 to 16630)	240.22(141.75 to 372.48)	6(4 to 7)	8(6 to 11)	44.9(3.66 to 98.68)
Angola	1045(164 to 3160)	2393(563 to 6444)	128.9(47.78 to 329.86)	22(3 to 64)	17(4 to 45)	-25.37(-52.39 to 41.55)
Central African Republic	287(111 to 606)	386(158 to 840)	34.35(-13.95 to 93.93)	22(8 to 46)	14(6 to 32)	-33.99(-56.18 to -5.97)
Congo	326(134 to 709)	594(252 to 1281)	81.95(15.74 to 189.57)	27(11 to 60)	18(8 to 40)	-32.57(-54.8 to 4.88)
Democratic Republic of the Congo	1745(852 to 3617)	3441(1528 to 8041)	97.16(20.96 to 201.63)	10(5 to 20)	8(3 to 20)	-16.58(-48.94 to 26.44)
Equatorial Guinea	18(9 to 37)	103(57 to 166)	469.31(197.71 to 978.29)	8(4 to 17)	16(9 to 27)	96.08(5.5 to 254.51)
Gabon	126(51 to 270)	307(169 to 528)	143.01(34.89 to 350.24)	21(8 to 45)	26(15 to 44)	26.1(-29.4 to 128.88)
Burundi	390(227 to 644)	577(345 to 926)	47.94(-6.48 to 144.09)	15(8 to 24)	10(6 to 16)	-34.15(-57.86 to 8.52)
Comoros	46(28 to 73)	106(63 to 162)	128.58(42.73 to 262.22)	21(13 to 33)	20(12 to 31)	-5.78(-40.07 to 43.88)
Djibouti	21(13 to 35)	120(71 to 197)	477.57(261.25 to 850.61)	13(8 to 21)	17(10 to 28)	31.8(-15.62 to 106.94)
Eritrea	175(108 to 279)	448(259 to 714)	155.55(71.32 to 292.59)	13(8 to 22)	14(8 to 22)	3.55(-29.18 to 52.31)
Ethiopia	2560(1918 to 3516)	4140(2684 to 6488)	61.72(7.25 to 156.48)	11(8 to 16)	8(5 to 13)	-26.79(-50.29 to 11.09)
Kenya	995(689 to 1508)	5076(3692 to 6849)	409.94(282.56 to 608.63)	10(7 to 16)	19(14 to 25)	84.02(35.97 to 155.22)
Madagascar	658(435 to 967)	1442(879 to 2331)	119.18(44.75 to 225.34)	11(7 to 17)	10(6 to 17)	-4.79(-37.81 to 39.88)
Malawi	541(337 to 801)	1529(989 to 2142)	182.4(86.02 to 331.16)	12(7 to 18)	17(11 to 25)	43.75(-4.63 to 118.73)
Mauritius	80(56 to 111)	41(28 to 57)	-48.53(-55.13 to -41.26)	11(7 to 15)	2(2 to 3)	-79.5(-81.86 to -77.08)
Mozambique	3011(1812 to 4633)	7804(3962 to 14304)	159.2(21.6 to 471.09)	51(31 to 78)	71(36 to 131)	38.67(-35.07 to 202.99)
Rwanda	683(400 to 1026)	1114(675 to 1672)	63.15(3.12 to 156.93)	20(12 to 31)	16(9 to 24)	-24.12(-50.14 to 15.08)
Seychelles	12(8 to 17)	14(9 to 21)	20.06(-11.72 to 68.11)	21(14 to 30)	12(8 to 17)	-44.04(-58.11 to -23)
Somalia	835(405 to 1545)	1988(1014 to 3813)	137.98(55.54 to 284.78)	29(14 to 53)	28(14 to 54)	-5.27(-37.94 to 47.59)

United Republic of Tanzania	2334(1560 to 3493)	5183(3232 to 8034)	122.08(52.15 to 232.94)	19(12 to 29)	18(11 to 27)	-7.17(-35.26 to 34.9)
Uganda	1557(962 to 2293)	4197(2577 to 6464)	169.52(61.46 to 345.38)	22(13 to 32)	23(14 to 36)	7.79(-36.06 to 75.48)
Zambia	1053(638 to 1746)	1427(562 to 3581)	35.58(-36.66 to 150.57)	31(18 to 51)	18(7 to 42)	-41.98(-70.61 to 1.32)
Botswana	95(44 to 187)	330(159 to 716)	248.35(62.43 to 691.98)	15(7 to 31)	20(10 to 40)	28.32(-39.67 to 185.71)
Lesotho	151(55 to 327)	638(261 to 1398)	321.3(28.88 to 1313.52)	17(6 to 37)	53(22 to 115)	212.38(-0.21 to 956)
Namibia	45(25 to 76)	141(89 to 208)	209.33(72.06 to 424.58)	7(3 to 11)	9(6 to 14)	43.25(-18.66 to 151)
South Africa	3525(2220 to 5477)	12216(9851 to 14884)	246.59(109.97 to 479.48)	14(9 to 23)	25(20 to 30)	72.07(2.17 to 195.35)
Eswatini	99(44 to 172)	463(171 to 1032)	367.49(37.36 to 1441.07)	30(13 to 54)	70(28 to 150)	131.27(-28.23 to 639.55)
Zimbabwe	1392(822 to 2161)	3692(2087 to 5634)	165.17(46.64 to 353.82)	31(18 to 49)	47(27 to 72)	51.17(-14.62 to 154.12)
Benin	1100(559 to 2013)	2419(1509 to 3754)	119.96(34.95 to 295.09)	51(25 to 92)	41(26 to 64)	-18.86(-50.62 to 46.74)
Burkina Faso	2627(1123 to 5629)	5036(2416 to 9758)	91.67(31.86 to 205.26)	56(24 to 123)	48(23 to 92)	-15.04(-41.75 to 34.41)
Cameroon	2730(1697 to 4029)	6275(3244 to 10259)	129.85(35.3 to 274.39)	55(33 to 82)	43(22 to 68)	-21.98(-53.84 to 30.4)
Cabo Verde	69(44 to 113)	201(121 to 297)	191(37.9 to 396.84)	30(19 to 49)	44(26 to 66)	50.04(-27.96 to 156.89)
Chad	1056(431 to 2200)	2195(1210 to 4130)	107.81(37.42 to 242.92)	35(14 to 74)	33(18 to 63)	-5.36(-36.5 to 54.1)
C 么 te d'Ivoire	748(441 to 1182)	1547(828 to 2454)	106.86(1.27 to 282.52)	16(9 to 25)	12(6 to 19)	-25.07(-64.27 to 36.16)
Gambia	275(179 to 407)	1121(644 to 1784)	307.52(130.35 to 607.88)	68(43 to 101)	98(57 to 154)	45.29(-17.56 to 147.25)
Ghana	2050(1161 to 3530)	4936(2879 to 7724)	140.85(8.6 to 409.29)	29(16 to 50)	26(15 to 41)	-7.93(-59.17 to 92.98)
Guinea	2251(1454 to 3186)	3828(2227 to 5730)	70.08(3.33 to 180.21)	64(41 to 91)	59(34 to 89)	-7.06(-43.46 to 52.19)
Guinea-Bissau	339(149 to 575)	470(303 to 740)	38.44(-8.58 to 173.73)	75(33 to 129)	54(35 to 85)	-27.44(-51.5 to 45.29)
Liberia	708(365 to 1189)	1387(871 to 2113)	95.72(22.17 to 218.27)	58(30 to 98)	55(35 to 83)	-4.25(-40.97 to 53.1)
Mali	2583(1674 to 3824)	6821(4477 to 10401)	164.08(85.65 to 279.28)	59(39 to 87)	68(44 to 105)	15.41(-18.8 to 66.3)
Mauritania	1101(302 to 2414)	1608(868 to 2433)	46.05(-20.85 to 270.35)	103(29 to 226)	70(38 to 110)	-31.68(-63.69 to 73.21)
Niger	1446(674 to 2902)	2613(1478 to 4596)	80.78(20.24 to 211.69)	45(21 to 90)	28(16 to 50)	-37.03(-57.39 to 8.2)
Nigeria	7506(3453 to 14974)	16436(10507 to 24849)	118.96(29.91 to 319.75)	16(7 to 32)	17(11 to 25)	4.24(-38.63 to 100.23)

Sao Tome and Principe	5(3 to 7)	9(5 to 16)	102.54(4.1 to 275.32)	7(5 to 10)	8(4 to 12)	8.5(-44.13 to 99.05)
Senegal	1432(953 to 2124)	3043(1952 to 4548)	112.49(34.65 to 237.51)	40(27 to 60)	37(23 to 55)	-9.32(-44.2 to 44)
Sierra Leone	913(340 to 1879)	1234(761 to 2012)	35.16(-26.34 to 225.55)	42(16 to 86)	29(17 to 47)	-31.32(-62.23 to 61.28)
Togo	366(242 to 537)	1182(724 to 1813)	222.96(94.26 to 404.22)	25(17 to 38)	28(17 to 43)	9.89(-35.87 to 73.21)
American Samoa	4(2 to 5)	12(8 to 18)	244.17(134.78 to 397.42)	14(9 to 20)	24(16 to 35)	74.96(19.95 to 149.47)
Bermuda	5(3 to 6)	5(3 to 8)	17.91(-6 to 45.2)	7(5 to 10)	4(3 to 6)	-43.07(-54.15 to -29.62)
Cook Islands	4(2 to 6)	8(5 to 13)	121.41(47.7 to 217.36)	28(18 to 42)	33(21 to 48)	17(-21.77 to 70.01)
Greenland	6(4 to 9)	12(8 to 19)	106.69(37.04 to 194.96)	16(11 to 23)	17(11 to 26)	10.41(-24.88 to 55.38)
Guam	7(5 to 10)	34(23 to 49)	387.87(285.88 to 506.25)	8(6 to 12)	17(11 to 23)	102.3(56.63 to 155.85)
Monaco	4(3 to 7)	13(8 to 20)	206.83(103.69 to 394.2)	7(4 to 11)	15(10 to 23)	124.77(48.72 to 247.01)
Nauru	1(1 to 1)	1(1 to 2)	24.33(-23.77 to 101.23)	17(11 to 25)	16(9 to 25)	-5.5(-41.1 to 52.03)
Niue	0(0 to 0)	0(0 to 1)	44.59(-16.02 to 140.29)	11(7 to 18)	16(9 to 25)	42.29(-17.62 to 136.4)
Northern Mariana Islands	4(2 to 6)	12(8 to 18)	236.05(132.17 to 390.39)	16(11 to 24)	22(14 to 31)	34.29(1.23 to 78.36)
Palau	2(1 to 3)	5(3 to 8)	218.77(86.89 to 451.18)	15(8 to 25)	20(11 to 31)	33.32(-20.02 to 127.61)
Puerto Rico	265(190 to 362)	585(392 to 812)	120.77(74.24 to 179.01)	7(5 to 10)	9(6 to 13)	26.5(-0.31 to 58.02)
Saint Kitts and Nevis	3(2 to 5)	6(4 to 9)	82.67(39.4 to 134.93)	9(6 to 12)	9(6 to 12)	-3.82(-24.46 to 18.02)
San Marino	1(1 to 2)	3(1 to 4)	145.51(54.34 to 282.04)	3(2 to 4)	4(2 to 6)	26.64(-21.42 to 97.35)
Tokelau	0(0 to 0)	0(0 to 0)	49.14(-4.54 to 133.18)	10(5 to 18)	13(7 to 23)	36.38(-13.14 to 114.34)
Tuvalu	1(0 to 1)	1(1 to 2)	85.83(34.5 to 171.32)	11(6 to 19)	13(8 to 21)	20.68(-13.47 to 75.99)
United States Virgin Islands	4(3 to 6)	13(8 to 21)	217.19(84.9 to 417.03)	5(3 to 7)	7(5 to 12)	55.03(-11.25 to 147.85)
South Sudan	485(284 to 798)	1105(646 to 1736)	127.82(52.13 to 241.04)	17(10 to 28)	25(14 to 39)	44.5(-3.56 to 113.85)
Sudan	819(413 to 1582)	2574(1499 to 4098)	214.34(68.65 to 460.01)	8(4 to 16)	12(7 to 18)	46.26(-22.74 to 160.97)
